# Minimizing the distortions in electrophysiological source imaging of cortical oscillatory activity *via* Spectral Structured Sparse Bayesian Learning

**DOI:** 10.3389/fnins.2023.978527

**Published:** 2023-03-15

**Authors:** Deirel Paz-Linares, Eduardo Gonzalez-Moreira, Ariosky Areces-Gonzalez, Ying Wang, Min Li, Mayrim Vega-Hernandez, Qing Wang, Jorge Bosch-Bayard, Maria L. Bringas-Vega, Eduardo Martinez-Montes, Mitchel J. Valdes-Sosa, Pedro A. Valdes-Sosa

**Affiliations:** ^1^MOE Key Lab for Neuroinformation, The Clinical Hospital of Chengdu Brain Science Institute, University of Electronic Science and Technology of China, Chengdu, China; ^2^Neuroinformatics Department, Cuban Neuroscience Center, Havana, Cuba; ^3^Center for Biomedical Imaging and Neuromodulation, Nathan Kline Institute for Psychiatric Research, Orangeburg, NY, United States; ^4^Research Unit for Neurodevelopment, Institute of Neurobiology, Autonomous University of Mexico, Querétaro, Mexico; ^5^Faculty of Electrical Engineering, Central University “Marta Abreu” of Las Villas, Santa Clara, Cuba; ^6^Faculty of Technical Sciences, University of Pinar del Río “Hermanos Saiz Montes de Oca”, Pinar del Rio, Cuba; ^7^McGill Centre for Integrative Neurosciences MCIN, Montreal Neurological Institute, McGill University, Montreal, QC, Canada; ^8^Ludmer Centre for Mental Health, Montreal Neurological Institute, McGill University, Montreal, QC, Canada

**Keywords:** electrophysiological source imaging, Fourier transform, cross-spectrum, *joint a priori* probability, structured sparsity, *maximum a posteriori* probability, Bayesian learning

## Abstract

Oscillatory processes at all spatial scales and on all frequencies underpin brain function. Electrophysiological Source Imaging (ESI) is the data-driven brain imaging modality that provides the inverse solutions to the source processes of the EEG, MEG, or ECoG data. This study aimed to carry out an ESI of the source cross-spectrum while controlling common distortions of the estimates. As with all ESI-related problems under realistic settings, the main obstacle we faced is a severely ill-conditioned and high-dimensional inverse problem. Therefore, we opted for Bayesian inverse solutions that posited *a priori* probabilities on the source process. Indeed, rigorously specifying both the likelihoods and *a priori* probabilities of the problem leads to the proper Bayesian inverse problem of cross-spectral matrices. These inverse solutions are our formal definition for cross-spectral ESI (cESI), which requires *a priori* of the source cross-spectrum to counter the severe ill-condition and high-dimensionality of matrices. However, inverse solutions for this problem were NP-hard to tackle or approximated within iterations with bad-conditioned matrices in the standard ESI setup. We introduce cESI with a *joint a priori* probability upon the source cross-spectrum to avoid these problems. cESI inverse solutions are low-dimensional ones for the set of random vector instances and not random matrices. We achieved cESI inverse solutions through the variational approximations *via* our Spectral Structured Sparse Bayesian Learning (ssSBL) algorithm https://github.com/CCC-members/Spectral-Structured-Sparse-Bayesian-Learning. We compared low-density EEG (10–20 system) ssSBL inverse solutions with reference cESIs for two experiments: (a) high-density MEG that were used to simulate EEG and (b) high-density macaque ECoG that were recorded simultaneously with EEG. The ssSBL resulted in two orders of magnitude with less distortion than the state-of-the-art ESI methods. Our cESI toolbox, including the ssSBL method, is available at https://github.com/CCC-members/BC-VARETA_Toolbox.

## Introduction

Brain function is embodied in large-scale dynamic networks underlying all behavior and cognition. The natural modes of these networks are oscillations at functionally specific frequencies (Engel et al., 2001; Varela et al., 2001). Direct (invasive) observations of brain oscillations are increasingly more fine-grained and informative (Buzsáki et al., 2013; Frauscher et al., 2018). However, they cannot be feasibly examined in the whole brain *in vivo* human studies. Hence, considerable effort is dedicated to developing indirect (non-invasive) imaging methods to reveal the brain's oscillatory architecture with fine-grained time resolution. Notably, such an imaging modality should reveal the two different but interrelated aspects of networks: (a) the *activity* of each dynamical unit (node) and (b) their *connectivity*.

Electrophysiological Source Imaging (ESI) is a prime candidate for mapping brain network activity and connectivity. ESI is predicated upon the fact that *the Electro-encephalogram* (EEG), *Magneto-encephalogram* (MEG), or *Electrocorticogram* (ECoG) are generated by the electrical currents of macroscopic neural masses (nodes) resulting from the local mean field of post-synaptic potential activity (Jirsa and Haken, 1996, 1997; Jirsa, 2004; Deco et al., 2008; Friston, 2009; Daunizeau et al., 2010, 2011; Moran et al., 2013).

In addition, ESI has been used in attempts to estimate these currents, also known as source activity, from their electrophysiological observables. For a neural mass, the current is directly proportional to the mean field activity (Valdes-Sosa et al., 2009; Rosa et al., 2010). In turn, each neural mass is the collection of neurons to the extent of a few millimeters such that a mean-field observable (Freeman, 1975; Vinck and Perrenoud, 2019) is the “source” of the observations. In this study, we attempted the most general formulation of the problem as a reference for future work while providing concrete examples.

In principle, source activity in a network is modeled as a strictly dynamic random field ***ι***(***ϱ*** , ***ς***) over a continuous spatiotemporal manifold with ***ϱ*** ∈ ℝ^3^ (the parts of the brain that generate observations) and continuous time ***ς*** ∈ ℝ. In practice, the random field ***ι***(***ϱ*** , ***ς***) must be discretized at spatial points ***ϱ***_*g*_; *g* = 1, ⋯ , *G*, and at discrete time points ***ς***_*t*_ = *t* △ ***ς***; *t* = −*T*, ⋯ , *T*. In this scenario △***ς*** is the sampling period. Thus, we focused on the vector time series ***ι***(*t*), defined as the vector function with entries ***ι***(*g* , *t*) = ***ι***(***ϱ***_*g*_ , ***ς***_*t*_). The (multi-channel) electro-physiological data is the vector time series ***v***(*t*) with entries ***v***(*e* , *t*) for each sensor, *e* = 1⋯*E* that arises from the discretization of *v*(***ϱ*** , ***ς***), where the electromagnetic field was produced by ***ι***(***ϱ***, ***ς***).

The data ***v***(*t*) from its latent source activity ***ι***(*t*) is presented in the following forward model (Eq. 1).

(1) $$\begin{array}{l} v\left(t\right)={\text{L}}_{v\iota }\iota \left(t\right)+\xi \left(t\right) \end{array}$$

Where **L**_***vι***_ is the real-valued lead field matrix or forward operator that projects sources ***ι***(*t*) to forward the data ***v***(*t*), and ***ξ***(*t*) is the time series of instrumental noise assumed to be independent of the source activity ***ι***(*t*). The forward operator (lead field) **L**_***vι***_ is linear and stationary by definition, derived from the discretization of a quasi-static electromagnetic forward model (Hämäläinen et al., 1993; Riera and Fuentes, 1998; Hallez et al., 2007; Lei et al., 2011; Piastra et al., 2020). For operators, we used suffixes that indicate the operator's codomain and domain.

ESI can be defined as the generally non-linear inverse solution (Eq. 2) (Nunez, 1974; Hämäläinen and Ilmoniemi, 1994; Nunez et al., 1994; Baillet et al., 2001; Nunez and Srinivasan, 2006; Burle et al., 2015) *via* the optimal inverse operator that we denote with a hat ${\hat{\text{W}}}_{\iota v}$. An inverse operator ${\hat{\text{W}}}_{\iota v}$ projects the data ***v***(*t*) to explain approximately its source and produce its estimator $\hat{\iota }\left(t\;\right)$.

(2) $$\begin{array}{l} \hat{\iota }\left(t\right)\leftarrow {\hat{\text{W}}}_{\iota v}\left(v\;\left(t\right)\right) \end{array}$$

Optimizing **W**_***ιv***_ from the data solves an inverse problem, Eq. (1), that is not only ill-posed in the sense of Hadamard (Hadamard and Morse, 1953) with degeneracy in a (*G* − *S*)-dimensional space but also severely ill-conditioned and high dimensional (with *G* ≫ *E*). Overcoming these challenges to obtain acceptable inverse solutions has been the subject of much research in specific optimization methods for **W**_***ιv***_ which was well-summarized in the study of Knösche and Haueisen (2022).

Although we have defined ESI in terms of time domain signals, it is well-known that brain activity is oscillatory at all scales, from the local field potentials at the neuronal level (Freeman, 1975; Vinck and Perrenoud, 2019) to the EEG, MEG, or ECoG (Niedermeyer and da Silva, 2005; Le Van Quyen and Bragin, 2007; Frauscher et al., 2018). In tailoring ESI for oscillatory activity, a natural framework is that of the frequency domain ***ι***(*f*), a random vector representing the (discrete) Fourier transform of vector time series ***ι***(*t*), and comprising complex-valued entries ***ι***(*g* , *f*) for each source *g* = 1, ⋯ , *G* and frequency *f* = −*T*, ⋯ , *T*. Considering that we may compute the physical frequency as ν_*f*_ = *f△ν* for a spectral period $△\nu =\frac{1}{\left(\left(2\;T\;+\;1\right)\;△\;\varsigma \right)}$ where (2 *T* + 1)△***ς*** is the Nyquist frequency, the corresponding frequency domain data term is ***v***(*f*).

Then, the equivalent expressions to the previous Eqs. (1) and (2) in the frequency domain are Eq. (3), the corresponding inverse problem for the Fourier transform ***ι***(*f*), and its inverse solution leading to the estimator $\hat{\iota }\left(f\right)$. Considering that solving an inverse problem in the frequency domain should be (ideally) optimizing the inverse operator ${\hat{\text{W}}}_{\iota v}$ Eq. (3) from the data Fourier transform ***v***(*f*) (and not from the process ***v***(*t*)).

(3) $$\begin{array}{l} \begin{array}{c} v\left(f\right)={\text{L}}_{v\iota }\iota \left(f\right)+\xi \left(f\right)\\ \hat{\iota }\left(f\right)\leftarrow {\hat{\text{W}}}_{\iota v}\left(v\;\left(f\right)\right) \end{array} \end{array}$$

Frequency domain descriptions of the electrophysiological observations and their inverse solutions (Eqs. 1–3) have been used fruitfully to probe behavior and cognition (Valdés et al., 1992; Engel et al., 2001; Varela et al., 2001; Le Van Quyen and Bragin, 2007; Marzetti et al., 2008; Valdes-Sosa et al., 2009; Brookes et al., 2011a,b; Faes and Nollo, 2011; Faes et al., 2012, 2017; Friston et al., 2012; Hipp et al., 2012; Colclough et al., 2015; Wens et al., 2015; Mahjoory et al., 2017; Vidaurre et al., 2018a,b; Tewarie et al., 2019; Nolte et al., 2020).

Our concern, then, is with frequency domain ESI in Eq. (3), our primary target being the source cross-spectral density matrix or source cross-spectrum $\Sigma_{\iota \iota }\left(f\right)=\langle \iota \;\left(f\right)\;\iota^{†}\;\left(f\right)\rangle$, with the expected value over the sample space of the discrete Fourier transform ***ι***(*f*) (Valdés et al., 1992; Engel et al., 2001; Varela et al., 2001; Nunez and Srinivasan, 2006; Hipp et al., 2012; Vidaurre et al., 2018b). The corresponding data cross-spectrum is $\Sigma_{vv}\left(f\right)=\langle v\;\left(f\right)\;v^{†}\;\left(f\right)\rangle$. In practice, this later quantity must be substituted by its estimator ${\bar{\Sigma }}_{vv}\left(f\right)$ Eq. (4).

(4) $$\begin{array}{l} {\bar{\Sigma }}_{vv}\left(f\right)=\langle v_{m}\;\left(f\right)\;v_{m}^{†}\;\left(f\right);\;M\rangle =\frac{1}{M}\overset{M}{\underset{m=1}{\sum }}v_{m}\left(f\right)v_{m}^{†}\left(f\right) \end{array}$$

${\bar{\Sigma }}_{vv}\left(f\right)$ is denoted with a different hat type since the expectation is for a finite number of instances *M* with an index *m* = 1⋯*M*. Toward this estimation, we followed the standard practice of segmenting the data time series ***v***(*t*) into segments (***v***_*m*_ (*t*); ∀ *m*). Thus, we worked with instances (***v***_*m*_ (*f*); ∀ *m*) of the discrete Fourier transform applied to realizations or observations for these segments.

The frequency domain inverse problem and the inverse solution for the source cross-spectrum **Σ**_***ιι***_(*f*) may be stated as Eq. (5) (He et al., 2019), valid under the condition of independence between the source process ***ι***(*t*) and the noise process **ξ**(*t*) in the previous forward model (Eq. 1).

(5) $$\begin{array}{l} \begin{array}{c} \Sigma_{vv}\left(f\right)={\text{L}}_{v\iota }\Sigma_{\iota \iota }\left(f\right){\text{L}}_{\iota v}+\Sigma_{\xi \xi }\left(f\right)\\ {\hat{\Sigma }}_{\iota \iota }\left(f\right)\leftarrow {\hat{\text{W}}}_{\iota v}\left({\bar{\Sigma }}_{vv}\;\left(f\right)\right) \end{array} \end{array}$$

The pursuit of the cross-spectrum **Σ**_***ιι***_(*f*) is rewarding since it completely specifies the multivariate linear properties of any stochastically driven system, be it linear or non-linear (Brillinger, 2001, 2012), though the latter requires additional higher-order kernels for a complete description (Brillinger, 1965; Brillinger and Rosenblatt, 1967). We used the asymptotic stochastic properties of the discrete Fourier transform to introduce these developments (Section Asymptotic probability theory of the Fourier transform and cross-spectrum).

Cross-spectral diagonal elements **Σ**_***ιι***_(*g* , *g* , *f*) have intuitive interpretations: the variances $\sigma_{\iota \iota }^{2}\left(g\;,\;f\right)$ ($\sigma_{\iota \iota }^{2}\left(f\right)=diag\left(\Sigma_{\iota \iota }\;\left(f\right)\right)$) are the spectra of source activity, the *Cortical Spectral Topography* (CST). Off-diagonal elements $\Sigma_{\iota \iota }\left(g\;,\;g{\;}^{\prime },\;f\right)$ are the cross-spectra reflecting functional connectivity. Optimal inverse operators ${\hat{\text{W}}}_{\iota v}$ for the cross-spectrum **Σ**_***ιι***_(*f*) Eq. (5) is a novel form of electrophysiological source imaging: *cross-spectral ESI* (cESI).

From expressions (Eqs. 1–5), the time and frequency domain variants of the inverse problem, it is evident that cESI, the inverse solution ${\hat{\Sigma }}_{\iota \iota }\left(f\right)$ of the source cross-spectrum **Σ**_***ιι***_(*f*), may be obtained from three different types of primary information: ***v***(*t*), or ***v***(*f*), or ${\bar{\Sigma }}_{vv}\left(f\right)$. Initially, it might seem that an inverse solution ${\hat{\Sigma }}_{\iota \iota }\left(f\right)$ from any of these data types would be equivalent. However, as shown further into the study, each data type requires optimizing its specific inverse operator ${\hat{\text{W}}}_{\iota v}$, thus defining different estimation “routes” to cross-spectrum **Σ**_***ιι***_(*f*).

We describe in detail the theory, benefits, and problems of each route, in terms of the forward operator **L**_***vι***_ (Section Forward projection by the lead field linear and stationary operator), and the inverse operator **W**_***ιv***_ (Section Bayesian (MAP) inverse operators. Quasilinear (F-invariant) approximation).

In order to explore these routes in the following sections (Sections Bayesian MAP1 inverse operators. MNE, eLORETA, and LCMV as particular cases and Data used for the validation), we need to select an inverse solution framework, of which there are many potentially valuable approaches (Knösche and Haueisen, 2022). We adopted the Bayesian *maximum a posteriori* (MAP) probability approach (MacKay, 2003). The MAPs (for each route) are derived from finding the latent variables X that maximize the *posterior* probability q(X|Y)(Eq. 6).

(6) $$\begin{array}{l} q\left(X|Y\right)\propto p\left(Y|X\right)p\left(X\right) \end{array}$$

Where p(Y|X) is the *likelihood* of the data Y conditional on the latent variable X. In our context, Y can corresponding to be ***v***(*t*), or ***v***(*f*), or ${\bar{\Sigma }}_{vv}\left(f\right)$, and X can be corresponding to ***ι***(*t*), or ***ι***(*f*), or **Σ**_***ιι***_(*f*) (Grave de Peralta Menendez et al., 2004; Friston et al., 2006; Mattout et al., 2006; Friston K. J. et al., 2008; Wipf and Nagarajan, 2009). The term p(X) is the respective *a priori* probability upon the latent variable ***ι***(*t*), or ***ι***(*f*), or **Σ**_***ιι***_(*f*).

As can be observed in the following section (Section Data used for the validation), the third route inverse solution (Eq. 5) has desirable properties for cESI. However, if not impossible in a realistic cESI setup, such inverse solutions are N-P hard and therefore require Approximated Bayesian Computation (ABC) (Csilléry et al., 2010). This issue has been discussed in detail in the Bayesian literature (Dempster et al., 1977; Liu and Rubin, 1994; Daunizeau and Friston, 2007; Friston et al., 2007; Nummenmaa et al., 2007; Friston K. J. et al., 2008; Paz-Linares et al., 2018).

In this study, we took a more practical path. Rather than attempting to use complicated ABCs to obtain more general solutions, the cESI rationale we focused on is to restrict Bayesian inverse operators **W**_***ιv***_ in Eqs. (1–5) within the “quasilinear” class **W**_***ιv***_ ≅ **T**_***ιv***_. Quasilinear inverse operators are also known in a more general context as the linear proximal operators or as linear back projectors that solve non-linear optimization or inverse problems (Kaplan and Tichatschke, 1998; Piotrowski and Yamada, 2008; Gramfort et al., 2012; Tirer and Giryes, 2020).

Here, quasilinear inverse operator **T**_***vι***_, which holds the same linearity attribute as the forward operator **L**_***vι***_, produced cESI that preserved the amplitude and phase information in the frequency domain (Mantini et al., 2007; Marzetti et al., 2008; Brookes et al., 2011b; Hipp et al., 2012; Lopes da Silva, 2013; He et al., 2019; Nolte et al., 2020). That is, we ensured that **T**_***vι***_ possesses the attribute of what we call “F-invariance” for essential properties (Brillinger, 2001, 2012).

An immediate consequence of taking **W**_***ιv***_ ≅ **T**_***ιv***_, a quasilinear approximation of the inverse operator in the third cESI route (Eq. 5), is the following representation of source cross-spectrum $\Sigma_{\iota \iota }\left(f\right)≅{\bar{\Sigma }}_{\iota \iota }\left(f\right)$ (Eq. 7). Here, we considered ${\bar{\Sigma }}_{\iota \iota }\left(f\right)$ to be calculated from the set of random instances (***ι***_*m*_ (*f*); ∀ *m*) of the latent vector process ***ι***(*f*).

(7) $$\begin{array}{l} {\bar{\Sigma }}_{\iota \iota }\left(f\right)=\langle \iota_{m}\left(f\right)\iota_{m}^{†}\left(f\right);M\rangle =\frac{1}{M}\overset{M}{\underset{m=1}{\sum }}\iota_{m}\left(f\right)\iota_{m}^{†}\left(f\right) \end{array}$$

Once $\Sigma_{\iota \iota }\left(f\right)≅{\bar{\Sigma }}_{\iota \iota }\left(f\right)$ was assumed, the ABCs were simplified dramatically since a MAP for the matrix ${\bar{\Sigma }}_{\iota \iota }\left(f\right)$ (Eq. 7) turns into a joint-MAP (Section Validation rationale) for the random vectors (***ι***_*m*_ (*f*); ∀ *m*) that are implicit in ${\bar{\Sigma }}_{\iota \iota }\left(f\right)$ (Hsiao et al., 1998, 2002; Yeredor, 2000; Davis et al., 2001; Auranen et al., 2005; Wipf and Nagarajan, 2009; Chen et al., 2011).

We implemenedt the joint-MAP *via* Spectral Structured Sparse Bayesian Learning (ssSBL), the type of ABC developed in Section Measures of distortion. As shown under realistic inverse problem settings (Section Results), the third cESI route (Eq. 5) implemented *via* the ssSBL approach leads to less distorted estimates than the traditional methods for the first and second cESI routes (Eqs. 1–3). To judge distortions, we employed the well-known ESI methods as a baseline: *Exact Low-Resolution Electromagnetic Tomographic Analysis* (ELORETA) (Pascual-Marqui et al., 2006) and *Linearly Constrained Minimum Variance* (LCMV) (Van Veen et al., 1997).

The theoretical framework allowed one to consider the fundamental problem of ESI distortions. Indeed, significant distortions are expected with any state-of-the-art inverse solutions in a realistic ESI setup. The distortions, which we explore later, are localization error and leakage (blurring). These distortions are pervasive comparing simulated topographic vectors, say ***ι***(*t*) or ***ι***(*f*), vs. their inverse solution $\hat{\iota }\left(t\right)$ or $\hat{\iota }\left(f\right)$ (Kobayashi et al., 2003; Grova et al., 2008; Schoffelen and Gross, 2009; Haufe et al., 2013; Burle et al., 2015; Colclough et al., 2015; Bradley et al., 2016; Mahjoory et al., 2017; Stokes and Purdon, 2017; He et al., 2018, 2019; Palva et al., 2018; Haufe and Ewald, 2019; Marinazzo et al., 2019).

As we will show in Section Discussion, the topographic distortions of the inverse solutions for a random vector ***ι***(*t*) or ***ι***(*f*) can reach unacceptable levels for second-order statistics, such as the sample estimator for the cross-spectrum ${\bar{\Sigma }}_{\hat{\iota }\hat{\iota }}\left(f\right)$ calculated from an inverse solution $\hat{\iota }\left(t\right)$ or $\hat{\iota }\left(f\right)$. Minimizing CST distortions (for the estimator of the spectrum $\sigma_{\iota \iota }^{2}\left(f\right)$) benefits the overall cross-spectral estimation (for **Σ**_***ιι***_(*f*)). Our results suggest that that ssSBL significantly reduces these distortions.

## Standard cESI theory

### Asymptotic probability theory of the Fourier transform and cross-spectrum

The material in this section (with minor differences in notation) is described in greater detail in Brillinger (2012). For an exhaustive definition of terms, refer to Supplementary material (SD, Section Introduction). Our primary interest will be in frequency domain quantities. Let **x**(*t*) be an *R*-dimensional vector time series. We worked with the following definitions:

- The *vector stochastic process* **x**(*f*) (Eq. 8) is defined in the discrete frequency domain ν_*f*_ = *f* △ *ν* with *f* = −*T*, ⋯ , *T*, as the discrete Fourier transform of the vector time series **x**(*t*).

(8) $$\begin{array}{l} \text{x}\left(f\right)=\overset{T}{\underset{t=-T}{\sum }}\text{x}\left(t\right)e^{-i2\pi \left(f\;△\;\nu \right)\left(t\;△\;\varsigma \right)} \end{array}$$

- The *cross-spectral density matrix* or *cross-spectrum* **Σ**_**xx**_(ν) (Eq. 9) was defined in the frequency domain ν as the Fourier transform of the *auto-covariance matrix* **Σ**_**xx**_(τ).

(9) $$\begin{array}{l} \Sigma_{\text{x}\text{x}}\left(\nu \right)=\overset{+\infty }{\underset{\tau =-\infty }{\sum }}\Sigma_{\text{x}\text{x}}\left(\tau \right)e^{-i2\pi \nu \left(\tau \;△\;\varsigma \right)} \end{array}$$

Here, the *auto-covariance* $\Sigma_{\text{x}\text{x}}\left(\tau \right)=\langle \text{x}\;\left(t\right)\;{\text{x}}^{†}\;\left(t\;+\;\tau \right)\rangle$ depends on the time-lag τ and does not vary with time *t*, thus being second-order stationary. We will furthermore assume (for technical reasons) that **x**(*t*) is a *strictly stationary vector time series* where all moments are also translation invariants. We also assumed that the strong mixing condition holds. This condition was due to the rapid decrease in the magnitude of the autocovariance **Σ**_**xx**_(τ), and all higher-order moments as the time-lag τ increases.

A fundamental result on which we based our work is Theorem 4.1.1 of Brillinger (2001), which can be understood as the equivalent for Fourier coefficients of the central limit theorem under stationarity and strong mixing conditions (Rosenblatt, 1956). In our notation, this theorem states:

“Assume that the number of time points in the discrete Fourier transform (Eq. 8), goes to the infinity (*T* → +∞), and let the sampling period go to zero (△***ς*** → 0+) so that the spectral resolution $△\nu =\frac{1}{\left(\left(2\;T\;+\;1\right)\;△\;\varsigma \right)}=constant$ holds constant. Then, it holds that **x**(*f*) is asymptotically independent for all *f* and converges in probability to the *circularly symmetric multivariate complex-valued Gaussian probability density* (Eq. 10). Where the Hermitian covariance matrix **Σ**_**xx**_(*f*) is the cross-spectrum (Eq. 9) at the frequencies ν_*f*_ = *f* △ *ν* with *f* = −*T*, ⋯ , *T*.”

(10) $$\begin{array}{l} N^{\mathbb{C}}\left(\text{x}\left(f\right)|0,\Sigma_{\text{x}\text{x}}\left(f\right)\right)=\frac{1}{\left|\pi \;\Sigma_{\text{x}\text{x}}\;\left(f\right)\right|}e^{-{\text{x}}^{†}\left(f\right)\Sigma_{\text{x}\text{x}}^{-1}\left(f\right)\text{x}\left(f\right)} \end{array}$$

We emphasize that this Gaussian distribution asymptotic distribution not only holds for **x**(*f*) but also for time-varying estimators such as the time-windowed discrete Fourier transform or the Hilbert transform **x**(*t* , *f*) (Bruns, 2004). The latter has been widely used in the literature (Faes and Nollo, 2011; Friston et al., 2012; Nolte et al., 2020).

It is important to note that Gaussianity might not be valid for the original time series **x**(*t*) or even one of its band-filtered versions, as many assume in the literature (Grave de Peralta Menendez et al., 2004; Friston et al., 2006; Mattout et al., 2006; Friston K. J. et al., 2008; Wipf and Nagarajan, 2009; Paz-Linares et al., 2017). In fact, even in the case of non-Gaussian, non-linear time series Brillinger's theorem is valid as long as stationarity and strong mixing hold. This validity does not imply that the spectral density matrix **Σ**_**xx**_(*f*) completely characterizes the non-linear or non-Gaussian system. Cumulant information of orders higher than two may be necessary for a complete system description (Brillinger, 1965; Brillinger and Rosenblatt, 1967).

Brillinger's theorem leads to the probability density of any sampled estimator of the cross-spectrum. In particular, it applies to ${\bar{\Sigma }}_{\text{x}\text{x}}\left(f\right)=\langle {\text{x}}_{m}\;\left(f\right)\;{\text{x}}_{m}^{†}\;\left(f\right);\;M\rangle$ the sampled estimator for Fourier transform instances (**x**_*m*_ (*f*); ∀ *m*) with sample size *M*. Here, these instances (**x**_*m*_ (*f*); ∀ *m*) represent the discrete Fourier transform applied to sample realizations (**x**_*m*_ (*t*); ∀ *m*) obtained from time segments of the observations. Then, it follows that a Hermitian Wishart *W*^ℂ^ (Eq. 11), with *R*-dimensional scale matrix **Σ**_**xx**_(*f*) (cross-spectrum), and degree of freedom *M* (with *M* ⩾ *R*), is the probability density of the estimator ${\bar{\Sigma }}_{\text{x}\text{x}}\left(f\;\right)$.

(11) $$\begin{array}{l} W^{\mathbb{C}}\left({\bar{\Sigma }}_{\text{x}\text{x}}\left(f\right)|\Sigma_{\text{x}\text{x}}\left(f\right),M\right)\propto \frac{|{\bar{\Sigma }}_{\text{x}\text{x}}\;\left(f\right){|}^{M-R}}{|\Sigma_{\text{x}\text{x}}\;\left(f\right){|}^{M}}e^{-Mtr\left(\Sigma_{\text{x}\text{x}}^{-1}\left(f\right){\bar{\Sigma }}_{\text{x}\text{x}}\left(f\right)\right)} \end{array}$$

Since we based our further developments on the assumption of Gaussianity (Eqs. 10, 11), we carried out the statistical test for the distribution of the discrete Fourier transform **x**(*f*) (Eq. 10) with two examples of resting state sensor data: MEG data from the Human Connectome Project (HCP) (Van Essen et al., 2013) and Macaque ECoG data from the Neurotycho project (Nagasaka et al., 2011). The outcome of this test for data did not allow us to reject the hypothesis of Gaussianity, which then was also plausible for the sources.

### Forward projection by the lead field linear and stationary operator

We emphasize that linearity and stationarity are essential “conservative” attributes of the operators for our target (cESI). We note, this is the reason why one can state all the forward “routes” in terms of the same operator **L**_***vι***_ (Eq. 12). Preserving the Gaussianity of the Fourier transform ***ι***(*f*) is only possible under the linear forward operator **L**_***vι***_ and subsequent linear or quasilinear inverse operator **T**_***ιv***_ (Marzetti et al., 2008; He et al., 2019; Nolte et al., 2020).

(12) $$\begin{array}{l} \begin{array}{c} \left(route\;1\right)\;\text{v}\left(t\right)={\text{L}}_{v\iota }\iota \left(t\right)+\xi \left(t\right)\\ \left(route\;2\right)\;\text{v}\left(f\right)={\text{L}}_{v\iota }\iota \left(f\right)+\xi \left(f\right)\\ \left(route\;3\right)\;\Sigma_{vv}\left(f\right)={\text{L}}_{v\iota }\Sigma_{\iota \iota }\left(f\right){\text{L}}_{\iota v}+\Sigma_{\xi \xi }\left(f\right) \end{array} \end{array}$$

Furthermore, both attributes are crucial to avoid non-linear warping and delays of the amplitude and phase information in the frequency domain for cESI (Reid et al., 2019). Therefore, we define operators with these properties for the frequency domain as “F-invariant.” Though we restricted our attention later to F-invariant operators **T**_***ιv***_, non-linear operators **W**_***ιv***_ have been useful in other contexts (Picton and Hillyard, 1974; Picton et al., 1974; Lopes da Silva et al., 1991; Clark et al., 1994; Makeig et al., 1999, 2004; Makeig, 2002; Eichele et al., 2005; Harrison et al., 2008; Vega-Hernández et al., 2008; Maurer and Dierks, 2012).

Noteworthily, distinguishing forward “routes” leads to variants of the inverse problems or inverse operators **W**_***ιv***_ for estimating the specific latent variable ***ι***(*t*), or ***ι***(*f*), or **Σ**_***ιι***_(*f*), as discussed in the next section (Section Bayesian (MAP) inverse operators. Quasilinear (F-invariant) approximation). Estimation of the cross-spectrum **Σ**_***ιι***_(*f*), involves the challenging inverse problem of the matrix equation (route 3) detailed in section (Section Bayesian MAP1 inverse operators. MNE, eLORETA, and LCMV as particular cases).

**Figure 1:**
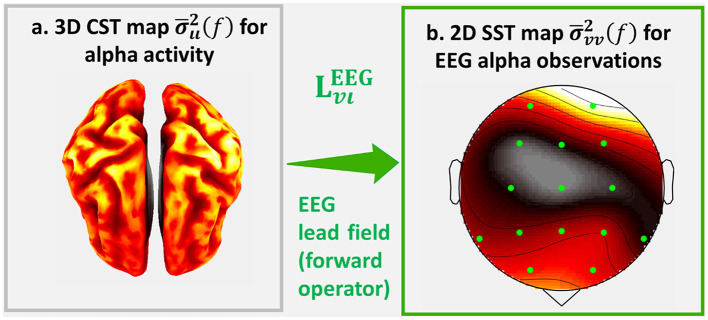
An illustration of **Σ**_***ιι***_(*f*) the source cross-spectrum of human cortical alpha activity and its EEG cross-spectrum **Σ**_***vv***_(*f*). For this illustrations we calculate the sampled estimates ${\bar{\Sigma }}_{\iota \iota }\left(f\right)$ and ${\bar{\Sigma }}_{vv}\left(f\right)$ of which we represent the topographic projections: **(a)** Cortical Spectral Topography (CST) ${\bar{\sigma }}_{\text{u}}^{2}\left(f\right)={\bar{\Sigma }}_{\text{u}}\left(f\right)$ and **(b)** Sensor Spectral Topography (SST) ${\bar{\sigma }}_{vv}^{2}\left(f\right)={\bar{\Sigma }}_{vv}\left(f\right)$.

We currently illustrate the effects of the forward operator with the topographic maps: *Cortical Spectral Topography* (CST), a map of the cortical spectrum ${\bar{\sigma }}_{\iota \iota }^{2}\left(f\right)=diag\left({\bar{\Sigma }}_{\iota \iota }\;\left(f\right)\right)$, and *Sensor Spectral Topography* (SST), a map of the sensor spectrum ${\bar{\sigma }}_{vv}^{2}\left(f\right)=diag\left({\bar{\Sigma }}_{vv}\;\left(f\right)\right)$ (Figure 1). On the left is a hypothetical CST, and on the right is the corresponding SST. The inverse problem consists in estimating the latent CST from observed SST.

### Bayesian (MAP) inverse operators, quasilinear (F-invariant) approximation

**Figure 2:**
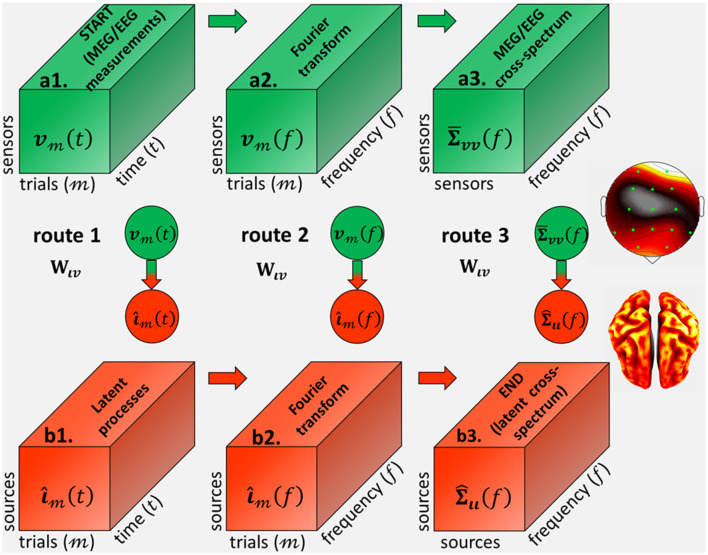
Inverse operators from **(a1)** the MEG/EEG/ECoG sensor signal ***v***_**t**_(*t*), defined as 3D tensor with every trial and time-point observation, to **(b3)** the source cross-spectrum at every frequency **Σ**_***ιι***_(*f*), *via* the different *cross-spectral Electrophysiological Source Imaging* (cESI) routes. Route 1 (**a1**→**b1**→**b2**→**b3**) *via* an inverse operator **W**_***ιv***_ in the time-domain first determines **(b1)** the source processes ***ι***_3_(*t*) and then compute **(b2)** their Fourier transform**ι**_3_(*f*). Route 2 (**a1**→**a2**→**b2**→**b3**) first computes **(a2)** the data Fourier transform ***v***_**m**_(*f*) and then *via* an inverse operator **W**_***ιv***_ in the frequency domain determines **(b2)** their source Fourier transform ***ι***_*m*_(*f*). Route 3 (**a1**→**a2**→**a3**→**b3**) first computes **(a2)** the data Fourier transform ***v***_**3**_(*f*) and then determines **(a3)** the data cross-spectrum **Σ**_***vv***_(*f*). We revindicate Route 3, which is *via* an inverse operator **W**_***ιv***_ in the spectral-domain with a priori probabilities upon the source cross-spectrum.

From the theory of inverse problems (Tarantola, 2005), one may seek inverse solutions for each of the latent variables in the routes corresponding to ***ι***(*t*), or ***ι***(*f*), or **Σ**_***ιι***_(*f*) (Eq. 13) and Figure 2. In each route, the theoretical inverse operator ${\hat{\text{W}}}_{\iota v}$ represents the symbolic projection to the source space and these inverse solutions. In turn, inverse operators are determined (analytically or numerically) by data-driven methods that depend on the routes from the data variable: ***v***(*t*), or ***v***(*f*), or ${\bar{\Sigma }}_{vv}\left(f\right)$, and the forward operator **L**_***vι***_.

(13) $$\begin{array}{l} \begin{array}{c} \left(route\;1\right)\;{\hat{\text{W}}}_{\iota v}\left(v\;\left(t\right)\right)\\ \left(route\;2\right)\;\hat{\iota }\left(f\right)\leftarrow {\hat{\text{W}}}_{\iota v}\left(v\;\left(f\right)\right)\;\\ \left(route\;3\right)\;{\hat{\Sigma }}_{\iota \iota }\left(f\right)\leftarrow {\hat{\text{W}}}_{\iota v}\left({\bar{\Sigma }}_{vv}\;\left(f\right)\right) \end{array} \end{array}$$

Let any of the data variables be represented generically by Y, and likewise, X represent all types of latent variables in Eq. (13). A *maximum a posteriori* (*MAP*) Bayesian inverse operator ${\hat{\text{W}}}_{\mathrm{XY}}$ (Eq. 14) achieves the maximum of *a posteriori* probability q(X|Y). We define the *a posteriori* q(X|Y) as the conditional probability determined from the *likelihood* p(Y|X) and *a priori* p(X). Depending on q(X|Y) an inverse operator ${\hat{\text{W}}}_{\mathrm{XY}}$ can be non-linear or linear, computed numerically or analytically, also intractable or tractable.

(14) $$\begin{array}{l} \begin{array}{c} X\leftarrow {\hat{\text{W}}}_{\mathrm{XY}}\left(Y\right)\\ {\hat{\text{W}}}_{\mathrm{XY}}=argmax_{{\text{W}}_{\mathrm{XY}}}\left(q\left({\text{W}}_{\mathrm{XY}}\left(Y\right)|Y\right)\right)\\ q\left(X|Y\right)\propto p\left(Y|X\right)p\left(X\right) \end{array} \end{array}$$

The essential role of the *a priori* p(X) is to overcome the ill condition of the *likelihood* p(Y|X), in other words, to provide a unique solution to the inverse problem for the source variable X. Here we formulate this *a priori* p(X) as a Gibbs probability density (Eq. 15).

(15) $$\begin{array}{l} p\left(X\right)\propto \exp\left(H\left(X\right)|\alpha \right), \end{array}$$

Where α is the Gibbs temperature parameter. The Gibbs energy function H(X) is commonly defined using the norms of vectors or matrices (Petersen and Pedersen, 2008; Golub and Van Loan, 2013), usually used to reflect empirical criteria about the structure and density of the source variable X defined over the spatial, time, or frequency domains. Assimilating these source qualities requires, in addition, the empirically determined (data-driven) scale parameter α, by some method from the data variable Y.

We emphasize that this Bayesian MAP formalism is particularly helpful in developing the cESI routes and our optimal inverse solution in the following sections (Sections Bayesian MAP1 inverse operators. MNE, eLORETA, and LCMV as particular cases and Data used for the validation). In another context, Eqs. (14, 15) may be completely equivalent to the classical Tikhonov regularization (Tikhonov and Arsenin, 1977), taking *a posteriori* p(X|Y) under the −log transformation. However, the Bayesian formalism is more general than the Tikhonov regularization since the Gibbs energy H(X) (Eq. 15) describes the statistical properties of any physical system (Landau and Lifshitz, 1980).

As stated before, we constrained all routes (Eq. 13) to the class of quasilinear inverse operators **T**_***ιv***_ (Eq. 16) (Baillet et al., 2001; Grech et al., 2008). Moreover, the quasilinear class leads to the type of F-invariant inverse solutions for the properties in Eqs. (10, 11), and particularly the tractable cESI route 3.

(16) $$\begin{array}{l} \begin{array}{c} \left(route\;1\right)\;\hat{\iota }\left(t\right)\leftarrow {\hat{\text{W}}}_{\iota v}\left(v\;\left(t\right)\right)≅{\hat{\text{T}}}_{\iota v}\left(v\;\left(t\right)\right)v\left(t\right)\;\\ \left(route\;2\right)\;\hat{\iota }\left(f\right)\leftarrow {\hat{\text{W}}}_{\iota v}\left(v\;\left(f\right)\right)≅{\hat{\text{T}}}_{\iota v}\left(v\;\left(f\right)\right)v\left(f\right)\;\\ \left(route\;3\right)\;{\hat{\Sigma }}_{\iota \iota }\left(f\right)\leftarrow {\hat{\text{W}}}_{\iota v}\left({\bar{\Sigma }}_{vv}\;\left(f\right)\right)≅{\hat{\text{T}}}_{\iota v}\left({\bar{\Sigma }}_{vv}\;\left(f\right)\right)\\ {\bar{\Sigma }}_{vv}\left(f\right){\hat{\text{T}}}_{v\iota }\left({\bar{\Sigma }}_{vv}\;\left(f\right)\right) \end{array} \end{array}$$

The term “quasilinear” **T**_***ιv***_ is applies to inverse operators defined as non-linear matrix functions of the vector argument ***v***(*t*) or ***v***(*f*), or the matrix argument ${\bar{\Sigma }}_{vv}\left(f\right)$. A matrix function comprises entries **T**_***ιv***_(*g* , *s*) in the *G* × *E* cartesian product of sources *g* = 1⋯*G* and sensors *e* = 1⋯*E*. These entries are non-linear functions of the vector arguments **T**_***ιv***_(*g* , *s* , ***v*** (*t*)), or **T**_***ιv***_(*g* , *s* , ***v*** (*f*)), or the matrix argument ${\text{T}}_{\iota v}\left(g\;,\;s\;,\;{\bar{\Sigma }}_{vv}\;\left(f\right)\;\right)$.

### Bayesian MAP1 inverse operators. MNE, eLORETA, and LCMV as particular cases

Inverse solutions for routes 1 and 2 (Eq. 13) are the traditional *activation Electrophysiological Source Imaging* (aESI). We obtained these inverse solutions as a *first-type MAP* (MAP1) (Eq. 17), which estimates the source variable ***ι***(*t*) in the time domain (Hämäläinen and Ilmoniemi, 1994), or the source variable ***ι***(*f*) in the frequency domain (Salmelin and Hämäläinen, 1995).

(17) $$\begin{array}{l} \begin{array}{c} \left(route\;1\right)\;\hat{\iota }\left(t\right)\leftarrow {\hat{\text{W}}}_{\iota v}\left(v\;\left(t\right)\right)\\ {\hat{\text{W}}}_{\iota v}=argmax_{{\text{W}}_{\iota v}}q\left({\text{W}}_{\iota v}\left(v\;\left(t\right)\right)|v\left(t\right)\right)\\ q\left(\iota \left(t\right)|v\left(t\right)\right)\propto q\left(v\left(t\right)|\iota \left(t\right)\right)p\left(\iota \;\left(t\right)\right)\\ \left(route\;2\right)\;\hat{\iota }\left(f\right)\leftarrow {\hat{\text{W}}}_{\iota v}\left(v\;\left(f\right)\right){\hat{\text{W}}}_{\iota v}=argmax_{{\text{W}}_{\iota v}}q\left({\text{W}}_{\iota v}\left(v\;\left(f\right)\right)|v\left(f\right)\right)\\ q\left(\iota \left(f\right)|v\left(f\right)\right)\propto p\left(v\left(f\right)|\iota \left(f\right)\right)p\left(\iota \;\left(f\right)\right) \end{array} \end{array}$$

The *a posteriori* probabilities require definitions of the *likelihood*, and the *a priori* is given below in Eq. (18). For the data ***v***(*t*) a real-valued Gaussian is commonly assumed with mean **L**_***vι***_***ι***(*t*) and noise covariance **Σ**_**ξξ**_(*t*). This assumption might not be valid for all types of time domain data. In contrast, and in virtue of Brillinger's theorem cited above, data ***v***(*f*) obtained with the discrete Fourier transform will almost certainly have a complex-valued Gaussian *likelihood* with mean **L**_***vι***_***ι***(*f*) and noise cross-spectrum **Σ**_**ξξ**_(*f*) and thus the one we adopted. The *a priori* probabilities in Eq. (18) are placed upon the activation Gibbs energy in the time domain *H*(***ι***(*t*)) or the frequency domain *H*(***ι***(*f*)) (Eq. 18) as expressed by the vector *p-norm* (frequently *p-norm*^*p*^) upon real-valued ***ι***(*t*) or complex-valued ***ι***(*f*) (Riesz, 1910; Rudin, 1970; Dunford and Schwartz, 1988; Bourbaki, 2013).

(18) $$\begin{array}{l} \begin{array}{c} \left(route\;1\right)\;p\left(v\left(t\right)|\iota \left(t\right)\right)=\;N^{\mathbb{R}}\left(v\left(t\right)|{\text{L}}_{v\iota }\iota \left(t\right),\Sigma_{\xi \xi }\left(t\right)\right)\\ p\left(\iota \;\left(t\right)\right)\propto \exp\left(H\left(\iota \;\left(t\right)\right)|\alpha \left(t\right)\right)\\ \left(route\;2\right)\;p\left(v\left(f\right)|\iota \left(f\right)\right)=N^{\mathbb{C}}\left(v\left(f\right)|{\text{L}}_{v\iota }\iota \left(f\right),\Sigma_{\xi \xi }\left(f\right)\right)\\ p\left(\iota \;\left(f\right)\right)\propto \exp\left(H\left(\iota \;\left(f\right)\right)|\alpha \left(f\right)\right) \end{array} \end{array}$$

Henceforth, we ruled out route 1 since it is based on the *ad-hoc* Gaussian assumption for *p*(***v***(*t*)|***ι***(*t*)), which, as mentioned before, is not always tenable. Furthermore, our interest was in the frequency domain, which concentrates our attention on route 2, and which bases the *likelihood* of the Fourier transform *p*(***v***(*f*)|***ι***(*f*)) (Eq. 18) on the complex-valued Gaussian probability (Eq. 10).

Toward cESI, selecting *a priori* *p*(***ι***(*f*)) (Eq. 18) follows the standard rationale that oscillatory brain networks and their activity are characterized by a large-scale and dense (or non-sparse) distribution in conditions of resting-state or task in a block design (Mantini et al., 2007; Brookes et al., 2011b; Hipp et al., 2012; Lopes da Silva, 2013). Such activity is the stochastic and stationary process ***ι***(*t*) that is composited by oscillations in the frequency domain, as described by the Fourier transform ***ι***(*f*) (Engel et al., 2001; Varela et al., 2001; Vidaurre et al., 2018a,b; Tewarie et al., 2019).

A general smooth *a priori* model posits the following Gibbs energy *H*_2, **A**(*f*)_(***ι***(*f*)) (Eq. 19) which, in addition, specifies **A**(*f*) as some positive definite and symmetric, or Hermitian, matrix. Specifying the matrix **A**(*f*) may follow some types of goodness criteria of the inverse solution, and data-driven methods, which lead to the most common cases of quasilinear (F-invariant) inverse operators (Baillet et al., 2001; Hauk, 2004; Friston K. J. et al., 2008; Grech et al., 2008; Marzetti et al., 2008; Henson et al., 2011; Hindriks, 2020).

(19) $$\begin{array}{l} H_{2,\text{A}\left(f\right)}\left(\iota \;\left(f\right)\right)=\iota^{†}\left(f\right){\text{A}}^{-1}\left(f\right)\iota \left(f\right) \end{array}$$

Representing the most common quasilinear cases the following inverse operator ${\hat{\text{T}}}_{\iota v}\left(\text{A}\;\left(f\right)\;,\;\text{B}\;\left(f\right)\right)$, in brief notation ${\hat{\text{T}}}_{\iota v}\left(f\right)$ (Eq. 20), the constrained generalized inverse of the forward operator **L**_***ιv***_ that incorporates the regularization matrices **A**(*f*) and **B**(*f*).

(20) $$\begin{array}{l} \begin{array}{c} \hat{\iota }\left(f\right)\leftarrow {\hat{\text{T}}}_{\iota v}\left(f\right)v\left(f\right)\\ {\hat{\text{T}}}_{\iota v}\left(f\right)={\hat{\Pi }}_{\iota \iota }\left(f\right){\text{L}}_{\iota v}{\text{B}}^{-1}\left(f\right)\;with\;{\hat{\Pi }}_{\iota \iota }\left(f\right)=({\text{L}}_{\iota v}\;{\text{B}}^{-1}\;\left(f\right)\;{\text{L}}_{v\iota }\;\\ +\;\alpha \;\left(f\right)\;{\text{A}}^{-1}\;\left(f\right){)}^{-1}\\ q\left(\iota \left(f\right)|v\left(f\right)\right)\propto N^{\mathbb{C}}\left(\iota \left(f\right)|{\hat{\text{T}}}_{\iota v}\left(f\right)v\left(f\right),{\hat{\Pi }}_{\iota \iota }\left(f\right)\right) \end{array} \end{array}$$

Where ${\hat{\text{T}}}_{\iota v}\left(f\right)$ arises from the route 2 MAP1 (Eq. 17) that incorporates the Gibbs energy *H*_2, **A**(*f*)_(***ι***(*f*)) (Eq. 19), and **B**(*f*) any approximation to the noise cross-spectrum **Σ**_**ξξ**_(*f*) in probabilities (Eq. 18); and *q*(***ι***(*f*)|***v***(*f*)) is the Gaussian *a posteriori* *N*^ℂ^, with mean ${\hat{\text{T}}}_{\iota v}\left(f\right)v\left(f\right)$ and covariance matrix ${\hat{\Pi }}_{\iota \iota }\left(f\right)$, that follows from the conjugated relation between the *likelihood* and *a priori* probabilities in the route 2 MAP1.

The cESI estimator is then ${\bar{\Sigma }}_{\hat{\iota }\hat{\iota }}\left(f\right)$ (Eq. 21) for any set of inverse solution instances $\left({\hat{\iota }}_{m}\;\left(f\right);\;\forall \;m\right)$, or more compactly from an estimate of the data cross-spectrum ${\bar{\Sigma }}_{vv}\left(f\right)$ (Eq. 22).

(21) $$\begin{array}{l} {\bar{\Sigma }}_{\hat{\iota }\hat{\iota }}\left(f\right)=\langle {\hat{\iota }}_{m}\;\left(f\right)\;{\hat{\iota }}_{m}^{†}\;\left(f\right);\;M\rangle \langle {\hat{\iota }}_{m}\;\left(f\right)\;{\hat{\iota }}_{m}^{†}\;\left(f\right);\;M\rangle =\\ \left(\frac{1}{M}\right)\overset{M}{\underset{m=1}{\sum }}{\hat{\iota }}_{m}\left(f\right){\hat{\iota }}_{m}^{†}\left(f\right) \end{array}$$

(22) $$\begin{array}{l} {\bar{\Sigma }}_{\hat{\iota }\hat{\iota }}\left(f\right)={\hat{\text{T}}}_{\iota v}\left(f\right){\bar{\Sigma }}_{vv}\left(f\right){\hat{\text{T}}}_{v\iota }\left(f\right) \end{array}$$

Important examples of cESI that follow route 1 are MNE, eLORETA and LCMV:

- The *Minimum Norm Estimate* (MNE) (Hämäläinen and Ilmoniemi, 1994). The basic smooth model of the source variables, that could either disregard the weight matrix **A**(*f*) = **I** or consider it *ad-hoc* **A**(*f*) = **A**^*ac*^ based on anatomical information.
- The *Linearly Constrained Minimum Variance* (LCMV) (Van Veen et al., 1997). The beamformer method that estimates a diagonal weight matrix **A**(*f*) = *diag*(**a**). This estimation produces an ideal filter (inverse operator) for each source variable, suppressing the interference from the other source variables and performing ideally under focalized distribution around one or a few sources.
- The *Exact Low-Resolution Electromagnetic Tomographic Analysis* (ELORETA) (Pascual-Marqui et al., 2006). A regression method that estimates **A**(*f*) so that the localization of the maximum for the estimated source variables corresponds exactly to the true maximum. This estimation performs ideally under a unimodal and smooth distribution of source variables.

The variants of these techniques are the one optimized for route 2, ESI in the frequency domain: the Spectral eLORETA (seLORETA) (Nolte et al., 2020) and the Spectral LCMV (sLCMV) (Larson-Prior et al., 2013). An additional solution used in this study as a reference is the spectral MNE (sMNE).

## Novel cESI theory leading to the sSSBL approximation

Having described the theory of state-of-the-art cESI methods, we now focus on more sophisticated MAP theory and the sSSBL approximation that allows their practical implementation.

### Bayesian MAP2 inverse operators

It is important to stress that posing *ad-hoc priors* and their hyperparameters is always necessary due to the uncertainties involved, by definition of the MAPs (Eqs. 14, 15) [54], [126]. In route 2, the prior was placed upon the Fourier transform ***ι***(*f*) (Eq. 19). An alternative is to leverage the asymptotic distribution of the Fourier transform (Eq. 10) and place the *a priori* upon **Σ**_***ιι***_(*f*).

This approach brings us to our main contribution, the theoretically promising cESI, which leads to an optimal inverse operator ${\hat{\text{W}}}_{\iota v}$ based on the *second-type MAP* (MAP2) (Eq. 23).

(23) $$\begin{array}{l} \begin{array}{c} \left(r\;o\;u\;t\;e\;3\right)\;{\hat{\Sigma }}_{\iota \iota }\left(f\right)\leftarrow {\hat{\text{W}}}_{\iota v}\left({\bar{\Sigma }}_{vv}\;\left(f\right)\right)\\ {\hat{\text{W}}}_{\iota v}=argmax_{{\text{W}}_{\iota v}}q\left({\text{W}}_{\iota v}\left({\bar{\Sigma }}_{vv}\;\left(f\right)\right)|{\bar{\Sigma }}_{vv}\left(f\right)\right)\\ q\left(\Sigma_{\iota \iota }\left(f\right)|{\bar{\Sigma }}_{vv}\left(f\right)\right)\propto p\left(\Sigma_{vv}\left(f\right)|\Sigma_{\iota \iota }\left(f\right)\right)p\left(\Sigma_{\iota \iota }\;\left(f\right)\right) \end{array} \end{array}$$

This MAP2 above posits the *a priori* probability *p*(**Σ**_***ιι***_ (*f*)) upon the source cross-spectrum **Σ**_***ιι***_(*f*), considered a random hyper-parameter matrix. Incorporating an *a priori* to match the *likelihood* upon the sampled estimator ${\bar{\Sigma }}_{vv}\left(f\right)$ leads to *a posteriori* $q\left(\Sigma_{\iota \iota }\left(f\right)|{\bar{\Sigma }}_{vv}\left(f\right)\right)$. Such a *likelihood* is the Complex Wishart probability density *W*^ℂ^ (Eq. 11) now defined for ${\bar{\Sigma }}_{vv}\left(f\right)$ (Eq. 24) with scale matrix **Σ**_***vv***_(*f*) and *M* degrees of freedom. The specific scale matrix **Σ**_***vv***_(*f*) in this *likelihood* posits the relation to the matrix equation of source cross-spectrum **Σ**_***ιι***_(*f*) (Eq. 12). An *a priori* probability is then upon some cross-spectral Gibbs energy *P*(**Σ**_***ιι***_ (*f*)) defined by norms such as the *vectorized (entry-wise) p-norm* (Ding et al., 2006) and *Schatten p-norm* (Fan, 1951; Schatten, 2013) upon the matrix **Σ**_***ιι***_(*f*).

(24) $$\begin{array}{l} \begin{array}{c} p\left({\bar{\Sigma }}_{vv}\left(f\right)|\Sigma_{\iota \iota }\left(f\right)\right)=W^{\mathbb{C}}\left({\bar{\Sigma }}_{vv}\left(f\right)|{\text{L}}_{v\iota }\Sigma_{\iota \iota }\left(f\right){\text{L}}_{\iota v}+\Sigma_{\xi \xi }\left(f\right),M\right)\\ p\left(\Sigma_{\iota \iota }\;\left(f\right)\right)\propto \exp\left(H\left(\Sigma_{\iota \iota }\;\left(f\right)\right)|\alpha \left(f\right)\right) \end{array} \end{array}$$

Depending on the *likelihood* and *a priori* probability (Eq. 24), the optimal inverse operator ${\hat{\text{W}}}_{\iota v}$ (Eq. 23) is often non-linear and numerically intractable. In this case, which we followed in this article, optimizing ${\hat{\text{W}}}_{\iota v}$ requires Approximated Bayesian Computation (ABC) (Csilléry et al., 2010). The ABC we describe here employed ${\hat{\text{W}}}_{\iota v}^{\left(k\;+\;1\right)}$, a non-convex but numerically tractable successive approximation to ${\hat{\text{W}}}_{\iota v}$. In turn, the tractable ${\hat{\text{W}}}_{\iota v}^{\left(k\;+\;1\right)}$ followed from $q^{\left(k\right)}\left(\Sigma_{\iota \iota }\left(f\right)|{\bar{\Sigma }}_{vv}\left(f\right)\right)$, the non-convex relaxation of the *a posteriori* $q\left(\Sigma_{\iota \iota }\left(f\right)|{\bar{\Sigma }}_{vv}\left(f\right)\right)$ in Expectation-Maximization (EM) iterations. Obtaining the *a posteriori* relaxation is *via* the *Variational Bayes* (VB) treatment (Dempster et al., 1977; Liu and Rubin, 1994; Daunizeau and Friston, 2007; Friston et al., 2007; Nummenmaa et al., 2007; Friston K. J. et al., 2008). Such a VB treatment could target the separable model for $q\left(\Sigma_{\iota \iota }\left(f\right)|{\bar{\Sigma }}_{vv}\left(f\right)\right)$, or the separable *Hierarchical Bayesian* (HB) model for the *a priori* *p*(**Σ**_***ιι***_ (*f*)).

### Bayesian (joint-MAP) inverse operators and cross-spectral norms

Considering the MAP2 (Eq. 23) and the latent cross-spectrum matrix **Σ**_***ιι***_(*f*) constrained within the vector subspace generated by random instances (***ι***_*m*_ (*f*); ∀ *m*), i.e., as if it was defined by its usual estimator $\Sigma_{\iota \iota }\left(f\right)≅{\bar{\Sigma }}_{\iota \iota }\left(f\right)$ (Eq. 7). Hence, the MAP2 is in probability equivalent to the joint-MAP (Hsiao et al., 1998, 2002; Yeredor, 2000; Davis et al., 2001; Auranen et al., 2005; Chen et al., 2011).

We introduce this joint-MAP (Eq. 25) substituting in the general MAP definition (Eqs. 14, 15) the data Y and source variable X by the instances $Y=\left(v_{m}\;\left(f\right);\;\forall \;m\right)$ and $X=\left(\iota_{m}\;\left(f\right);\;\forall \;m\;\right)$.

(25) $$\begin{array}{l} \begin{array}{c} \left(\hat{\iota_{m}\;\left(f\right);\;\forall \;m}\right)\leftarrow {\hat{\text{W}}}_{\iota v}\left(v_{m}\;\left(f\right);\;\forall \;m\right)\\ {\hat{\text{W}}}_{\iota v}\left(v_{m}\;\left(f\right);\;\forall \;m\right)=argmax_{{\text{W}}_{\iota v}}p\left({\text{W}}_{\iota v}\left(v_{m}\;\left(f\right);\;\forall \;m\right)|v_{m}\left(f\right);\forall m\right)\\ p\left(\iota_{m}\left(f\right);\forall m|v_{m}\left(f\right);\forall m\right)\propto p\left(v_{m}\left(f\right);\forall m|\iota_{m}\left(f\right);\forall m\right)p\left(\iota_{m}\;\left(f\right);\;\forall \;m\right) \end{array} \end{array}$$

In our case, we considered the above *p*(***v***_*m*_(*f*);∀*m*|***ι***_*m*_(*f*);∀*m*) the factorizable *joint likelihood* (Eq. 26) whose factors are present throughout the complex-valued Gaussian probability density (*likelihood* for route 2) (Eq. 18). In addition, one may assume **Σ**_**ξξ**_ = *diag*(**β** (*f*)) a univariate (in many cases homogenous) noise model as expressed in terms of the noise spectrum **β**(*f*).

(26) $$\begin{array}{l} \begin{array}{c} p\left(v_{m}\left(f\right);\forall m|\iota_{m}\left(f\right);\forall m\right)=\prod_{m=1}^{M}p\left(v_{m}\left(f\right)|\iota_{m}\left(f\right)\right)\\ p\left(v_{m}\left(f\right)|\iota_{m}\left(f\right)\right)=N^{\mathbb{C}}\left(v_{m}\left(f\right)|{\text{L}}_{v\iota }\iota_{m}\left(f\right),diag\left(\beta \;\left(f\right)\right)\right) \end{array} \end{array}$$

We now introduce the *joint a priori* *p*(***ι***_*m*_ (*f*); ∀ *m*) (Eq. 27) upon the Gibbs energy *H*(***ι***_*m*_ (*f*); ∀ *m*) redefining the previous (Eq. 15) over instances (***ι***_*m*_ (*f*); ∀ *m*). Toward cESI, this function may be read as cross-spectral Gibbs energy $H\left({\bar{\Sigma }}_{\iota \iota }\;\left(f\right)\;\right)$.

(27) $$\begin{array}{l} \begin{array}{c} p\left(\iota_{m}\;\left(f\right);\;\forall \;m\right)\propto \exp\left(H\left(\iota_{m}\;\left(f\right);\;\forall \;m\right)|\alpha \left(f\right)\right)=\\ exp\left(H\left({\bar{\Sigma }}_{\iota \iota }\;\left(f\right)\right)|\alpha \left(f\right)\right) \end{array} \end{array}$$

Our purpose now is to define the type of cross-spectral Gibbs energy that must deal with a severely ill-conditioned and high dimensional cESI setup (*G* ≫ *S*). We shall deal with these problems employing the vector or matrix norms, such as the vector structured *p, q-norm* (Kowalski and Torrésani, 2009a,b; Gramfort et al., 2012, 2013) and the Schatten matrix *p-norm* (Fan, 1951; Schatten, 2013).

To recap, our approach toward cESI is then with the joint-MAP inverse operator ${\hat{\text{W}}}_{\iota v}$ (Eqs. 25–27) [instead of the MAP2 inverse operator (Eqs. 23, 24)]. Toward our target (cESI), we must leverage the class of *joint a priori* probabilities expressed by the cross-spectral Gibbs energy $H\left({\bar{\Sigma }}_{\iota \iota }\;\left(f\right)\right)$, not the more general case of Gibbs energy defined upon the instances *H*(***ι***_*m*_ (*f*); ∀ *m*).

We leverage *H*_1_ (Eq. 28) the structured *p* = *1, q* = *2-norm* (square) that performs sparse regularization of the cross-spectrum topographic projection or spectrum $tr^{\frac{1}{2}}\left({\bar{\Sigma }}_{\iota \iota }\;\left(f\right)\right)$. *H*_1_ is the well-known *Least Absolute Shrinkage and Selection Operator* (LASSO) (Tibshirani, 1996) for vectors ***ι***(*f*) extended or structured over the second dimension index *m* for the set of instances (***ι***_*m*_ (*f*); ∀ *m*). Such a structured norm is a matrix *quasinorm* that does not fulfill the triangle equality over the cross-spectrum ${\bar{\Sigma }}_{\iota \iota }\left(f\;\right)$.

(28) $$\begin{array}{l} H_{1}\left({\bar{\Sigma }}_{\iota \iota }\;\left(f\right)\right)=\sum_{g=1}^{G}{\left(\sum_{m=1}^{M}|\iota_{m}\;\left(g\;,\;f\right){|}^{2}\right)}^{\frac{1}{2}}=tr^{\frac{1}{2}}\left({\bar{\Sigma }}_{\iota \iota }\;\left(f\right)\right) \end{array}$$

In addition, we introduce *H*_2_ (Eq. 29) the structured *p* = *2, q* = *2-norm* (square), Shatten *p* = *1-norm* or nuclear norm $tr\left({\bar{\Sigma }}_{\iota \iota }\;\left(f\right)\right)$, to compensate sparse bias of *H*_1_ (Eq. 28) and regularize eigenvalues for the cross-spectrum ${\bar{\Sigma }}_{\iota \iota }\left(f\right)$. *H*_2_ is the well-known Ridge operator (Hoerl and Kennard, 1970) for a vector ***ι***(*f*) structured over the second dimension index *m* for the set of instances (***ι***_*m*_ (*f*); ∀ *m*).

(29) $$\begin{array}{l} H_{2}\left({\bar{\Sigma }}_{\iota \iota }\;\left(f\right)\right)=\sum_{m=1}^{M}\sum_{g=1}^{G}|\iota_{m}\;\left(g\;,\;f\right){|}^{2}=tr\left({\bar{\Sigma }}_{\iota \iota }\;\left(f\right)\right) \end{array}$$

ESI practice, which is based on either the *sparse* or the smooth models, may be insufficient, though, in many scenarios where brain activity is patch-wise or not wholly sparse, Elastic Net, the linear combination of *sparse/smooth* models, may be ideal (Zou and Hastie, 2005; Vega-Hernández et al., 2008). Such a regularization style is a spatially structured sparsity due to the linear combination of the *sparse* LASSO and *smooth* Ridge operators upon the vector ***ι***(*f*). Hence, for the vector instances (***ι***_*m*_ (*f*); ∀ *m*), the linear combination of the *quasinorm* *H*_1_ (Eq. 28) and the *nuclear norm* *H*_2_ (Eq. 29) leads to the following *joint a priori* probability *p*(***ι***_*m*_ (*f*); ∀ *m*) (Eq. 30).

(30) $$\begin{array}{l} \begin{array}{c} p\left(\iota_{m}\;\left(f\right);\;\forall \;m\right)\propto \exp\left(H_{1}\left({\bar{\Sigma }}_{\iota \iota }\;\left(f\right)\right)|\alpha_{1}\left(f\right)\right)\\ \exp\left(H_{2}\left({\bar{\Sigma }}_{\iota \iota }\;\left(f\right)\right)|\alpha_{2}\left(f\right)\right) \end{array} \end{array}$$

Motivated by ESI practice, our definition of the Elastic Net (Eq. 30) (nuclear quasinorm) diverges from that of previous studies. From these works, the Elastic Net nuclear norm combines the nuclear norm $tr\left({\bar{\Sigma }}_{\iota \iota }\;\left(f\right)\right)$ with the square Schatten *p* = *2-norm* (Frobenius norm) $tr\left({\bar{\Sigma }}_{\iota \iota }^{2}\;\left(f\right)\right)$ (Sun and Zhang, 2012; Chen et al., 2013; Kim et al., 2015; Zhang et al., 2017). This Elastic Net nuclear norm resolves convexity problems of the nuclear norm in the context of matrix completion (Candes and Recht, 2012; Hu et al., 2012) due to a non-convex problem with the sole nuclear norm. Although it was not our purpose to investigate convexity here, this property holds for our Elastic Net nuclear quasinorm, assuming cross-spectrum ${\bar{\Sigma }}_{\iota \iota }\left(f\right)$ upon vector basis (Zou and Hastie, 2005). In addition, we did not consider the vectorized (entry-wise) *p-norm* (Ding et al., 2006). The latter, which might be necessary to regularize off-diagonal entries in some cases (Paz-Linares et al., 2018), failed to ameliorate ill-condition or distortions.

The type of ABC introduced here is known as “gamma-MAP,” the standard VB treatment applied to *a priori* probabilities in the joint-MAP. In turn, the gamma-MAP leads to the quasilinear successive approximations ${\hat{\text{T}}}_{\iota v}^{\left(k\right)}$. Solving the gamma-MAP under cross-spectral Gibbs energy $H\left({\bar{\Sigma }}_{\iota \iota }\;\left(f\right)\right)$ defined by the Elastic Net nuclear quasinorm is the Structured Sparse Bayesian learning algorithm (ssSBL) described in the next section.

### Gamma-MAP and implementation of SSBL

Similarly to the MAP2 (Eq. 23), the joint-MAP inverse operator ${\hat{\text{W}}}_{\iota v}$ (Eq. 25) could be non-linear by nature, depending on the *joint a priori* probability *p*(***ι***_*m*_ (*f*); ∀ *m*). We now introduce the gamma-MAP that achieves the quasilinear joint-MAP version leveraging the idea of mean-field approximation (Kadanoff, 2009). Such a mean-field approximation is the main idea behind the VB treatment applied to the latent variables of neuroimaging data (MacKay, 1998; Roweis and Ghahramani, 1999; Ghahramani and Beal, 2000, 2001; Eyink et al., 2004; Friston et al., 2007; Nummenmaa et al., 2007; Friston K. J. et al., 2008; Babacan et al., 2009).

By assuming the definition for this *joint a priori* *p*(***ι***_*m*_ (*f*); ∀ *m*) upon Gibbs energy *H*(***ι***_*m*_ (*f*); ∀ *m*) (Eq. 27), the mean-field approximation is then to search for the self-consistent energy form $\tilde{H}\left(\iota_{m}\;\left(f\right);\;\forall \;m\right)$ (Eq. 31) as represented by separable (in most cases also indistinguishable) energy terms *H*_*g*_(***ι***_*m*_ (*g* , *f*); ∀ *m*) for each source. A self-consistent energy form *H*_*g*_(***ι***_*m*_ (*g* , *f*); ∀ *m*) also summarizes the interaction field *g* ↔ ∀*g*′, as a property of the original Gibbs energy *H*(***ι***_*m*_ (*f*); ∀ *m*).

(31) $$\begin{array}{l} \begin{array}{c} H\left(\iota_{m}\;\left(f\right);\;\forall \;m\right)≅\tilde{H}\left(\iota_{m}\;\left(f\right);\;\forall \;m\right)=\\ \sum_{g}H_{g}\left(\iota_{m}\;\left(g\;,\;f\right);\;\forall \;m\right) \end{array} \end{array}$$

Obtaining the self-consistent form Eq. (31) was always plausible under the Gibbs energy *H*(***ι***_*m*_ (*f*); ∀ *m*) that fulfills pair-wise separability in identical functions $H\left(\iota_{m}\;\left(g\;,\;f\right)\;,\;\iota_{m}\;\left(g^{{}^{\prime }}\;,\;f\right);\;\forall \;m\right)$ (Eq. 32). Note that this property is known for ensuring the convergence of solutions for the self-consistency field equations in the literature of magnetism (Weiss, 1907, 2001; Le Boudec et al., 2007; Kadanoff, 2009; Zheng et al., 2015). The cross-spectral Gibbs energy $H\left({\bar{\Sigma }}_{\iota \iota }\;\left(f\right)\right)$, as defined by the norms considered here (Elastic nuclear quasinorm (Eq. 30) or others), fulfills such a property.

(32) $$\begin{array}{l} \begin{array}{c} H\left({\bar{\Sigma }}_{\iota \iota }\;\left(f\right)\right)=\\ \sum_{g=1}^{G}\sum_{g^{{}^{\prime }}=1}^{G}H\left(\langle \iota_{m}\;\left(g\;,\;f\right)\;\iota_{m}^{†}\;\left(g^{{}^{\prime }}\;,\;f\right);\;M\rangle \right) \end{array} \end{array}$$

Then, if the *joint a priori* probability *p*(***ι***_*m*_ (*f*); ∀ *m*) (Eq. 27) is written in terms of the separable Gibbs energy $H\left({\bar{\Sigma }}_{\iota \iota }\;\left(f\right)\right)$ (Eq. 32) it turns out the Markov random field property (Eq. 33) that is summarized by $p\left(\iota_{m}\left(g\;,\;f\right);\forall m|\iota_{m}\left(g^{{}^{\prime }}\;,\;f\right);\forall m\right)$ the pair-wise and identical probability factors (Kindermann et al., 1980; Lafferty et al., 2001). Note that such probability factors may approximate but do not strictly represent conditional probabilities.

(33) $$\begin{array}{l} \begin{array}{c} p\left(\iota_{m}\;\left(f\right);\;\forall \;m\right)=\\ \prod_{g=1}^{G}\prod_{g^{{}^{\prime }}=1}^{G}p\left(\iota_{m}\left(g\;,\;f\right);\forall m|\iota_{m}\left(g^{{}^{\prime }}\;,\;f\right);\forall m\right)\\ p\left(\iota_{m}\left(g\;,\;f\right);\forall m|\iota_{m}\left(g^{{}^{\prime }}\;,\;f\right);\forall m\right)\propto \\ \exp\left(P\left(\langle \iota_{m}\;\left(g\;,\;f\right)\;\iota_{m}^{†}\;\left(g^{{}^{\prime }}\;,\;f\right);\;M\rangle \right)|\alpha \left(f\right)\right) \end{array} \end{array}$$

Hence, we target VB—the factorizable (separable) approximation $h^{q}\left(\iota_{m}\;\left(f\right);\;\forall \;m\right)$ (Eq. 34) of the *joint a priori* *p*(***ι***_*m*_ (*f*); ∀ *m*) (Eq. 33). Essential to obtain $h_{g}^{q}\left(\iota_{m}\;\left(g\;,\;f\right);\;\forall \;m\right)$ is the *Hierarchical Bayes* (HB) or *probability mixture* bellow, identical *a priori* *h* and *a posteriori* *q* probabilities that are upon some type of “variational” hyper-parameters **γ**(*g* , *f*). Such an *a posteriori* *q* is also known as the proxy for belief propagation or message passing, denominated iterated conditional mode in the general literature of Markov random fields (Pearl, 1988, 2022; Weiss, 2001; Yedidia et al., 2003; Zheng et al., 2015).

(34) $$\begin{array}{l} \begin{array}{c} p\left(\iota_{m}\;\left(f\right);\;\forall \;m\right)≅h^{q}\left(\iota_{m}\;\left(f\right);\;\forall \;m\right)\propto \prod_{g=1}^{G}h_{g}^{q}\left(\iota_{m}\;\left(g\;,\;f\right);\;\forall \;m\right)\\ h_{g}^{q}\left(\iota_{m}\;\left(g\;,\;f\right);\;\forall \;m\right)=\\ \int h\left(\iota_{m}\left(g\;,\;f\right);\forall m|\gamma \left(g\;,\;f\right)\right)q\left(\gamma \left(g\;,\;f\right)|\iota_{m}\left(f\right);\forall m\right)d\gamma \left(g\;,\;f\right) \end{array} \end{array}$$

Where the hyper-parameters **γ**(*g* , *f*) condense field interactions *H*(〈***ι*** (*g* , *f*) ***ι***^†^ (*g*′ , *f*); *M*〉) (Eq. 33) by the definition of an *a posteriori* probability *q* upon (***ι***_*m*_ (*f*); ∀ *m*).

After specifying an *a priori* *h*, the VB treatment is applied to search for estimators of the *a posteriori* $\hat{q}$ (Eq. 35). That is to minimize the Kullback-Leibler divergence $D_{KL}\left(p\;,\;h^{q}\right)$ of the approximation *h*^*q*^regarding to the *joint a priori* *p* (Eq. 34).

(35) $$\begin{array}{l} \begin{array}{c} \hat{q}=argmin_{q}D_{KL}\left(p\;||\;h^{q}\right)\\ D_{KL}\left(p\;||\;h^{q}\right)=\\ \int p\left(\iota_{m}\;\left(f\right);\;\forall \;m\right)\log\left(p\;\left(\iota_{m}\;\left(f\right);\;\forall \;m\right)/h^{q}\left(\iota_{m}\;\left(f\right);\;\forall \;m\right)\right)\\ d\left(\iota_{m}\;\left(f\right);\;\forall \;m\right) \end{array} \end{array}$$

Minimizing the $D_{KL}\left(p\;,\;h^{q}\right)$, although theoretically achievable, turned out to be a difficult variational calculus problem for non-parametric HB (mixture) models (Blei and Jordan, 2006; Bryant and Sudderth, 2012; Gershman and Blei, 2012; Gershman et al., 2012; Nguyen and Bonilla, 2013; Duvenaud et al., 2016). Thus, the *D*_*KL*_ approach is common with parametric solutions constraining models (*a priori* *h* and *a posteriori* *q*) to the exponential family of probabilities (Andersen, 1970; Casella and Berger, 2021).

A simplifying assumption that could bypass the Kullback-Leibler divergence $D_{KL}\left(p\;,\;h^{q}\right)$ (Eq. 35) is to regard the *a priori* *h* and *a posteriori* *q* (34) within a family of conjugate HB models of the *likelihood*. Furthermore, we considered *a priori* *h* factorizable over the set of instances since the pair-wise interactions are additive by definition of 〈***ι*** (*g* , *f*) ***ι***^†^ (*g*′ , *f*); *M*〉 the cross-spectrum entries in Eq. (7).

Henceforth, a complex-valued Gaussian probability *N*^ℂ^ defines the *a priori* *h*(***ι***_*m*_(*f*)|**γ**(*f*)), and a probability in the Gamma family Γ defines the *a posteriori* *q*(**γ**(*f*)|***ι***_*m*_(*f*) ; ∀*m*) which we incorporate into the following HB model (Eq. 36).

(36) $$\begin{array}{l} \begin{array}{c} h^{q}\left(\iota_{m}\;\left(f\right);\;\forall \;m\right)\propto \int \left(\prod_{m=1}^{M}h\left(\iota_{m}\left(f\right)|\gamma \left(f\right)\right)\right)q\left(\gamma \left(f\right)|\iota_{m}\left(f\right);\forall m\right)\\ d\gamma \left(f\right)\\ h\left(\iota_{m}\left(f\right)|\gamma \left(f\right)\right)=N^{\mathbb{C}}\left(\iota_{m}\left(f\right)|0,diag\left(\gamma \;\left(f\right)\right)\;\right)\\ q\left(\gamma \left(f\right)|\iota_{m}\left(f\right);\forall m\right)=\prod_{g=1}^{G}\Gamma \left(\gamma \left(g\;,\;f\right)|\delta \left(g\;,\;\iota_{m}\;\left(f\right);\;\forall \;m\right)\right) \end{array} \end{array}$$

The previous HB model is the *Generalized Gaussian Scale Mixture Model* (GGSMM) prescribed by the *Andrews and Mallows lemma* (Andrews, 1974), where the particular and “Gamma” probability Γ depends on *joint a priori* to be approximated *via* $D_{KL}\left(p\;,\;h^{q}\right)$ (Eq. 35) and falls within the class of scale mixture Gamma models (McLachlan and Basford, 1988; Lindsay, 1995; Böhning and Seidel, 2003; Hancock and Samuelsen, 2007).

In such a mixture model, the hyper-parameter vector **γ**(*f*) may then be interpreted as the “variational spectrum,” which specifies a Complex Gaussian *a priori* probability *N*^ℂ^ (Eq. 36). The *a posteriori* for the variational spectrum entries **γ**(*g* , *f*) is a form of probability that belongs to the Gamma Γ family, with a parameterization **δ**(*g* , ***ι***_*m*_ (*f*); ∀ *m*) that condenses field interactions *H*(〈***ι*** (*g* , *f*) ***ι***^†^ (*g*′ , *f*); *M*〉) (Eq. 32).

The joint-MAP (Eq. 25) can be approximated sequentially within iterations for the set $\left(\iota_{m}^{\left(k\right)}\;\left(f\right);\;\forall \;m\right)$, or directly the cross-spectrum ${\hat{\Sigma }}_{\iota \iota }^{\left(k\right)}\left(f\right)$, due to the quasilinear inverse operator ${\text{T}}_{\iota v}^{\left(k\right)}$ (37). Quasilinear ${\text{T}}_{\iota v}^{\left(k\right)}$ was then equivalent to iterated MAP1s for the Fourier transform (Eq. 20), positing complex-valued Gaussian *likelihood* *p*(***v***_*m*_(*f*)|***ι***_*m*_(*f*)) (Eq. 26) and *a priori* *h*(***ι***_*m*_(*f*)|**γ**(*f*)) (36).

(37) $$\begin{array}{l} \begin{array}{c} \hat{\left(\iota_{m}^{\left(k\right)}\;\left(f\right);\;\forall \;m\right)}\leftarrow {\text{T}}_{\iota v}^{\left(k\right)}\left(f\right)\left(v_{m}\;\left(f\right);\;\forall \;m\right)\\ {\hat{\bar{\Sigma }}}_{\iota \iota }^{\left(k\right)}\left(f\right)={\text{T}}_{\iota v}^{\left(k\right)}\left(f\right){\bar{\Sigma }}_{vv}\left(f\right){\text{T}}_{v\iota }^{\left(k\right)}\left(f\right) \end{array} \end{array}$$

These iterated MAP1s are for an equivalent *a posteriori* $q^{\left(k\right)}\left(\iota_{m}\left(f\right)|v_{m}\left(f\right)\right)$ (Eq. 38), specified by the mean ${\text{T}}_{\iota v}^{\left(k\right)}\left(f\right)v\left(f\right)$ and the covariance $\Pi_{\iota \iota }^{\left(k\right)}\left(f\right)$, which are functions of the variational spectrum *diag*(**γ**^(*k*)^ (*f*)) and the noise spectrum *diag*(**β**^(*k*)^ (*f*)). These spectrums specified the type of univariate approximations for the source cross-spectrum **Σ**_***ιι***_ , and the noise cross-spectrum **Σ**_**ξξ**_ in the quasilinear MAP1 of the Fourier transform (Eq. 20). Henceforth, we place the emphasis in the variational spectrum iterations **γ**^(*k*)^(*f*) and defer this noise spectrum **β**^(*k*)^(*f*) which is not essential for our main exposition.

(38) $$\begin{array}{l} \begin{array}{c} q^{\left(k\right)}\left(\iota_{m}\left(f\right)|v_{m}\left(f\right)\right)=N^{\mathbb{C}}\left(\iota_{m}\left(f\right)|{\text{T}}_{\iota v}^{\left(k\right)}\left(f\right)v_{m}\left(f\right),\Pi_{\iota \iota }^{\left(k\right)}\left(f\right)\;\right)\\ {\text{T}}_{\iota v}^{\left(k\right)}\left(f\right)={\hat{\Pi }}_{\iota \iota }^{\left(k\right)}\left(f\right){\text{L}}_{\iota v}diag^{-1}\left(\;\beta^{\left(k\right)}\;\left(f\right)\right)\\ \Pi_{\iota \iota }^{\left(k\right)}\left(f\right)={\left({\text{L}}_{\iota v}\;d\;i\;a\;g^{-1}\;\left(\;\beta^{\left(k\right)}\;\left(f\right)\right)\;{\text{L}}_{v\iota }\;+\;d\;i\;a\;g^{-1}\;\left(\gamma^{\left(k\right)}\;\left(f\right)\right)\right)}^{-1} \end{array} \end{array}$$

Targeting the variational vector **γ**(*f*) was under the marginal or hyper-parametrized *likelihood* for data *p*(***v***_*m*_(*f*)|**γ**(*f*)) (Eq. 39). This *likelihood* was due to integration (expectation) of *p*(***v***_*m*_(*f*)|***ι***_*m*_(*f*)) under the *a priori* probability for parameters *h*(***ι***_*m*_(*f*)|**γ**(*f*)), which is upon the variational hyper-parameters (spectrum) **γ**(*f*) (Eq. 30) (MacKay, 1999).

(39) $$\begin{array}{l} \begin{array}{c} p\left(v_{m}\left(f\right)|\gamma \left(f\right)\right)=\int p\left(v_{m}\left(f\right)|\iota_{m}\left(f\right)\right)h\left(\iota_{m}\left(f\right)|\gamma \left(f\right)\right)d\iota_{m}\left(f\right)\\ p\left(v_{m}\left(f\right)|\iota_{m}\left(f\right)\right)=N^{\mathbb{C}}\left(v_{m}\left(f\right)|{\text{L}}_{v\iota }\iota_{m}\left(f\right),diag\left(\beta \;\left(f\right)\right)\right)\\ h\left(\iota_{m}\left(f\right)|\gamma \left(f\right)\right)=N^{\mathbb{C}}\left(\iota_{m}\left(f\right)|0,diag\left(\gamma \;\left(f\right)\right)\;\right) \end{array} \end{array}$$

However, the actual marginal *likelihood* *p*(***v***_*m*_(*f*)|**γ**(*f*)) (Eq. 39) was intractable, with approximations *via* the expectation of the *log joint* probability *p*(***v***_*m*_(*f*), ***ι***_*m*_(*f*)|**γ**(*f*)) (Dempster et al., 1977; Liu and Rubin, 1994) under the iterated *a posteriori* $q^{\left(k\right)}\left(\iota_{m}\left(f\right)|v_{m}\left(f\right)\right)$ (Eq. 40). Then an approximated marginal *likelihood* $L\left(v_{m}\left(f\right)|\gamma^{\left(k\right)}\left(f\right),\gamma \left(f\right)\right)$ depended on the variational hyper-parameters (spectrum) **γ**^(*k*)^(*f*)in the current iteration.

(40) $$\begin{array}{l} \begin{array}{c} \log\left(p\;\left(v_{m}\left(f\right)|\gamma \left(f\right)\right)\right)≅log\left(L\;\left(v_{m}\left(f\right)|\gamma^{\left(k\right)}\left(f\right),\gamma \left(f\right)\right)\right)=\\ \int q^{\left(k\right)}\left(\iota_{m}\left(f\right)|v_{m}\left(f\right)\right)\log\left(p\;\left(v_{m}\left(f\right)|{\text{L}}_{v\iota }\iota_{m}\left(f\right)\right)\;h\;\left(\iota_{m}\left(f\right)|\gamma \left(f\right)\right)\right) \end{array}\\ d\iota_{m}\left(f\right) \end{array}$$

An inverse operator **W**_***ιv***_ of the variational spectrum **γ**(*f*) is theoretically equivalent to the previous joint-MAP (Eq. 25), which is inverse operator of the samples (***ι***_*m*_ (*f*); ∀ *m*). This is known from the literature as “gamma-MAP” (Hsiao et al., 1998, 2002; Wipf and Nagarajan, 2009). Within the loop (Eq. 41) such an inverse operator ${\text{W}}_{\iota v}^{\left(k\right)}$ was the tractable successive approximations, with the iterated *a posteriori* $q\left(\gamma \left(f\right)|\iota_{m}^{\left(k\right)}\left(f\right);\forall m\right)$ and *likelihood* $L\left(v_{m}\left(f\right)|\gamma^{\left(k\right)}\left(f\right),\gamma \left(f\right)\;\right)$.

(41) $$\begin{array}{l} \begin{array}{c} \gamma^{\left(k\;+\;1\right)}\left(f\right)\leftarrow {\hat{\text{W}}}_{\iota v}^{\left(k\right)}\left(v_{m}\;\left(f\right);\;\forall \;m\right)\\ {\hat{\text{W}}}_{\iota v}\left(v_{m}\;\left(f\right);\;\forall \;m\right)=\\ argmax_{{\text{W}}_{\iota v}}p^{\left(k\right)}\left({\text{W}}_{\iota v}\left(v_{m}\;\left(f\right);\;\forall \;m\right)|v_{m}\left(f\right);\forall m\right)\\ p^{\left(k\right)}\left(\gamma \left(f\right)|v_{m}\left(f\right);\forall m\right)\propto \left(\prod_{m=1}^{M}L\left(v_{m}\left(f\right)|\gamma^{\left(k\right)}\left(f\right),\gamma \left(f\right)\right)\right)\\ q\left(\gamma \left(f\right)|\iota_{m}^{\left(k\right)}\left(f\right);\forall m\right) \end{array} \end{array}$$

Here we implemented the Bayesian learning algorithm that effectuates the gamma-MAP **γ**^(*k* + 1)^(*f*) (41) and computes the quasilinear ${\hat{\text{T}}}_{\iota v}^{\left(k\right)}$ (Eq. 38), for a *joint a priori* based on the Elastic Net nuclear quasinorm (Eq. 30). For this *joint a priori*, the solution to the HB (mixture) model (Eq. 36) is exact, following an extension of the *Andrews and Mallows* lemma to the structured sparsity norms which are here upon the spectrum ${\bar{\sigma }}_{\iota \iota }^{2}\left(f\;\right)$.

Henceforth, we refer to this algorithm as Spectral Structured Sparse Bayesian Learning (ssSBL). The full derivation of the gamma-MAP and the ssSBL algorithm are developed in Supplementary material (Section Standard cESI theory).

## Comparison of the distortions produced by seLORETA, sLCMV, and sSSBL

### Data used for the validation

We used two different sets of EEG data to calculate estimated CST, each with their corresponding gold standard CST:

1. Realistically simulated low-density EEG obtained considering as sources a CST obtained from a MEG recording with a very high sensor density (simulated-EEGvsMEG). The gold standard here is the sMNE CST of the MEG recording due to the well-known advantages of MEG over EEG and the very high sensor density.
2. Real low-density macaque EEG (EEGvsECoG). The gold standard here is the sMNE CST of the simultaneously recorded EcoG.

#### Simulated-EEG vs. MEG

**Figure 3:**
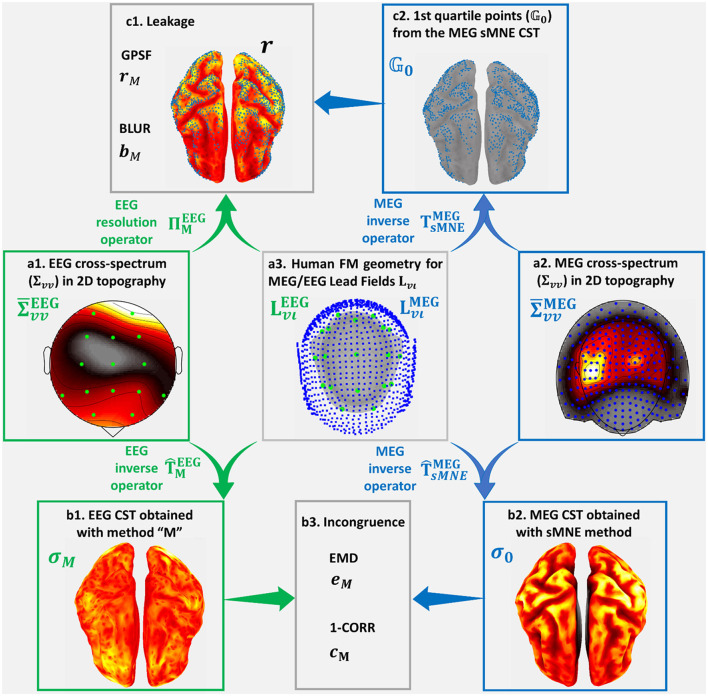
Simulated-EEG vs. MEG experiment that illustrates our validation strategy. We show distortion measures of the estimated Cortical Spectral Topographies (CST) resting-state MEG alpha activity. The same strategy is followed for all spectral bands. It is also applied to the study of distortion based on the EEG vs. ECoG experiment. We start from the cross-spectrum data shown in hot colormaps of 2D space topographies **(a1)** $\sum_{vv}^{EEG}$ for EEG sensors (green dots) and **(a2)** $\sum_{vv}^{MEG}(\sum_{vv}^{ECoG}$) for MEG (ECoG) sensors (blue dots). Using the corresponding cross-spectrum data and Lead Fields **(a3)** ${\text{L}}_{v\iota }^{EEG}$ and ${\text{L}}_{v\iota }^{MEG}$ (${\text{L}}_{v\iota }^{ECoG}$), for EEG sensors (green dots) and MEG (ECoG) sensors (blue dots), upon human (macaque) cortex, head layers geometries, we obtain the inverse operators for EEG ${\overset{\wedge }{T}}_{M}^{EEG}$, based on the tested method “M,” and MEG (ECoG) ${\overset{\wedge }{T}}_{sMNE}^{MEG}\left({\overset{\wedge }{T}}_{sMNE}^{ECoG}\right)$, based on the reference method sMNE. Employing these inverse operators, we obtain estimators for **(b1)** $\sigma_{M}^{EEG}$ the EEG tested CST given by method “M” and **(b2)** **σ**_0_ the MEG (ECoG) sMNE reference CST. Incongruence between **(b1)** the EEG tested CST and **(b2)** the MEG (ECoG) reference CST is measured through **(b3)** **e**_*M*_ the Earth Movers' Distance (EMD) and **c**_*M*_ the correlation distance (1-CORR). Using **(a1)** the EEG cross-spectrum data and **(b3)** Lead Field for EEG, we obtain the resolution operator **R**_*M*_. Leakage in **(b1)** the EEG tested CST, which is based in **(c2)** the 1st quartile point of **(b2)** the MEG (ECoG) reference CST, is measured through **(c1)** **r**_*M*_, the Generalized Point Spread Function (GPSF) and **b**_*M*_ the Blurring for the GPSF.

Figure 3 was based on a high-quality resting state MEG recording for subject 175,237 from the HCP database. The MEG signal selected for this purpose (Figure 3a2) was the 246-channel preprocessed data file. The electrical and magnetic lead fields were calculated with the subject's cortical and head geometry. With the magnetic lead field, Spectral MNE (sMNE) was used to calculate the MEG sources, which were taken as sources for the EEG. These sources were passed through the electrical Lead Field (Figure 3a3) to simulate a low-density 19-channel EEG recording (Figure 3a1). The simulation design is standard, essentially the same as those based on more straightforward configurations, where dipoles or patches are taken as the “ground truth” (Haufe and Ewald, 2019). Here, we used a much more realistic set of sources, determined from the MEG. Code availability: https://github.com/CCC-members/MEGvsSimulated-EEG.

#### EEG vs. ECoG

**Figure 4:**
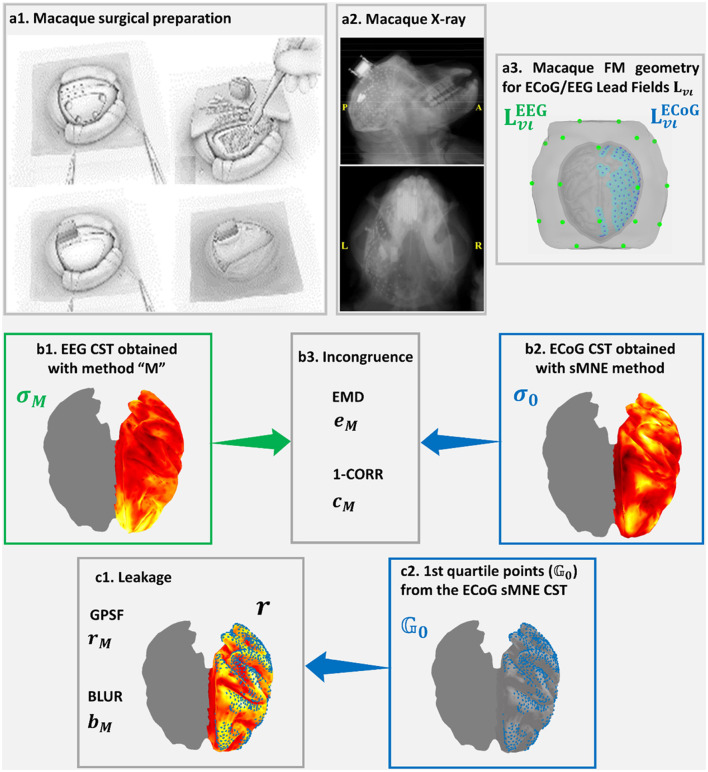
Confirmation of Cortical Spectral Topographies (CST) based in EEG recorded simultaneously with **(a1)** ECoG implanted in the macaque. An X-ray image shows **(a2)** the high-density ECoG array placed onto the macaque cortical surface. ECoG recordings and **(a3)** their Lead Fields provide a more fine-grained reference for confirming CST estimators and measures of distortions for the EEG. The validation here includes elements analogous to the MEGvsSimulated-EEG experiment of Figure 3 **(a3, b1–b3, c1, c2)**. Incongruence between **(b1)** **σ**_*M*_ the EEG tested CST and **(b2)** **σ**_*o*_ the ECoG reference CST is measured through **(b3)** $e_{M}^{ECoG\to EEG}$ the Earth Movers' Distance (EMD) and $C_{M}^{ECoG\to EEG}$ the correlation distance (1-CORR). Leakage in **(b1)** **σ**_*M*_ the EEG tested CST, which is based in **(c2)** on the first quartile point of **(b2)** **σ**_*o*_ the ECoG reference CST is measured through **(c1)** r_*M*_, the Generalized Point Spread Function (GPSF) and b_*M*_ the Blurring for the GPSF. Elements **(a1, a2)** of this figure, are freely available in http://www.www.neurotycho.org/.

Figure 4 was based on high-density macaque ECoG recordings acquired in 128 sensors, concurrently with a low-density EEG acquired at 19 sensors simultaneously with the ECoG in the resting state (Nagasaka et al., 2011). This macaque preparation allowed the realistic measurement of distortions in resting-state connectivity estimated from low-density EEG using different aESI solutions (Wang et al., 2019). A sensor array placed surgically on the left macaque's cortical surface (Figure 4a1) allowed dense recordings of ECoG (Figure 4a2). The lead fields for EEG and ECoG (Figure 4a3) were obtained using the macaque's cortical and head geometry. The ECoG lead field was used to generate a reference or ground truth CST using sMNE. It was unnecessary to simulate data since the concurrent low-density EEG was available. Code availability: https://github.com/CCC-members/ECoGvsEEG.

#### Validation rationale

We evaluated the low-density EEG recordings using ssSBL, sELORETA, and sLCMV to produce the CSTs **σ**_*sSSBL*_, **σ**_*sELOR*_, and **σ**_*sLCMV*_, respectively. We denote these CST generically as **σ**_*M*_, with *M*specifying the method. These calculations were carried out for the previously mentioned experiments: simulated EEG vs. MEG and EEG vs. ECOG. These CSTs were compared with reference CSTs (**σ**_0_) considered the “gold standards.” This reference was varied according to the type of measurement and will be detailed in the context below. Finally, each method's distortion of its reference was assessed using the measures described next.

### Measures of distortion

Measures of distortion to compare **σ**_*M*_ with **σ**_0_ fall into two groups, leakage and incongruence indices that we illustrate in the Simulated-EEG vs. MEG experiment (Figure 3). The measures of the macaque EEG vs. ECoG experiment in figure elements (Figures 4b1–b3, c1, c2) are analogous to those of the Simulated-EEG vs. MEG experiment.

*Leakage* (Palva et al., 2018; Van de Steen et al., 2019), or spread, is quantified using the Generalized Point Spread Function and a blurring measure. These are based on the concept of the resolution operator **R**_*M*_ = **T**_**ι*****v***, *M*_**L**_***vι***_. Here **T**_**ι*****v***, *M*_ is the inverse operator for the method *M*, and **L**_***vι***_ the lead field. Consider a point source **u**_*o*_ indexed by *g*_0_, any column **R**_*M*_(:, *g*_0_) is then the voltages ***v***_0_ produced by this source.

- The Generalized Point Spread Function (Grova et al., 2006b; Haufe et al., 2008) (GPSF) **r**_*M*_ in Figure 3c1, depicted with a hot colormap, represents the leakage (spread) of the low-density EEG CST estimators for a given set of cortical points. These cortical points, shown as blue dots (Figure 3c2), are selected as the most active 25% of the reference **σ**_0_ and are the set *G*_0_. The GPSF, for any point *g* ∈ *G* in the set of cortical points, is then:

(42) $$\begin{array}{l} {\text{r}}_{M}\left(g\right)=\sqrt{\frac{1}{\left|{𝔾}_{0}\right|}\sum_{g_{0}\in {𝔾}_{0}}|{\text{R}}_{M}\;\left(g\;,\;g_{0}\right){|}^{2}} \end{array}$$

Where |𝔾_0_| denotes the number of elements in that set.

- The blurring measure for images (BLUR) *b*_*M*_ is defined as the Spatial Dispersion (SD) of the GPSF (Grova et al., 2006a; Haufe et al., 2008). It is worth clarifying that for a perfect cESI solution, with zero *b*_*M*_ = 0 in Figure 3c1, there would be an exact coincidence between the GPSF **r**_*M*_ non-zero values in the colormap and the blue dots in Figure 3c2. Formally, *b*_*M*_ is defined as:

(43) $$\begin{array}{l} b_{M}=\sqrt{\frac{1}{\left|{𝔾}_{0}\right|}\sum_{g_{0}\in {𝔾}_{0}}\vartheta_{M}^{2}\left(g_{0}\right)}, \end{array}$$

Where $\vartheta_{M}^{2}\left(g_{0}\right)$ is the spatial dispersion around the reference point *g*_0_ and


(44) $$\begin{array}{l} \vartheta_{M}^{2}\left(g_{0}\right)=\frac{1}{\left|𝔾\right|}\sum_{g\in 𝔾}|{\text{R}}_{M}\;\left(g\;,\;g_{0}\right)|d_{gg_{0}}^{2}, \end{array}$$

Where $d_{gg_{0}}^{2}$ is the square of the geodesic distance between those points.

*Incongruence* (Wang et al., 2019) quantifies the global level of distortion (leakage and localization error):

1. The Earth Movers' Distance (EMD) *e*_*M*_ in Figure 3b3, which measures the effort to deform the CST spatial density determined from EEG (Figure 3b1) into the reference (Figure 3b2) (Grova et al., 2006b; Haufe et al., 2008; Paz-Linares et al., 2017).
2. The correlation distance (1-CORR) *c*_*M*_ which measures deformations from the expected collinearity between pairs of spatially distributed CSTs.

These incongruence measures (EMD) *e*_*M*_ correlation distance (1-CORR) *c*_*M*_ combine sensitivity to leakage and localization error.

## Results

### Simulated-EEG vs. MEG inverse solutions

**Figure 5:**
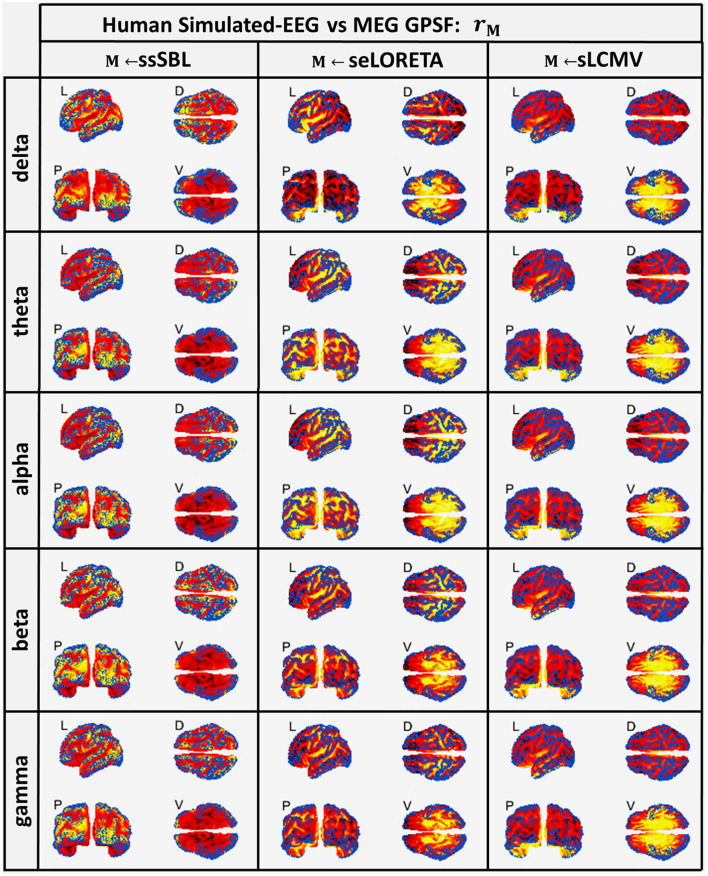
In hot colormaps, measurements of “Leakage” in the EEG-based Cortical Spectral Topographies (CST) were obtained with all tested methods “M” (ssSBL, seLORETA, and sLCMV). Leakage is measured by employing r_*M*_, the Generalized Point Spread Function (GPSF) regarding to the MEG sMNE reference CST, shown in the cortical views Left (L), Dorsal (D), Posterior (P), and Ventral (V) and for five characteristic spectral bands (delta, theta, alpha, beta, and gamma). Leakage distortions are proportional to mismatch and spread in the GPSF values (hot colormaps) regarding reference points (blue dots). Calculations of the GPSF follow the procedure described in Figure 3 for the MEGvsSimulated-EEG experiment.

Both sELORETA and sLCMV CST estimators were seriously affected by leakage (Figure 5), judging by the mismatch between their estimated GPSF **r**_*sELOR*_ and **r**_*sLCMV*_ compared to the reference points (blue dots). They were centered at incorrect sites (opposite the blue dots) and with a much larger spread. For ssSBL, the set of local maxima for **r**_*ssSBL*_ was correctly centered around reference points and did not extend significantly beyond these. In other words, as can be appreciated qualitatively, **r**_*ssSBL*_ minimized leakage compared to sELORETA and LCMV.

Initially, these results differed from those reported by other authors for **r**_*sELOR*_ and **r**_*sLCMV*_ (Haufe and Ewald, 2019). The explanation may be due to their using idealized EEG simulations (point generators or discrete patches) that generate fewer distortions, thus reducing the apparent differences in the performance of different aESI methods. We verified this in recent aESI studies (Paz-Linares et al., 2017), concluding that simple SSBL (not the cESI ssSBL implemented in this paper) outperforms eLORETA and other methods only by a narrow margin.

An aESI solution computed with LCMV was usually sparser than an ELORETA solution, which was expected due to the thresholding strategy implemented in the original LCMV (not sLCMV) (Van Veen et al., 1997). However, this did not lead to any improvement in terms of the leakage observed in the GPSFs **r**_*sELOR*_ and **r**_*sLCMV*_. The “zero localization error” of eLORETA has been claimed to be the unique advantage in its favor (Pascual-Marqui et al., 2006). However, this property has been theoretically demonstrated only for the peak (maximum) activity and not for the numerous local maxima of secondary activations common in real-life scenarios. Therefore, unsurprisingly, eLORETA can produce much better results in idealized simulations that use activity modeled as a focalized concentrated unimodal distribution (e.g., Gaussian) or only a few simple point sources (Kobayashi et al., 2003; Grova et al., 2008; Schoffelen and Gross, 2009; Haufe et al., 2013; Burle et al., 2015; Colclough et al., 2015; Bradley et al., 2016; Mahjoory et al., 2017; Stokes and Purdon, 2017; Palva et al., 2018; Haufe and Ewald, 2019; Marinazzo et al., 2019).

A striking observation was that despite **σ**_0_ being highly frequency dependent, the GPSF pattern was **r**_*seLORETA*_ and **r**_*sLCMV*_ was relatively invariant for all frequencies across the spectrum. This invariance suggests that the pattern was mainly due to Lead Field properties rather than physiologically (Lopes da Silva et al., 1974; Niedermeyer and da Silva, 2005; Lopes da Silva, 2013). In contrast, the GPSF **r**_*ssSBL*_ pattern was closely tied to the physiological fluctuations across frequencies: from activity in the slow delta band to the faster alpha band. These fluctuations were inherited from the reference MEG data.

**Figure 6:**
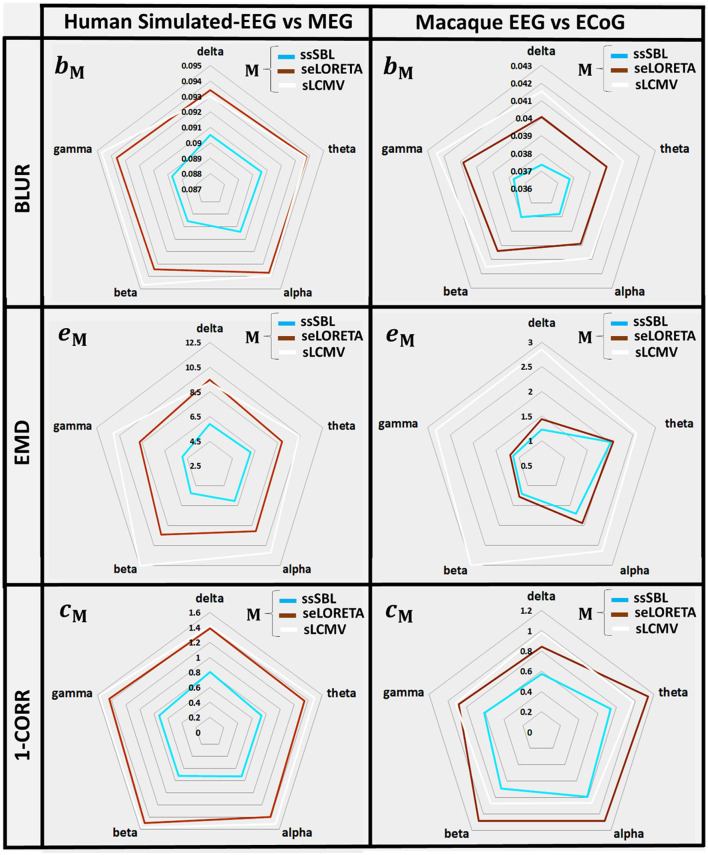
In radar graphs, measurements of “Leakage” and “Incongruence” in the Cortical Spectral Topographies (CST) were obtained with all tested methods “M” (ssSBL, seLORETA, and sLCMV) from the EEG. Leakage, regarding “Y,” the MEG or ECoG sMNE reference CST, is measured employing B_*M*_, the Blurring (BLUR), and Incongruence, employing e_*M*_ the Earth Movers' Distance (EMD), and C_*M*_ the Correlation Distance (1-CORR), shown for five characteristic spectral bands (delta, theta, alpha, beta, and gamma). Distortions based in the BLUR, EMD, and 1-CORR are proportional to their values in the radar graph. Calculations of the BLUR, EMD, and 1-CORR follow the procedure described in Figures 3, 4, for the MEGvsSimulated-EEG and ECoGvsEEG experiments.

The quantitative analysis of the leakage measure *b*_*M*_ (BLUR) (Figure 6, left-column) confirms our qualitative impressions based on the GPSF **r**_*M*_. The radar graphs showed a decrease in leakage of ssSBL compared to sELORETA and sLCMV (*b*_*sELOR*_ > *b*_*ssSBL*_, *b*_*sLCMV*_ > *b*_*ssSBL*_). This improvement was valid for all spectral bands (delta, theta, alpha, gamma 1, and gamma 2).

EMD values *e*_*M*_ are intuitively the amount of work to deform the EEG-based CST estimator to the reference CST estimator (**σ**_*M*_ → **σ**_0_). They were one to two orders larger for sELORETA and sLCMV than SSBL (*e*_*sELOR*_ >> *e*_*ssSBL*_, and *e*_*sLCMV*_ >> *e*_*ssSBL*_).

**Figure 7:**
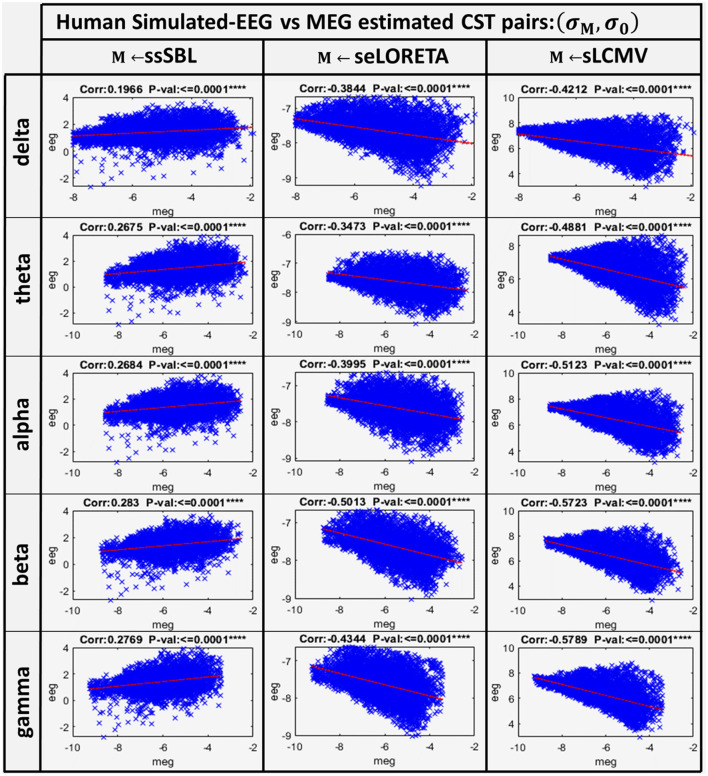
Linear model and correlations for the Cortical Spectral Topographies (CST) obtained from the MEGvsSimulated-EEG experiment in the human. These were adjusted from the CST data pairs (**σ**_*M*_, **σ**_0_) for all tested methods “M” (ssSBL, seLORETA, and sLCMV), and in five characteristic spectral bands (delta, theta, alpha, beta, and gamma).

A linear model adjusted to every source of the EEG and MEG CSTs estimated data pairs shows a clear linear tendency (Figure 7), with correlation distances larger for sELORETA and sLCMV. Some correlations are even negative correlations. By contrast, *c*_*sELOR*_ was in the range of around 0.7, for all frequency bands. This behavior was congruent with that observed in the colormaps for GPSF (**r**_*sELOR*_ and **r**_*sLCMV*_) (Figure 5), where the maximum values appear in opposite areas to the blue dots (reference estimation).

These results suggest that commonly used ESI validation procedures, limited to idealized simulations of local neural currents, may not accurately assess the actual distortions (Haufe et al., 2013; Haufe and Ewald, 2019). Our simulation of EEG that incorporates realistic local neural currents, estimated from high-density MEG, shows that the leakage and localization error in ESI applied to actual data might be much more severe than expected (Palva et al., 2018; He et al., 2019; Van de Steen et al., 2019). Therefore, future efforts should consider validation benchmarks based on realistic simulations like those described here. When using this benchmark, the ssSBL approach appears to considerably control the effect of distortions with respect to other techniques.

### EEG vs. ECoG inverse solutions

**Figure 8:**
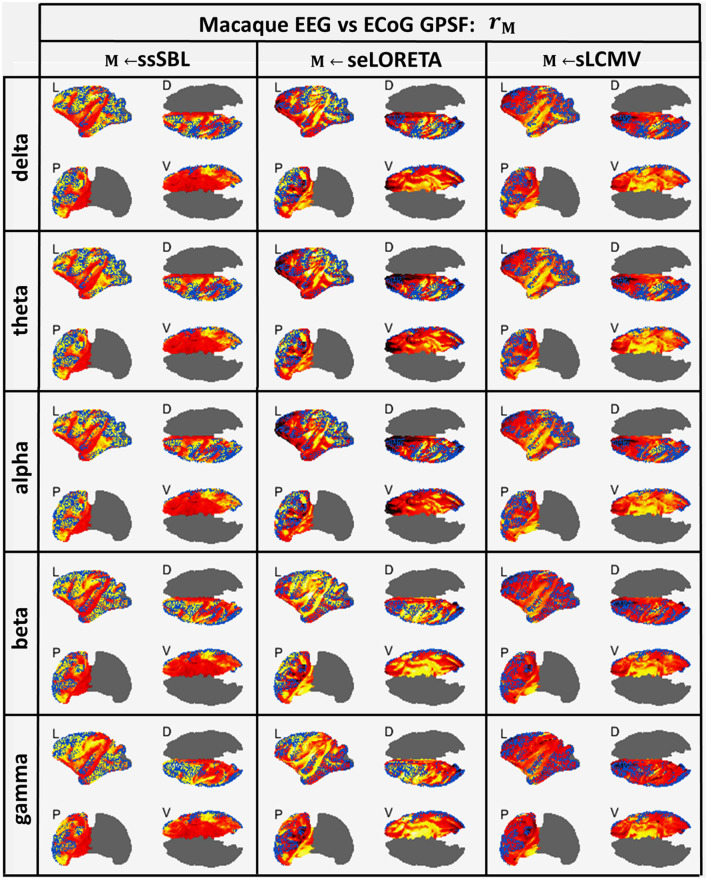
In hot colormaps, measurements of “Leakage” in the EEG-based Cortical Spectral Topographies (CST) were obtained with all tested methods “M” (ssSBL, seLORETA, and sLCMV). Leakage is measured by employing r_*M*_, the Generalized Point Spread Function (GPSF) regarding to the ECoG sMNE reference CST, shown in the cortical views Left (L), Dorsal (D), Posterior (P), and Ventral (V) and for five characteristic spectral bands (delta, theta, alpha, beta, and gamma). Leakage distortions are proportional to mismatch and spread in the GPSF values (hot colormaps) regarding reference points (blue dots). Calculations of the GPSF follow the procedure described in Figure 4 for the ECoGvsEEG experiments.

The GPSF colormaps (Figure 8) for each method showed a consistent behavior as those of the Simulated-EEG vs. MEG experiment (Figure 5). As evident in the GPSF maps **r**_*ssSBL*_ and measured in *b*_*ssSBL*_ (Figure 6, right-column), the performance of ssSBL in reducing leakage was superior compared to sELORETA (*b*_*sELOR*_ > *b*_*ssSBL*_) and sLCMV (*b*_*sLCMV*_ > *b*_*ssSBL*_).

The measurements of incongruence by the EMD *e*_*M*_ (Figure 6, right-column) were consistent with those of the previous experiment (left-column), confirming the improvement of ssSBL regarding sELORETA (*e*_*sELOR*_ > *e*_*ssSBL*_ by a considerably narrow margin) and LCMV (*e*_*sLCMV*_ >> *e*_*ssSBL*_ by a wider margin of three orders of magnitude) for all spectral bands.

**Figure 9:**
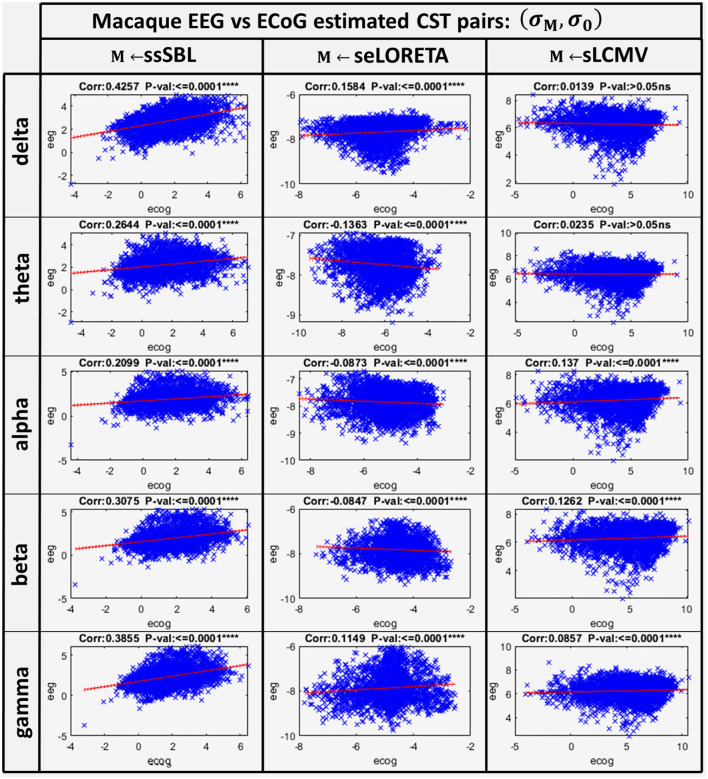
Linear model and correlations for the Cortical Spectral Topographies (CST) obtained from the ECoGvsEEG experiment in the macaque. These were adjusted from the CST data pairs (**σ**_*M*_, **σ**_*M*_) for all tested methods “M” (ssSBL, seLORETA, and sLCMV), and in five characteristic spectral bands (delta, theta, alpha, beta, and gamma).

The correlation distance *c*_*M*_ for all methods showed that linear regression described the relation of EEG CST to ECoG CST well. The correlation was positive for ssSBL but negative for sELORETA and sLCMV as seen in Figure 6 (right-column). As a consequence (*c*_*sLCMV*_ > *c*_*LORETA*>_*c*_*ssSBL*_) for all spectral bands. This linear tendency in Figure 9, similarly to Figure 7 confirms the feasibility of cESI, even with low-density EEG, given its close relationship to a more direct observation modality like ECoG. The results of sELORETA and sLCMV did not reveal a clear linear positive tendency. These results confirm and extend the results of previous studies (Marinazzo et al., 2019; Papadopoulou et al., 2019; Wang et al., 2019), suggesting that some types of ESI should be interpreted with extreme care.

## Discussion

We now summarize and evaluate our results from a theoretical point of view. We introduce a general Bayesian framework for cESI, the estimation of source cross-spectral matrices. This approach allowed us to address the high level of topographic distortions, which arise from the severely ill-conditioned nature of the underlying inverse problem (Kobayashi et al., 2003; Grova et al., 2008; Schoffelen and Gross, 2009; Haufe et al., 2013; Burle et al., 2015; Colclough et al., 2015; Bradley et al., 2016; Mahjoory et al., 2017; Stokes and Purdon, 2017; He et al., 2018, 2019; Palva et al., 2018; Haufe and Ewald, 2019; Marinazzo et al., 2019). We indicated that the problems originating from the ill-posedness were compounded by the habitual implementation of linear and non-sparse (smooth) types of inverse solutions (Mantini et al., 2007; Marzetti et al., 2008; Brookes et al., 2011b, 2012; Hipp et al., 2012; Lopes da Silva, 2013; Colclough et al., 2015; Marinazzo et al., 2019; Nolte et al., 2020).

Non-linear and sparse inverse solutions might ameliorate distortions (Tibshirani, 1996; Zou and Hastie, 2005; Yuan and Lin, 2006; Haufe et al., 2008; Vega-Hernández et al., 2008; Kowalski and Torrésani, 2009a,b; Li and Tian, 2011; Gramfort et al., 2012, 2013). Unfortunately, they may also introduce warping and other biases of the cross-spectral (cESI) estimator. ESI practice with these inverse solutions was most beneficial when addressing the deterministic time/frequency waveforms of event-related brain processes within the framework of spatially short-scale distributed event-related brain networks (Picton and Hillyard, 1974; Picton et al., 1974; Lopes da Silva et al., 1991; Clark et al., 1994; Makeig et al., 1999, 2004; Makeig, 2002; Eichele et al., 2005; Harrison et al., 2008; Vega-Hernández et al., 2008; Maurer and Dierks, 2012). This approach to ESI empiricism suggests that the best results were obtained with flexible smooth/sparse a priori models (Zou and Hastie, 2005; Vega-Hernández et al., 2008; Li and Lin, 2010). Quasilinear inverse solutions of these smooth/sparse models can provide good data-driven approximations.

We formalized the three possible *routes* toward cESI (our target) *via* MAP1 inverse solutions, for the vector processes or their Fourier transform and *via* MAP2 or joint-MAP inverse solutions for their cross-spectrum. The cross-spectral MAP2 or joint-MAP are plausible inverse solutions, which target the quantity of interest and theretofore posit the *a priori* upon the cross-spectrum **Σ**_***ιι***_(*f*).

In contrast, most cESI have been previously limited to route 1 for the processes or route 2 for the Fourier transform (aESI) wherein the *a priori* is placed upon ***ι***(*t*) or ***ι***(*f*). Therefore, essential statistics like cross-spectrum were incorrectly addressed as the subsequent step to aESI. As we demonstrated in simulations and real data, the aESI procedure is not suitable, with cESI amplifying the distortions previously produced during aESI. Indeed, a way to achieve statistical guarantees in cESI is through *route 3* associated with the source cross-spectrum for the data.

We have deferred for now to the complete implementation of the MAP2 inverse solution for the cross-spectrum. This MAP2, which targets a source matrix, was not straightforward, given a cESI setup that is severely ill-conditioned and of high dimensionality. Hence, we adopted the joint-MAP, as an approximation to the MAP2, which targets ${\bar{\Sigma }}_{\iota \iota }\left(f\right)$ of the sampled source cross-spectrum.

An essential concept introducing any MAP inverse solution was the Gibbs energy, a concept first formulated as the Gibbs free energy of any physical system (Landau and Lifshitz, 1980). From the point of view of the inverse problem theory, the Gibbs free energy describes the forward energy exchange of a system (the source variable) to the media (the data) (Ghahramani and Beal, 2001; Grave de Peralta Menendez et al., 2004; Friston et al., 2006; Mattout et al., 2006; Friston K. J. et al., 2008; Friston K. L. et al., 2008; Wipf and Nagarajan, 2009). Thus, this is an important generalization of the Tikhonov regularization and ESI for source cross-spectrum.

We employed this cross-spectral Gibbs energy to model the *joint a priori* probabilities and second-order multivariate properties of the Fourier transform. The Gibbs energy in cESI must be a function of the cross-spectrum entries. This assumption follows from the Gaussianity of Fourier transform (Brillinger, 1965; Brillinger and Rosenblatt, 1967), or statistical sufficiency of the cross-spectrum. This assumption is valid for ESI under a variety of experimental conditions, as it can be demonstrated with high-quality MEG and ECoG data (Nagasaka et al., 2011; Van Essen et al., 2013).

Quasilinear inverse solutions preserved the F-invariance for Gaussianity, avoiding warping of cross-spectral amplitude and phase information (Marzetti et al., 2008; He et al., 2019; Nolte et al., 2020). F-invariance is also valid for a MAP1 assuming Gaussianity of the Fourier transform, and streamlining the dimensionality reduction for MAP2, leading to our joint-MAP interpretation (Hsiao et al., 1998, 2002; Yeredor, 2000; Davis et al., 2001; Auranen et al., 2005; Chen et al., 2011).

We indicate the importance of the nuclear norm (trace) and nuclear quasinorm (square root trace) for matrices, from the context of matrix completion inverse problems (Fan, 1951; Ding et al., 2006; Sun and Zhang, 2012; Chen et al., 2013; Schatten, 2013; Kim et al., 2015; Zhang et al., 2017) which translated into a sparse/smooth spectrum model. Per the definition of cross-spectrum upon vector basis, this model indeed could be unified with the structured vector norms (Kowalski and Torrésani, 2009a,b; Gramfort et al., 2012, 2013). Furthermore, this has been very common in aESI with sparse (LASSO) (Tibshirani, 1996) and smooth (MNE) (Hoerl and Kennard, 1970) model. Our sparse-smooth model was, therefore, an Elastic Net nuclear quasinorm.

Our approach has an important connection to Bayesian learning (Tipping, 2001; Wipf et al., 2006; Park and Casella, 2008; Wipf and Nagarajan, 2009; Casella et al., 2010; Li and Lin, 2010; Li et al., 2011; Paz-Linares et al., 2017) and provided the link between the joint-MAP and quasilinear inverse solutions. We introduced a type of variational approximation to the *joint a priori* probability upon cross-spectral Gibbs energy. This variational approximation is similar to the previous Bayesian Learning methods with extended applicability to high dimensional inverse problems that are solved in iterated conditional mode (Pearl, 1988, 2022; Weiss, 2001; Yedidia et al., 2003; Zheng et al., 2015).

Here, we restricted ourselves to the Gaussian Mixture Model approach (Blei and Jordan, 2006; Bryant and Sudderth, 2012; Gershman and Blei, 2012; Gershman et al., 2012; Nguyen and Bonilla, 2013; Duvenaud et al., 2016) applied to the specific *a priori* per the definition of the Elastic Net nuclear quasinorm (Candes and Recht, 2012; Hu et al., 2012). Our Bayesian learning method (ssSBL) was a generalization of the SBL and sSBL approaches.

Studying the distortions in cESI required a validation based on a reference estimation (ground truth) closer to actual source distributions in the brain rather than simulations of brain electrical activity based on ideal source configurations (Kobayashi et al., 2003; Grova et al., 2008; Schoffelen and Gross, 2009; Haufe et al., 2013; Burle et al., 2015; Colclough et al., 2015; Bradley et al., 2016; Mahjoory et al., 2017; Stokes and Purdon, 2017; Palva et al., 2018; Haufe and Ewald, 2019; Marinazzo et al., 2019).

We demonstrated with several quality measures (Grova et al., 2006b; Haufe et al., 2008; Paz-Linares et al., 2017; Van de Steen et al., 2019; Wang et al., 2019) that cESI estimator distortions in actual data are more larger than expected for some state-of-the-art methods, such as sELORETA and LCMV. Our Simulated-EEG vs. MEG and EEG vs. ECoG validation method benchmarked the cESI distortions in the 10–20 EEG system (19 channels), which is considered the lower bound for all ESI. Therefore, we can infer that the behavior of our methods in EEG or other techniques that provide denser recordings can only improve.

The human Simulated-EEG vs. MEG validation method produces measurements of the cESI estimator distortions very similar to those of the EEG vs. ECoG simultaneously recorded in the macaque (Nagasaka et al., 2011; Wang et al., 2019). This similarity strongly supports the possibility of effectively measuring the distortions expected in many real-life scenarios with our Simulated-EEG vs. MEG design, which is easily replicable for other MEG acquisitions or different experimental conditions (not only resting state).

Our results indicate that ssSBL produces less cESI distortions than sELORETA and sLCMV, according to all leakage measures computed for the Simulated-EEG vs. MEG. These results were supported by cESI obtained from low-density EEG recordings in macaque compared to more fine-grained cESI obtained from simultaneously recorded high-density ECoG.

## Conclusion

In this manuscript, we leveraged Bayesian theory to investigate the benefits and shortcomings of facing cESI with a large family of methods. We show that cESI must be taken care of in severely ill-conditioned and high-dimensional settings such as the ones dealt with here. In such settings, achieving cESI *via* exact Bayesian MAP2 methods is unfeasible and requires approximations. We have introduced quasilinearity, a reasonable cESI assumption, leading to the joint MAP that is feasible *via* the variational approximation to MAP. Our implementation, ssSBL specifies a priori to diminish CST distortions and exhibits good properties, outperforming state-of-the-art methods. The Bayesian theory and methods presented here could potentially be applied to signal processing and imaging other biological phenomena described by the cross-spectrum.

## Data availability statement

The original contributions presented in the study are included in the article/Supplementary material, further inquiries can be directed to the corresponding author/s.

## Author contributions

DP-L and EG-M contributed in developing the theory, mathematical demonstrations, design of methods, implementation, and paper preparation. AA-G, ML, and YW prepared part of the data, code, and results used in this work. MV-H, QW, and EM-M prepared the macaque data for experimental confirmation and contributed to the theory and methods. MB-V, JB-B, and MV-S as senior authors analyzed the results. PV-S as senior author, introduced the theoretical background, CNS program of and guided this work. All authors contributed to the article and approved the submitted version.
