# Supplementary material for: Minimizing the distortions in electrophysiological source imaging of cortical oscillatory activity via Spectral Structured Sparse Bayesian Learning
Source: Front Neurosci. 2023 Mar 15;17:978527. doi: 10.3389/fnins.2023.978527 (PMC10050575; doi:10.3389/fnins.2023.978527)
Supplement: Supplementary file 1 [file Data_Sheet_1.docx]

**Supplementary materials**

# Probability and stochastic processes theory

We include the basic probability theory definitions and properties of stochastic processes from the literature for easy reference (Capiński and Kopp 2004; Durrett 2019; Steele 2001; Hui-Hsiung 2006; Arnold 1974, 1995; Lehmann and Casella 2006).

**Def. 1.** Consider a randomized experiment as the construct upon an abstract probability space defined by the triad . A probability space includes the sample space or set of all possible random outcomes, the sigma-algebra or set of all possible events, and the probability measure with . A *Random variable,* which we denote as , is the mathematical construct for experimental observables, a real-valued *measurable function* with (in ).

*A real-valued *measurable function* is such that, for any Borel set with Borel sigma-algebra of , the inverse function is contained in sigma-algebra (measurable by probability ).

**A Borel sigma-algebra of is the minimal sigma-algebra containing , the set of semi-bounded intervals with .

**Def. 2.** *Cumulative distribution* of a *random variable* , denoted as in the equation Eq. , is the probability induced by inverse function over the semi-bounded intervals. *The probability density* of the *random variable*, denoted as , is the derivative, with respect to , of the *cumulative distribution* :

*** Consider another *random variable*. We may introduce the *joint* *cumulative distribution,* denoted by as in the equation Eq. for a pair of *random variables* , as a real-valued *measurable vector function* with (in ). In other words, a *joint* *cumulative distribution* is a probability induced by an inverse function (or equivalently ) over the semi-bounded rectangles. The *Joint probability density* of the pair of *random variables* , denoted as , is the mixed derivative with respect to of the *cumulative distribution* .

**The *Conditional cumulative distribution,* denoted as in the equation , of a random variable with respect to another random variable , is the probability that is induced by the subtraction of inverse function from over semi-bounded rectangles . *Conditional probability density* of random variable with respect to which we denote as is a more technical definition that instead of the probability considers over a sufficiently narrow rectangle . Then is just the derivative of with respect to . In the probability theory literature is also Radon-Nikodym derivative of the probability induced by inverse function with respect to the probability induced by inverse function over Borel sets .

*** Baye’s theorem for the *conditional cumulative distributions* (or *probability densities* ) is a critical consequence of the equation Eq. that reads as the equation Eq. .

**Def. 3.** -th order moment of a cumulative distribution (or probability density ) is the expected value Eq. of the random variable to a natural number . The first-order moment is the expected value (mean value) , and the second-order central moment computed around is the variance .

**a vital assumption so that all the moments are finite for all . This assumption of finite moments need not cause concern, for all the random variables available in experimental practice appear to be strictly bounded , so all the moments exist.

**Def. 4.** *Sample* is the collection of random outcomes with index counting up to a finite number (sample size) of independent realizations of the randomized experiment . For a *random variable,* , this set is , or simply with the index substituting .

*We employ the sample to determine statistics such as the unbiased estimator -th order moment Eq. . An estimator of the -th order moment is the expected value computed over a sample with , and which distinguishes from computed over (or probability density ). The first-order moment estimator is the sample mean .The second-order central moment , computed around , is the variance estimator .

**Def. 5.** *Random vector,* denoted as , (boldfaced letter) is a real-valued *measurable vector function* that comprises entries with counting a finite collection of *random variables* and with .

*A real-valued *measurable vector function* is such that, for any Borel set with Borel sigma-algebra of , the inverse function is contained in sigma-algebra (measurable by probability ).

**A Borel sigma-algebra of is the minimal sigma-algebra containing with , the set of -dimensional semi-bounded hyper-rectangles (-dimensional cartesian product of semi-bounded intervals) .

***We may consider an essential generalization of a *random vector*, a *random field* , a collection of *random variables* (real-valued *measurable functions*) with a vector index over the 3-dimensional continuous spatial domain , and with . The rigorous definition of requires the extension of Borel sigma-algebra to the continuous function space that we do not include here. However, in neuroimaging practice, we are limited to a discrete spatial domain with , and with index counting recording points. In these points, a neuroimage or random vector is defined as , a finite sub-collection from the *random field* . For simplicity, we use a short notation when referring to the sub-collection.

**Def. 6.** *Cumulative distribution* of a *random vector* , denoted as in the equation Eq. , is the probability that is induced by inverse function over semi-bounded hyper-rectangles . *The probability density* of a *random vector* denoted as is the mixed derivative with respect to of the *cumulative distribution* .

**Def. 7.** The definition of a *Vector stochastic process*, denoted as , employing a second-dimension index that shall appear by default and which is different from the index that counts the vector first-dimension, is the collection of *random vectors* with over the continuous time domain and with .

*An alternative definition of a *vector stochastic process* is the sub-collection (in vector notation with ) from a *dynamic random field* , collection of real-valued measurable functions with and .

**The set of entries of a *vector stochastic process* adjust to the nominal definition of *stochastic process*, i.e. for each entry , the collection of real-valued *measurable functions* with over the continuous time domain and with .

***Note that defining a *vector stochastic process* is very common in continuous time domain , but doing so with suits much better to neuroimaging practice. Also note that a rigorous definition for is through measurable functions over a Filtration, or a process of increasing sigma-algebras in time, so that the stochastic process represents a causal system.

**Def. 8.** *A Vector time series* which we denote as is the countable (finite in neuroimaging practice) sub-collection from a *vector stochastic process* over discrete time domain with and with index counting time points. In other words, *vector time series* which we denote is a real-valued *measurable matrix function* with .

*For the sake of simplicity, we use the short notation when referring to a *vector time series* . Hence, when referring to the physical time we calculate it as , increasing according to a sampling period . Note that we may also refer to or as a more general sub-collection with index counting time points that do not increase with a fixed sampling period. Also note that one could consider vector time series with an infinite number of time points as but this would require *measurable functions* defined over an infinite codomain.

**A real-valued *measurable matrix function* (analogously to previous *measurable vector function*) is such that, for any Borel set with Borel sigma-algebra of , the inverse function is contained in sigma-algebra (measurable by probability ).

***Borel sigma-algebra of is (analogously to previous Borel sigma-algebra of ) the minimal sigma-algebra containing with , the set of all -dimensional semi-bounded hyper-rectangles (-dimensional cartesian product of -dimensional semi-bounded hyper-rectangles) .

**Def. 9.** *Cumulative distribution* of a *vector time series* or simply sub-collection (in other literature *finite-dimensional cumulative distribution* of a *vector stochastic process* ) which we denote as Eq. is the probability that is induced by inverse function over semi-bounded hyper-rectangles . *Probability density* of a *vector time series* (finite-dimensional *probability density* of a *vector stochastic process* ) which we denote as is the mixed derivative with respect to of the *cumulative distribution* .

*We can now introduce the important condition of *stationarity*. A *vector stochastic process* with is *strictly stationary* if the *probability densities* (or equivalently *cumulative distributions*) of all possible sub-collections (not only sub-collections of *vector time series*) with index are translationally invariant, i.e. the identity Eq. holds for all .

**Note that translational invariance Eq. also applies to all possible *vector time series* with physical time and index which may be defined from the *vector stochastic process* modifying the values of , and . A translationally invariant *probability density* (or equivalently *cumulative distribution*) then, is so that for each value , and defining *vector time series* the identity Eq. holds for all .

***Stationarity is a strong condition but seems to be approximately valid for conditions valid during resting-state electrophysiological studies (Damoiseaux et al. 2006; Greicius 2008; Smith et al. 2013) or a task in a block design (Larson-Prior et al. 2013; Van Essen et al. 2013). It may be substituted by the assumption of *local stationarity*.

**Def. 10.** One may extend the concept of moments, from a *random variable* to a *dynamic random field*, a collection of *random variables* over the continuous spatial domain and the time domain . This extension is the essential concept of the multivariate statistics designated *cumulant* , an -th moment computed over the type of sub-collection with counting a finite set of points in space and time from a *dynamic random field* .

*The cumulant may simply be understood as the -th moment that is computed over with fixed , a sub-collection in time domain from a *vector stochastic process* , but yet this definition is too technical to be included here. However, the first-order cumulant and second-order cumulant are the mean value and auto-covariance matrix Eq. that are computed from the corresponding *cumulative distribution* (or *probability density*). In the notation of *vector time series,* the cumulant notation reads as . Thus, equation Eq. with and with reads as follows Eq. .

**Assume the vector stochastic process *strictly stationary*. Then, the *cumulative distributions* and the cumulants that are defined for a sub-collection , are translationally invariant. Thus, expressing the *cumulative distributions* or the *cumulants* we may arbitrarily omit one of the time points say with . The cumulants are then just a function of that we denote as subspace Eq. with exclusion of that leads to “”. In the notation of *vector time series* with () and we denote the subspace and the equation Eq. reads as Eq. .

*** Another important property is that of mixing of a *vector stochastic process* (or a *dynamic random field* ) indicating that the span of dependence between time points is small. A form of the mixing condition is integrability of the *cumulants* , for any , over the subspace Eq. . The mixing condition also seems reasonable for electrophysiology since predictability of future states from the past is always limited. In the notation of *vector time series* integrable reads as summable over the subspace Eq. .

**Def. 11.** *Trajectories* or sample path, or simply *realizations* of a *vector stochastic process* (also of any sub-collection in time or a *vector time series*) are the sample (also or ). This sample is, as usual, defined over the set of random outcomes with and with index counting up to a number (sample size) of independent realizations of the randomized experiment. The realizations are in short notation (also or ) with the index substituting .

*We may employ the samples of a sub-collection or a *vector time series* to determine statistics such as (short notation for with fixed) or the unbiased estimator of the -th order cumulant or that is also too technical to be included here. The sampled estimator of the first-order cumulant is the mean value estimator , and the second-order cumulant is the auto-covariance matrix estimator Eq. . In the notation of *vector time series* these estimators Eq. read as Eq. .

# gamma-MAP and implementation of ssSBL

***Lemma1 (Andrews and Mallows)***

Let be random variable distributing with the following probability density Eq. df:

where $\mathcal{Z}$ is a normalization constant. Then the following equality holds:

$\frac{1}{\mathcal{Z}}exp\left( -\alpha_{1}\left| x \right|-\alpha_{2}x^{2} \right)=$ $\int_{0}^{+\infty} N\left( x | 0,\lambda\right)TGa\left( \gamma\left| 1/2,1,\left( {\alpha_{1}^{2}}/{4\alpha_{2}},\infty\right) \right. \right)d\gamma$ (4.2)

$$\lambda=\left( 1-{\alpha_{1}^{2}}/{4\alpha_{2}\gamma} \right)/{2\alpha_{2}}$$

where $TGa$ is the Truncated Gamma pdf, with a lower truncation limit ${\alpha_{1}^{2}}/{4\alpha_{2}}$.

***Proof of Lemma1:***

The Normal/Laplace probability density function derived from the Gibbs model with Elastic Net penalization can be rearranged as:

$e^{-\alpha_{1}\left| x \right|}e^{-\alpha_{2}x^{2}}=\frac{2}{\alpha_{1}}e^{-\alpha_{2}x^{2}}La\left( x | \alpha_{1} \right)$ (4.3)

Using the integral representation of the Gaussian scale mixtures based on the Andrews and Mallows lemma for the Laplace term, we can represent the formula [B3] above:

$La\left( x | \alpha_{1} \right)=\int_{0}^{+\infty} N\left( x | 0,y \right)\frac{\alpha_{1}^{2}}{2}e^{-\frac{\alpha_{1}^{2}}{2}y}dy$ (4.4)

Alternatively, writing the Normal distribution explicitly in (A1-4), we obtain:

$e^{-\alpha_{1}\left| x \right|}e^{-\alpha_{2}x^{2}}=\int_{0}^{+\infty} e^{-\alpha_{2}x^{2}}\frac{\alpha_{1}}{\sqrt{2\pi y}}e^{-\frac{x^{2}}{2y}}e^{-\frac{\alpha_{1}^{2}}{2}y}dy$ (4.5)

To further simplify the expression (B5), we can rearrange the term on the right by multiplying and dividing by $\sqrt{1+2\alpha_{2}y}$:

$\alpha_{1}\int_{0}^{+\infty} \frac{1}{\sqrt{1+2\alpha_{2}y}}\frac{1}{\sqrt{2\pi\frac{y}{1+2\alpha_{2}y}}}e^{-\frac{x^{2}}{\frac{2y}{\left( 1+2\alpha_{2}y \right)}}}e^{-\frac{\alpha_{1}^{2}}{2}y}dy$ (4.6)

Through using the following change of variables

$\gamma=\left( {\alpha_{1}^{2}}/{4\alpha_{2}} \right)(1+2\alpha_{2}y)$

$\lambda=y/\left( 1+2\alpha_{2}y \right)$

we arrive at:

$\sqrt{{\alpha_{1}^{2}}/{4\alpha_{2}}}e^{{\alpha_{1}^{2}}/{4\alpha_{2}}}\int_{{\alpha_{1}^{2}}/{4\alpha_{2}}}^{+\infty} \frac{1}{\sqrt{2\pi\lambda}}e^{-\frac{x^{2}}{2\lambda}}\gamma^{-\frac{1}{2}}e^{-\gamma}d\gamma=$

$\int_{{\alpha_{1}^{2}}/{4\alpha_{2}}}^{+\infty} N\left( x | 0,\lambda\right)\sqrt{{\alpha_{1}^{2}}/{4\alpha_{2}}}e^{{\alpha_{1}^{2}}/{4\alpha_{2}}}\gamma^{-\frac{1}{2}}e^{-\gamma}d\gamma$

Then, using the definition for the Gamma probability density function truncated in the interval $\left( {\alpha_{1}^{2}}/{4\alpha_{2}},\infty\right)$, denominated truncated Gamma density:

$TGa\left( \gamma| 1/2,1,\left( {\alpha_{1}^{2}}/{4\alpha_{2}},\infty\right) \right)\propto\gamma^{-\frac{1}{2}}e^{-\gamma}I_{\left( {\alpha_{1}^{2}}/{4\alpha_{2}},\infty\right)}\left( \gamma\right)$

We can finally demonstrate that the Normal/Laplace probability density function can be represented as the following scaled mixture of Gaussians:

$e^{-\alpha_{1}\left| x \right|}e^{-\alpha_{2}x^{2}}\propto\int_{0}^{+\infty} N\left( x | 0,\lambda\right)TGa\left( \gamma| 1/2,1,\left( {\alpha_{1}^{2}}/{4\alpha_{2}},\infty\right) \right)d\gamma$ (4.7)

where $\lambda=\left( 1-{\alpha_{1}^{2}}/{4\alpha_{2}\gamma} \right)/{2\alpha_{2}}$.

***Vector form of Lemma1***

This can be extended for the case in which $x$ is the vector $\boldsymbol{x}$ in the following expression, with $\boldsymbol{\Lambda}=diag\left( \left( 1-{\alpha_{1}^{2}}/{4\alpha_{2}\boldsymbol{\gamma}} \right)/{2\alpha_{2}} \right)$ a matrix function of the vector argument $\boldsymbol{\gamma}$.

$e^{-\alpha_{1}\left\| \boldsymbol{x} \right\|_{1}}e^{-\alpha_{2}\left\| \boldsymbol{x} \right\|_{2}^{2}}\propto\int_{0}^{+\infty} N\left( \boldsymbol{x} | 0,\boldsymbol{\Lambda} \right)TGa\left( \boldsymbol{\gamma} | 1/2,1,\left( {\alpha_{1}^{2}}/{4\alpha_{2}},\infty\right) \right)d\boldsymbol{\gamma}$ (4.8)

where $\left\| \boldsymbol{x} \right\|_{1}$ is the L1 norm and $\left\| \boldsymbol{x} \right\|_{2}^{2}$ is the L2 norm, for the complex-valued vector $\boldsymbol{x}$ and the truncated gamma distribution $TGa$ upon the vector $\boldsymbol{\gamma}$ is expressed as follows:

$TGa\left( \boldsymbol{\gamma} | 1/2,1,\left( {\alpha_{1}^{2}}/{4\alpha_{2}},\infty\right) \right)=\prod_{g} TGa\left( \gamma\left( g \right) | 1/2,1,\left( {\alpha_{1}^{2}}/{4\alpha_{2}},\infty\right) \right)$■ (4.9)

## Extension of the Andrews and Mallows Lemma to the complex-valued hierarchical Elastic Net using measure-densities

The results for the hierarchical Elastic Net can be extended to the complex domain by modifying the Andrews and Mallows Lemma. For the complex-valued Elastic Net, the integral representation holds.

$e^{-\alpha_{1}\left\| \boldsymbol{x} \right\|_{1}}e^{-\alpha_{2}\left\| \boldsymbol{x} \right\|_{2}^{2}}\propto\int N\left( \left| \boldsymbol{x} \right| | 0,\boldsymbol{\Lambda} \right)p\left( \boldsymbol{\gamma} \right)d\boldsymbol{\gamma}$ (4.10)

where the variances are defined as $\boldsymbol{\Lambda}=diag\left( \left( 1-{\alpha_{1}^{2}}/{4\alpha_{2}\boldsymbol{\gamma}} \right)/{2\alpha_{2}} \right)$.

The measurable space in which the variable $\left. \boldsymbol{x} \right|\boldsymbol{\gamma}$ is defined as an unnormalized density function given by the Gaussian pdf $p\left( \boldsymbol{x} | \boldsymbol{\gamma} \right)=N\left( \left| \boldsymbol{x} \right| | 0,\boldsymbol{\Lambda} \right)$ and its variance $\boldsymbol{\Lambda}$ is dependent on the random variable $\gamma$ which has Truncated Gamma pdf.

$p\left( \boldsymbol{\gamma} \right)=TGa\left( \boldsymbol{\gamma} | 1/2,1,\left( {\alpha_{1}^{2}}/{4\alpha_{2}},\infty\right) \right)$ (4.11)

Then, the measure in the space product of $\boldsymbol{x}$ and $p\left( \boldsymbol{\gamma} \right)$ is had density represented as an unnormalized product of Gaussian and Gamma densities.

$p\left( \boldsymbol{x},\boldsymbol{\gamma} \right)\propto N\left( \left| \boldsymbol{x} \right| | 0,\boldsymbol{\Lambda} \right)TGa\left( \boldsymbol{\gamma} | 1/2,1,\left( {\alpha_{1}^{2}}/{4\alpha_{2}},\infty\right) \right)$ (4.12)

## Structured space-frequency sparsity modes within the hierarchical complex-valued Elastic-Net

We introduce additional tensor group penalization for the Hierarchical Elastic Net on the 3D cartesian space of generators, samples, and frequencies with:

$\left| x \right|=\sqrt{\sum_{m\in\mathbb{M}} \sum_{\nu\in\mathbb{F}_{b}} \sum_{g\in\mathbb{G}_{\mathcal{a}}} \left| \boldsymbol{\chi}_{\mathcal{m}}\left( \nu;g \right) \right|^{2}}$ (4.13)

where $\boldsymbol{\chi}_{m}\left( \nu\right)=\mathbf{DN}\boldsymbol{\iota}_{m}\left( \nu\right)$, $m\mathbb{\in M}$, $\nu\in\mathbb{F}$; $a$ refers to a specific Gray matter area $a\in\mathbb{A}$; $b$ refers to a specific frequency band $b\in\mathbb{B}$. Then the transformed prior of the parameters is described analytically by the following distribution:

$\left\{ \begin{matrix} \left\{ \boldsymbol{\chi}_{m}\left( \nu;g \right),m\in\mathbb{M}, \nu\in\mathbb{F}_{b}, g\in\mathbb{G}_{\mathcal{a}} \right\}\sim\frac{e^{-\frac{1}{2}\lambda_{\mathcal{a}}^{-1}\sum_{m\in\mathbb{M}} \sum_{\nu\in\mathbb{F}_{b}} \sum_{g\in\mathbb{G}_{\mathcal{a}}} \left| \boldsymbol{\chi}_{m}\left( \nu;g \right) \right|^{2}}}{\left| 2\pi\lambda_{\mathcal{a}} \right|^{\frac{1}{2}}} \\ or \\ \boldsymbol{\chi}_{m}\left( \nu;g \right)\sim\frac{e^{-\frac{1}{2}\boldsymbol{\chi}_{m}^{\dagger}\left( \nu;g \right)\lambda_{\mathcal{a}}^{-1}\boldsymbol{\chi}_{m}\left( \nu;g \right)}}{\left| 2\pi\lambda_{\mathcal{a}} \right|^{\frac{1}{2q_{a}f_{b}m}}},m\in\mathbb{M},\nu\in\mathbb{F}_{b},g\in\mathbb{G}_{a} \end{matrix} \right.$ (4.14)

where $\lambda_{\mathcal{a}}=\left( 1-{\alpha_{1}^{2}}/{4\alpha_{2}\gamma_{a}} \right)/{2\alpha_{2}}$ and $\gamma_{\mathcal{a}}\sim TGa\left( \gamma_{\mathcal{a}}\left| 1/2,1,\left( {\alpha_{1}^{2}}/{4\alpha_{2}},\infty\right) \right. \right)$

In vector form they are expressed as:

$\left\{ \begin{matrix} \boldsymbol{\chi}_{m}\left( \nu\right)\sim N\left( \left| \boldsymbol{\chi}_{\mathcal{m}}\left( \nu\right) \right| | 0,\boldsymbol{\Lambda} \right),m\in\mathbb{M},\nu\in\mathbb{F}_{\mathcal{b}} \\ or \\ \boldsymbol{\iota}_{m}\left( \nu\right)\sim N\left( \left| \mathbf{DN}\boldsymbol{\iota}_{\mathcal{m}}\left( \nu\right) \right| | 0,\boldsymbol{\Lambda} \right),m\in\mathbb{M},\nu\in\mathbb{F}_{\mathcal{b}} \end{matrix} \right.$ (4.15)

where $\boldsymbol{\Lambda}=diag\left( \left( 1-{\alpha_{1}^{2}}/{4\alpha_{2}\boldsymbol{\gamma}} \right)/{2\alpha_{2}} \right)$ and $\boldsymbol{\gamma}\left( g \right)=\gamma_{a}$, $g\in\mathbb{G}_{a}$, $a\in\mathbb{A}$. The unnormalized distribution upon $\boldsymbol{\chi}_{m}\left( \nu\right)$ is represented as:

$${e^{-\frac{1}{2}\boldsymbol{\chi}_{m}^{\dagger}\left( \nu\right)\boldsymbol{\Lambda}^{-1}\boldsymbol{\chi}_{m}\left( \nu\right)}}/{\left| 2\pi\boldsymbol{\Lambda} \right|^{\frac{1}{2 \left( \prod_{a\in\mathbb{A}} g_{a} \right)f_{b}m}}}$$

where the $g$-th element diagonal element $\boldsymbol{\Lambda}\left( g,g \right)$ are the variances. The full vector Bayesian model is as follows:

$\boldsymbol{v}_{m}\left( \nu\right)\sim N^{\mathbb{C}}\left( \boldsymbol{v}_{m}\left( \nu\right) | \mathbf{L}_{\boldsymbol{v\iota}}\boldsymbol{\iota}_{m}\left( \nu\right),\beta\mathbf{I} \right)$, $m\in\mathbb{M}$, $\nu\in\mathbb{F}_{b}$ (4.16)

$\boldsymbol{\iota}_{m}\left( \nu\right)\sim N\left( \left| \mathbf{DN}\boldsymbol{\iota}_{m}\left( \nu\right) \right| | 0,\boldsymbol{\Lambda} \right)$, $m\in\mathbb{M}$, $\nu\in\mathbb{F}_{b}$ (4.17)

$\boldsymbol{\gamma}\sim\prod_{a\in\mathbb{A}} TGa\left( \gamma_{a}\left| \frac{1}{2},1,\left( \frac{\alpha_{1}^{2}}{4\alpha_{2}},\infty\right) \right. \right)$ (4.18)

where $\boldsymbol{\Lambda}=diag\left( \left( 1-{\alpha_{1}^{2}}/{4\alpha_{2}\boldsymbol{\gamma}} \right)/{2\alpha_{2}} \right)$ and $\boldsymbol{\gamma}\left( g \right)=\gamma_{a}$, $g\in\mathbb{G}_{a}$, $a\in\mathbb{A}$

## Bayesian first type maximum a posteriori (parameters) analysis with the hierarchical complex-valued Elastic-Net prior

**Proposition1:**

For the joint distribution of data and parameters $p\left( \boldsymbol{v}_{\mathcal{m}}\left( \nu\right),\boldsymbol{\iota}_{\mathcal{m}}\left( \nu\right) \right)$ the following factorization holds, for simplicity, we avoid the use of argument for frequency $\nu$ and samples $m$:

$$N^{\mathbb{C}}\left( \boldsymbol{v} | \mathbf{L}_{\boldsymbol{v\iota}}\boldsymbol{\iota},\beta\mathbf{I} \right)N\left( \left| \mathbf{DN}\boldsymbol{\iota} \right| | 0,\boldsymbol{\Lambda} \right)=$$

$\left| \pi\mathbf{P} \right|N^{\mathbb{C}}\left( \boldsymbol{\iota} | \boldsymbol{\mu},\mathbf{P} \right)N^{\mathbb{C}}\left( \boldsymbol{v} | \mathbf{L}_{\boldsymbol{v\iota}}\boldsymbol{\mu},\beta\mathbf{I} \right)N\left( \left| \mathbf{DN}\boldsymbol{\mu} \right| | 0,\boldsymbol{\Lambda} \right)$ (4.19)

The quantities $\boldsymbol{\mu}$ (posterior mean) and $\mathbf{P}$ (posterior covariance) are defined as follows:

$\boldsymbol{\mu}=\beta^{-1}\mathbf{P}\mathbf{L}_{\boldsymbol{\iota v}}\boldsymbol{v}$ (4.20)

$\mathbf{P}=\left( \beta^{-1}\mathbf{L}_{\boldsymbol{\iota v}}\mathbf{L}_{\boldsymbol{v\iota}}+\frac{1}{2}\left( \mathbf{DN} \right)^{\mathcal{T}}\boldsymbol{\Lambda}^{-1}\mathbf{DN} \right)^{-1}$ (4.21)

The quantity $\mathbf{F}$ (ensemble covariance) is given by:

$\boldsymbol{\Lambda}=diag\left( \left( 1-{\alpha_{1}^{2}}/{4\alpha_{2}\boldsymbol{\gamma}} \right)/{2\alpha_{2}} \right)$ (4.22)

Note that we avoid using the frequency argument $\nu$, used to define the model in (4.19], for the sake of the readability of the derivations.

**Proof of Proposition1**

This proposition can be demonstrated by writing their distributions explicitly.

$N^{\mathbb{C}}\left( \boldsymbol{v} | \mathbf{L}_{\boldsymbol{v\iota}}\boldsymbol{\iota},\beta\mathbf{I} \right)=\frac{e^{-\beta^{-1}\left( \boldsymbol{v}-\mathbf{L}_{\boldsymbol{v\iota}}\boldsymbol{\iota} \right)^{\dagger}\left( \boldsymbol{v}-\mathbf{L}_{\boldsymbol{v\iota}}\boldsymbol{\iota} \right)}}{\left| \pi\beta\mathbf{I} \right|}$

$N\left( \left| \mathbf{DN}\boldsymbol{\iota} \right| | 0,\boldsymbol{\Lambda} \right)=\frac{e^{-\frac{1}{2}\boldsymbol{\iota}^{\dagger}\left( \mathbf{DN} \right)^{\mathcal{T}}\boldsymbol{\Lambda}^{-1}\mathbf{DN}\boldsymbol{\iota}}}{\left| 2\pi\boldsymbol{\Lambda} \right|^{\frac{1}{2 \left( \prod_{\mathcal{a\in}\mathbb{A}} g_{\mathcal{a}} \right)f_{\mathcal{b}}m}}}$

The form of the resultant distribution can be found by analyzing the terms that depend on the parameters (exponential argument) in the formula above:

$-\beta^{-1}\boldsymbol{v}^{\dagger}\boldsymbol{v}-\boldsymbol{\iota}^{\dagger}\mathbf{L}_{\boldsymbol{\iota v}}\mathbf{L}_{\boldsymbol{v\iota}}\boldsymbol{\iota}+\beta^{-1}\boldsymbol{v}^{\dagger}\mathbf{L}_{\boldsymbol{v\iota}}\boldsymbol{\iota}+\beta^{-1}\boldsymbol{\iota}^{\dagger}\mathbf{L}_{\boldsymbol{\iota v}}\boldsymbol{v}-\frac{1}{2}\boldsymbol{\iota}^{\dagger}\left( \mathbf{DN} \right)^{\mathcal{T}}\boldsymbol{\Lambda}^{-1}\mathbf{DN}\boldsymbol{\iota}$ (4.23)

Reorganizing in (4.23] $\beta^{-1}\mathbf{L}_{\boldsymbol{\iota v}}\mathbf{L}_{\boldsymbol{v\iota}}+\frac{1}{2}\left( \mathbf{DN} \right)^{\mathcal{T}}\boldsymbol{\Lambda}^{-1}\mathbf{DN}$ of terms 2 and 5 to render $\mathbf{P}^{-1}$ and $\beta^{-1}\mathbf{L}_{\boldsymbol{\iota v}}\boldsymbol{v}$ in terms 3 and 4 to render $\boldsymbol{\mu}$

$-\beta^{-1}\boldsymbol{v}^{\dagger}\boldsymbol{v}+\left( \beta^{-1}\mathbf{P}\mathbf{L}_{\boldsymbol{\iota v}}\boldsymbol{v} \right)^{\dagger}\mathbf{P}^{-1}\boldsymbol{\iota}+\boldsymbol{\iota}^{\dagger}\mathbf{P}^{-1}\left( \beta^{-1}\mathbf{P}\mathbf{L}_{\boldsymbol{\iota v}}\boldsymbol{v} \right)-\boldsymbol{\iota}^{\dagger}\left( \beta^{-1}\mathbf{L}_{\boldsymbol{\iota v}}\mathbf{L}_{\boldsymbol{v\iota}}\boldsymbol{+}\frac{1}{2}\left( \mathbf{DN} \right)^{\mathcal{T}}\boldsymbol{\Lambda}^{-1}\mathbf{DN} \right)\boldsymbol{\iota}$ (4.24)

From (4.24] based on $\mathbf{P}^{-1}$ and $\boldsymbol{\mu}$ we obtain

$-\beta^{-1}\boldsymbol{v}^{\dagger}\boldsymbol{v}+\boldsymbol{\mu}^{\dagger}\mathbf{P}^{-1}\boldsymbol{\iota}+\boldsymbol{\iota}^{\dagger}\mathbf{P}^{-1}\boldsymbol{\mu}-\boldsymbol{\iota}^{\dagger}\mathbf{P}^{-1}\boldsymbol{\iota}$ (4.25**)**

Completing (4.25] with the term $+\boldsymbol{\mu}^{\dagger}\mathbf{P}^{-1}\boldsymbol{\mu}$

$-\left( \boldsymbol{\iota}-\boldsymbol{\mu} \right)^{\dagger}\mathbf{P}^{-1}\left( \boldsymbol{\iota}-\boldsymbol{\mu} \right)-\beta^{-1}\boldsymbol{v}^{\dagger}\boldsymbol{v}+\boldsymbol{\mu}^{\dagger}\mathbf{P}^{-1}\boldsymbol{\mu}$ (4.26)

Completing (4.26] with the terms $-\beta^{-1}\boldsymbol{v}^{\dagger}\mathbf{L}_{\boldsymbol{v\iota}}\boldsymbol{\mu}$, $-\beta^{-1}\boldsymbol{\mu}^{\dagger}\mathbf{L}_{\boldsymbol{\iota v}}\boldsymbol{v}$ and $+\beta^{-1}\boldsymbol{\mu}^{\dagger}\mathbf{L}_{\boldsymbol{\iota v}}\mathbf{L}_{\boldsymbol{v\iota}}\boldsymbol{\mu}$ we obtain:

$-\beta^{-1}\left( \boldsymbol{\iota}-\boldsymbol{\mu} \right)^{\dagger}\mathbf{P}^{-1}\left( \boldsymbol{\iota}-\boldsymbol{\mu} \right)-\beta^{-1}\left( \boldsymbol{v}-\mathbf{L}_{\boldsymbol{v\iota}}\boldsymbol{\mu} \right)^{\dagger}\left( \boldsymbol{v}-\mathbf{L}_{\boldsymbol{v\iota}}\boldsymbol{\mu} \right)-\beta^{-1}\boldsymbol{v}^{\dagger}\mathbf{L}_{\boldsymbol{v\iota}}\boldsymbol{\mu-}\beta^{-1}\boldsymbol{\mu}^{\dagger}\mathbf{L}_{\boldsymbol{v\iota}}^{\mathcal{T}}\boldsymbol{v}+\beta^{-1}\boldsymbol{\mu}^{\dagger}\mathbf{L}_{\boldsymbol{\iota v}}\mathbf{L}_{\boldsymbol{v\iota}}\boldsymbol{\mu}+\boldsymbol{\mu}^{\dagger}\mathbf{P}^{-1}\boldsymbol{\mu}$ (4.27)

Then terms 3, 4, and 6 in (4.27] can be reorganized into $-\boldsymbol{\mu}^{\dagger}\mathbf{P}^{-1}\boldsymbol{\mu}$ since $\beta^{-1}\mathbf{L}_{\boldsymbol{v\iota}}^{\mathcal{T}}\boldsymbol{v}=\mathbf{P}^{-1}\boldsymbol{\mu}^{\dagger}$ to obtain:

$-\left( \boldsymbol{\iota}-\boldsymbol{\mu} \right)^{\dagger}\mathbf{P}^{-1}\left( \boldsymbol{\iota}-\boldsymbol{\mu} \right)-\beta^{-1}\left( \boldsymbol{v}-\mathbf{L}_{\boldsymbol{v\iota}}\boldsymbol{\mu} \right)^{\dagger}\left( \boldsymbol{v}-\mathbf{L}_{\boldsymbol{v\iota}}\boldsymbol{\mu} \right)-\boldsymbol{\mu}^{\dagger}\mathbf{P}^{-1}\boldsymbol{\mu}+\beta^{-1}\boldsymbol{\mu}^{\dagger}\mathbf{L}_{\boldsymbol{\iota v}}\mathbf{L}_{\boldsymbol{v\iota}}\boldsymbol{\mu}$ (4.28]

But combining terms 3 and 4 in (4.28] yields $-\frac{1}{2}\boldsymbol{\mu}^{\dagger}\left( \mathbf{DN} \right)^{\mathcal{T}}\boldsymbol{\Lambda}^{-1}\mathbf{DN}\boldsymbol{\mu}$

$-\left( \boldsymbol{\iota}-\boldsymbol{\mu} \right)^{\dagger}\mathbf{P}^{-1}\left( \boldsymbol{\iota}-\boldsymbol{\mu} \right)\beta^{-1}\left( \boldsymbol{v}-\mathbf{L}_{\boldsymbol{v\iota}}\boldsymbol{\mu} \right)^{\dagger}\left( \boldsymbol{v}-\mathbf{L}_{\boldsymbol{v\iota}}\boldsymbol{\mu} \right)-\frac{1}{2}\boldsymbol{\mu}^{\dagger}\left( \mathbf{DN} \right)^{\mathcal{T}}\boldsymbol{\Lambda}^{-1}\mathbf{DN}\boldsymbol{\mu}$ (4.29)

From (4.29], it holds that:

$$N^{\mathbb{C}}\left( \boldsymbol{v} | \mathbf{L}_{\boldsymbol{v\iota}}\boldsymbol{\iota},\beta\mathbf{I} \right)N\left( \left| \mathbf{DN}\boldsymbol{\iota} \right| | 0,\boldsymbol{\Lambda} \right)=$$

$\left| \pi\mathbf{P} \right|N^{\mathbb{C}}\left( \boldsymbol{\iota} | \boldsymbol{\mu},\mathbf{P} \right)N^{\mathbb{C}}\left( \boldsymbol{v} | \mathbf{L}_{\boldsymbol{v\iota}}\boldsymbol{\mu},\beta\mathbf{I} \right)N\left( \left| \mathbf{DN}\boldsymbol{\mu} \right| | 0,\boldsymbol{\Lambda} \right)$ (4.30)

## Bayesian second type maximum a posteriori (hyperparameters) analysis with the hierarchical complex-valued Elastic Net prior

From the joint distribution $p\left( \left\{ \left( \boldsymbol{v}_{\mathcal{m}}\left( \nu\right),\boldsymbol{\iota}_{\mathcal{m}}\left( \nu\right) \right), \mathcal{m}\in\mathbb{M}, \nu\in\mathbb{F}_{b} \right\} \right)$ we can derive iteratively and approximated representation of the Type II-Likelihood $p^{\left( k \right)}\left( \left\{ \boldsymbol{v}_{\mathcal{m}}\left( \nu\right), \mathcal{m}\in\mathbb{M}, \nu\in\mathbb{F}_{\mathcal{b}} \right\} \right)$:

$p\left( \left\{ \boldsymbol{v}_{\mathcal{m}}\left( \nu\right), \mathcal{m}\in\mathbb{M}, \nu\in\mathbb{F}_{\mathcal{b}} \right\} \right)=\prod_{\nu\in\mathbb{F}_{\mathcal{b}}} \prod_{\mathcal{m}\in\mathbb{M}} \int p\left( \boldsymbol{v}_{\mathcal{m}}\left( \nu\right),\boldsymbol{\iota}_{\mathcal{m}}\left( \nu\right) \right)d\boldsymbol{\iota}_{\mathcal{m}}\left( \nu\right)$ (4.31)

$p\left( \left\{ \boldsymbol{v}_{\mathcal{m}}\left( \nu\right), \mathcal{m}\in\mathbb{M}, \nu\in\mathbb{F}_{\mathcal{b}} \right\} \right)\approx p^{\left( k \right)}\left( \left\{ \boldsymbol{v}_{\mathcal{m}}\left( \nu\right), \mathcal{m}\in\mathbb{M}, \nu\in\mathbb{F}_{\mathcal{b}} \right\} \right)$ (4.32)

where

$$p^{\left( k \right)}\left( \left\{ \boldsymbol{v}_{\mathcal{m}}\left( \nu\right), \mathcal{m}\in\mathbb{M}, \nu\in\mathbb{F}_{\mathcal{b}} \right\} \right)=$$

$\prod_{\nu\in\mathbb{F}_{\mathcal{b}}} \prod_{\mathcal{m}\in\mathbb{M}} \int N^{\mathbb{C}}\left( \boldsymbol{v}_{\mathcal{m}}\left( \nu\right) | \mathbf{L}_{\boldsymbol{v\iota}}\boldsymbol{\iota}_{\mathcal{m}}\left( \nu\right),\beta\mathbf{I} \right)N\left( \left| \mathbf{DN}\boldsymbol{\iota}_{\mathcal{m}}\left( \nu\right) \right| | 0,\boldsymbol{\Lambda} \right)d\boldsymbol{\iota}_{\mathcal{m}}\left( \nu\right)$ (4.33)

The analysis of the previous section yields

$\int N_{p}^{\mathbb{C}}\left( \boldsymbol{v}_{\mathcal{m}}\left( \nu\right) | \mathbf{L}_{\boldsymbol{v\iota}}\boldsymbol{\iota}_{\mathcal{m}}\left( \nu\right),\beta\mathbf{I} \right)N\left( \left| \mathbf{DN}\boldsymbol{\iota}_{\mathcal{m}}\left( \nu\right) \right| | 0,\boldsymbol{\Lambda} \right)d\boldsymbol{\iota}_{\mathcal{m}}\left( \nu\right)=$

$\left| \pi\mathbf{P} \right|N^{\mathbb{C}}\left( \boldsymbol{v}_{\mathcal{m}}\left( \nu\right) | \mathbf{L}_{\boldsymbol{v\iota}}\boldsymbol{\mu}_{\mathcal{m}}^{\left( k \right)}\left( \nu\right),\beta\mathbf{I} \right)N\left( \left| \mathbf{DN}\boldsymbol{\mu}_{\mathcal{m}}^{\left( k \right)}\left( \nu\right) \right| | 0,\boldsymbol{\Lambda} \right)$ (4.34)

Then iteratively upon fixed values of the posterior mean $\boldsymbol{\mu}_{\mathcal{m}}^{\left( k \right)}\left( \nu\right)$ the Type II-Likelihood is expressed as:

$p^{\left( k \right)}\left( \left\{ \boldsymbol{v}_{\mathcal{m}}\left( \nu\right), \mathcal{m}\in\mathbb{M}, \nu\in\mathbb{F}_{\mathcal{b}} \right\} \right)=$

$\left\{ \begin{matrix} \begin{matrix} \prod_{\nu\in\mathbb{F}_{\mathcal{b}}} \prod_{\mathcal{m}\in\mathbb{M}} \left| \pi\mathbf{P} \right|N^{\mathbb{C}}\left( \boldsymbol{v}_{\mathcal{m}}\left( \nu\right) | \mathbf{L}_{\boldsymbol{v\iota}}\boldsymbol{\mu}_{\mathcal{m}}^{\left( k \right)}\left( \nu\right),\beta\mathbf{I} \right)\times\\ N\left( \left| \mathbf{DN}\boldsymbol{\mu}_{\mathcal{m}}^{\left( k \right)}\left( \nu\right) \right| | 0,\boldsymbol{\Lambda} \right) \end{matrix} \\ or \\ \frac{\left| \pi\mathbf{P} \right|^{f_{\mathcal{b}}m}}{\left| \pi\beta\mathbf{I} \right|^{f_{\mathcal{b}}m}\left| 2\pi\boldsymbol{\Lambda} \right|^{\frac{1}{2}}}e^{-\frac{f_{\mathcal{b}}m}{2}tr\left( \boldsymbol{\Lambda}^{-1}\mathbf{DN}{\bar{\boldsymbol{\Sigma}}}_{\boldsymbol{\iota\iota}}^{\left( k \right)}\left( \mathbf{DN} \right)^{\mathcal{T}} \right)}e^{-f_{\mathcal{b}}m\beta tr\left( {\bar{\boldsymbol{\Sigma}}}_{\boldsymbol{\xi\xi}}^{\left( k \right)} \right)} \end{matrix} \right.$ (4.35)

Where the covariances ${\bar{\boldsymbol{\Sigma}}}_{\boldsymbol{\iota\iota}}^{\left( k \right)}$ and ${\bar{\boldsymbol{\Sigma}}}_{\boldsymbol{\xi\xi}}^{\left( k \right)}$ are

${\bar{\boldsymbol{\Sigma}}}_{\boldsymbol{\iota\iota}}^{\left( k \right)}=\frac{1}{f_{\mathcal{b}}m}\sum_{\nu\in\mathbb{F}_{\mathcal{b}}} \sum_{\mathcal{m}\in\mathbb{M}} \boldsymbol{\mu}_{\mathcal{m}}^{\left( k \right)}\left( \nu\right)\left( \boldsymbol{\mu}_{\mathcal{m}}^{\left( k \right)}\left( \nu\right) \right)^{\dagger}$

${\bar{\boldsymbol{\Sigma}}}_{\boldsymbol{\xi\xi}}^{\left( k \right)}=\frac{1}{f_{b}m}\sum_{\nu\in\mathbb{F}_{b}} \sum_{m\in\mathbb{M}} \left( \boldsymbol{v}_{\mathcal{m}}\left( \nu\right)-\mathbf{L}_{\boldsymbol{v\iota}}\boldsymbol{\mu}_{\mathcal{m}}^{\left( k \right)}\left( \nu\right) \right)\left( \boldsymbol{v}_{\mathcal{m}}\left( \nu\right)-\mathbf{L}_{\boldsymbol{v\iota}}\boldsymbol{\mu}_{\mathcal{m}}^{\left( k \right)}\left( \nu\right) \right)^{\dagger}$

***Parameter estimators***

The parameters $\boldsymbol{\mu}_{\mathcal{m}}^{\left( k \right)}\left( \nu\right)$ are determined in the previous iteration in terms of the iteratively linear source transfer operator $\mathbf{T}_{\boldsymbol{\iota v}}^{\left( k \right)}$

$\boldsymbol{\mu}_{\mathcal{m}}^{\left( k \right)}\left( \nu\right)=\mathbf{T}_{\boldsymbol{\iota v}}^{\left( k \right)}\boldsymbol{v}_{\mathcal{m}}\left( \nu\right)$ (4.36)

The source transfer operator is defined upon fixed values of the hyperparameters:

$\mathbf{T}_{\boldsymbol{\iota v}}^{\left( k \right)}={\beta^{\left( k \right)}}^{-1}\mathbf{P}^{\left( k \right)}\mathbf{L}_{\boldsymbol{\iota v}}$ (4.37)

where $\mathbf{P}^{\left( k \right)}=\left( {\beta^{\left( k \right)}}^{-1}\mathbf{L}_{\boldsymbol{\iota v}}\mathbf{L}_{\boldsymbol{v\iota}}+\frac{1}{2}\left( \mathbf{DN} \right)^{\mathcal{T}}{\boldsymbol{\Lambda}^{\left( k \right)}}^{-1}\mathbf{DN} \right)^{-1}$

and $\boldsymbol{\Lambda}^{\left( k \right)}=diag\left( \left( 1-{{\alpha_{1}^{\left( k \right)}}^{2}}/{4\alpha_{2}^{\left( k \right)}\boldsymbol{\gamma}^{\left( k \right)}} \right)/{2\alpha_{2}^{\left( k \right)}} \right)$

This source transfer operator simplifies the computations of ${\bar{\boldsymbol{\Sigma}}}_{\boldsymbol{\iota\iota}}^{\left( k \right)}$ expressed as:

${\bar{\boldsymbol{\Sigma}}}_{\boldsymbol{\iota\iota}}^{\left( k \right)}=\mathbf{T}_{\boldsymbol{\iota v}}^{\left( k \right)}{\hat{\boldsymbol{\Sigma}}}_{vv}\mathbf{T}_{\boldsymbol{v\iota}}^{\left( k \right)}$ (4.38)

where ${\hat{\boldsymbol{\Sigma}}}_{vv}=\frac{1}{f_{\mathcal{b}}m}\sum_{\nu\in\mathbb{F}_{\mathcal{b}}} \sum_{\mathcal{m\in}\mathbb{M}} \boldsymbol{v}_{\mathcal{m}}\left( \nu\right)\boldsymbol{v}_{\mathcal{m}}^{\dagger}\left( \nu\right)$

The residuals $\boldsymbol{\xi}_{\mathcal{m}}^{\left( k \right)}\left( \nu\right)$ are determined in the previous iteration in terms of the iteratively linear residual transfer operator $\mathbf{T}_{\boldsymbol{\xi v}}^{\left( k \right)}$

$\boldsymbol{\xi}_{\mathcal{m}}^{\left( k \right)}\left( \nu\right)=\mathbf{T}_{\boldsymbol{\xi v}}^{\left( k \right)}\boldsymbol{v}_{\mathcal{m}}\left( \nu\right)$ (4.39)

The residual transfer operator is defined upon fixed values of the hyperparameters and the source transfer operator $\mathbf{T}_{\boldsymbol{\iota v}}^{\left( k \right)}$:

$\mathbf{T}_{\boldsymbol{\xi v}}^{\left( k \right)}=\mathbf{I}\mathbf{-}\mathbf{L}_{\boldsymbol{v\iota}}\mathbf{T}_{\boldsymbol{\iota v}}^{\left( k \right)}$ (4.40)

This simplifies the computations of ${\bar{\boldsymbol{\Sigma}}}_{\boldsymbol{\xi\xi}}^{\left( k \right)}$ expressed as:

${\bar{\boldsymbol{\Sigma}}}_{\boldsymbol{\xi\xi}}^{\left( k \right)}=\mathbf{T}_{\boldsymbol{\xi v}}^{\left( k \right)}{\hat{\boldsymbol{\Sigma}}}_{vv}\mathbf{T}_{\boldsymbol{v\xi}}^{\left( k \right)}$ (4.41)

The estimation formulas can be derived by applying maximum a posterior of the combined Type II Likelihood and priors. To do so, we reformulate the targeted hyperparameters:

$\rho\leftarrow\frac{\alpha_{1}^{2}}{4\alpha_{2}}$ (4.50)

$\alpha\leftarrow\alpha_{2}$ (4.51)

$\boldsymbol{\Lambda}\leftarrow\frac{1}{2\alpha}\boldsymbol{\Lambda}$ (4.52)

***Variances***

First the estimator of variances $\boldsymbol{\Lambda}\left( g,g \right)\boldsymbol{=\lambda}\left( g \right)\boldsymbol{=}\lambda_{a},g\in\mathbb{G}_{a}, \mathcal{a\in}\mathbb{A}$ can be computed from the stationary values of the expression:

$\left\{ \begin{matrix} \frac{\partial}{\partial\lambda_{a}}\log\left( p^{\left( k \right)}\left( \left\{ \boldsymbol{v}_{m}\left( \nu\right), m\in\mathbb{M}, \nu\in\mathbb{F}_{b} \right\} \right)p\left( \gamma_{a} \right) \right)=0 \\ or \\ \begin{matrix} \frac{\partial}{\partial\lambda_{a}}\left( f_{b}m\log\left| \pi\mathbf{P} \right|-\frac{f_{b}m}{2}tr\left( 2\alpha^{\left( k \right)}\boldsymbol{\Lambda}^{-1}\mathbf{DN}{\bar{\boldsymbol{\Sigma}}}_{\boldsymbol{\iota\iota}}^{\left( k \right)}\left( \mathbf{DN} \right)^{\mathcal{T}} \right) \right) \\ -\frac{1}{2}\frac{\partial}{\partial\lambda_{a}}\log\lambda_{a}+\frac{\partial}{\partial\lambda_{a}}\log p\left( \gamma_{a} \right)=0 \end{matrix} \end{matrix} \right.$ (4.53)

Due to the chain rule of matrix derivatives, the first term can be expressed in a close form.

$$\frac{\partial}{\partial\lambda_{a}}\left( f_{b}m\log\left| \pi\mathbf{P} \right|-\frac{f_{b}m}{2}tr\left( 2\alpha^{\left( k \right)}diag\left( \boldsymbol{\lambda}^{-1} \right)\mathbf{DN}{\bar{\boldsymbol{\Sigma}}}_{\boldsymbol{\iota\iota}}^{\left( k \right)}\left( \mathbf{DN} \right)^{\mathcal{T}} \right) \right)=$$

$\sum_{g\in\mathbb{G}_{a}} \frac{\partial}{\partial\boldsymbol{\lambda}\left( g \right)}\left( f_{b}m\log\left| \pi\mathbf{P} \right|-\frac{f_{\mathcal{b}}m}{2}tr\left( 2\alpha^{\left( k \right)}diag\left( \boldsymbol{\lambda}^{-1} \right){\tilde{\bar{\boldsymbol{\Sigma}}}}_{\boldsymbol{\iota\iota}}^{\left( k \right)} \right) \right)\frac{\partial\boldsymbol{\lambda}\left( g \right)}{\partial\lambda_{a}}$

$\left\{ \begin{matrix} \frac{\partial}{\partial\boldsymbol{\lambda}\left( g \right)}\left( f_{b}m\log\left| \pi\mathbf{P} \right|-\frac{f_{\mathcal{b}}m}{2}tr\left( 2\alpha^{\left( k \right)}diag\left( \boldsymbol{\lambda}^{-1} \right){\tilde{\bar{\boldsymbol{\Sigma}}}}_{\boldsymbol{\iota\iota}}^{\left( k \right)} \right) \right)= \\ or \\ f_{b}m\alpha^{\left( k \right)}\boldsymbol{\lambda}^{-2}\left( g \right)\left( {\tilde{\mathbf{P}}}^{\left( k \right)}\left( g,g \right)+{\tilde{\bar{\boldsymbol{\Sigma}}}}_{\boldsymbol{\iota\iota}}^{\left( k \right)}\left( g,g \right) \right) \end{matrix} \right.$ (4.54)

where ${\tilde{\bar{\boldsymbol{\Sigma}}}}_{\boldsymbol{\iota\iota}}^{\left( k \right)}=\mathbf{DN}{\bar{\boldsymbol{\Sigma}}}_{\boldsymbol{\iota\iota}}^{\left( k \right)}\left( \mathbf{DN} \right)^{\mathcal{T}}$ and ${\tilde{\mathbf{P}}}^{\left( k \right)}=\mathbf{DN}\mathbf{P}^{\left( k \right)}\left( \mathbf{DN} \right)^{\mathcal{T}}$

$\frac{\partial\boldsymbol{\lambda}\left( g \right)}{\partial\lambda_{a}}=\left\{ \begin{matrix} 1 & ,g\in\mathbb{G}_{\mathcal{a}} \\ 0 & ,otherwise \end{matrix} \right.$

Yielding:

$\frac{\partial}{\partial\lambda_{a}}\left( f_{b}m\log\left| \pi\mathbf{P} \right|-\frac{f_{b}m}{2}tr\left( 2\alpha^{\left( k \right)}\boldsymbol{\Lambda}^{-1}\mathbf{DN}{\bar{\boldsymbol{\Sigma}}}_{\boldsymbol{\iota\iota}}^{\left( k \right)}\left( \mathbf{DN} \right)^{\mathcal{T}} \right) \right)=$

$f_{\mathcal{b}}m\frac{\alpha^{\left( k \right)}}{\lambda_{a}^{2}}\sum_{g\in\mathbb{G}_{a}} \left( {\tilde{\mathbf{P}}}^{\left( k \right)}\left( g,g \right)+{\tilde{\bar{\boldsymbol{\Sigma}}}}_{\boldsymbol{\iota\iota}}^{\left( k \right)}\left( g,g \right) \right)$ (4.55)

The second and third term derivatives are:

$\frac{\partial}{\partial\lambda_{a}}\log\lambda_{a}=\frac{1}{\lambda_{a}}$ (4.56)

$\frac{\partial}{\partial\lambda_{a}}\log p\left( \gamma_{\mathcal{a}} \right)=\frac{\partial}{\partial\lambda_{a}}\log TGa\left( \frac{\rho^{\left( k \right)}}{1-\lambda_{a}}\left| \frac{1}{2},1,\left( \rho^{\left( k \right)},\infty\right) \right. \right)=-\frac{1}{2\left( 1-\lambda_{a} \right)}-\frac{\rho^{\left( k \right)}}{\left( 1-\lambda_{a} \right)^{2}}$ (4.57)

Substituting the derivatives, we obtain:

$$\frac{\partial}{\partial\lambda_{a}}\log\left( p^{\left( k \right)}\left( \left\{ \boldsymbol{v}_{m}\left( \nu\right), m\in\mathbb{M}, \nu\in\mathbb{F}_{b} \right\} \right)p\left( \gamma_{a} \right) \right)=$$

$f_{b}m\frac{\alpha^{\left( k \right)}}{\lambda_{a}^{2}}\sum_{b\in\mathbb{G}_{a}} \left( {\tilde{\mathbf{P}}}^{\left( k \right)}\left( g,g \right)+{\tilde{\bar{\boldsymbol{\Sigma}}}}_{\boldsymbol{\iota\iota}}^{\left( k \right)}\left( g,g \right) \right)-\frac{1}{2}\frac{1}{\lambda_{a}}-\frac{1}{2\left( 1-\lambda_{a} \right)}-\frac{\rho^{\left( k \right)}}{\left( 1-\lambda_{a} \right)^{2}}$ (4.58)

But since it holds that $-\frac{1}{2}\frac{1}{\lambda_{a}}-\frac{1}{2\left( 1-\lambda_{a} \right)}=-\frac{1}{2\lambda_{a}\left( 1-\lambda_{a} \right)}$ the expression is much more compact:

$\frac{\partial}{\partial\lambda_{a}}\log\left( p^{\left( k \right)}\left( \left\{ \boldsymbol{v}_{m}\left( \nu\right), m\in\mathbb{M}, \nu\in\mathbb{F}_{b} \right\} \right)p\left( \gamma_{a} \right) \right)=$

$f_{\mathcal{b}}m\frac{\alpha^{\left( k \right)}}{\lambda_{a}^{2}}\sum_{\mathcal{g\in}\mathbb{G}_{\mathcal{a}}} \left( {\tilde{\mathbf{P}}}^{\left( k \right)}\left( g,g \right)+{\tilde{\bar{\boldsymbol{\Sigma}}}}_{\boldsymbol{\iota\iota}}^{\left( k \right)}\left( g,g \right) \right)-\frac{1}{2\lambda_{a}\left( 1-\lambda_{a} \right)}-\frac{\rho^{\left( k \right)}}{\left( 1-\lambda_{a} \right)^{2}}$ (4.59)

Using the auxiliary quantity $\eta_{a}$ so that $\lambda_{a}=\frac{\eta_{a}}{\left( \rho^{\left( k \right)}+\eta_{a} \right)}$ we obtain:

$f_{b}m \alpha^{\left( k \right)}\left( \rho^{\left( k \right)} \right)^{2}\sum_{g\in\mathbb{G}_{a}} \left( {\tilde{\mathbf{P}}}^{\left( k \right)}\left( g,g \right)+{\tilde{\bar{\boldsymbol{\Sigma}}}}_{\boldsymbol{\iota\iota}}^{\left( k \right)}\left( g,g \right) \right)-\frac{1}{2}\rho^{\left( k \right)}\eta_{a}-\rho^{\left( k \right)}\left( \eta_{a} \right)^{2}=0$ (4.60)

Therefore, the only possible solution for the conditions set by the problem statement and estimator can be obtained from $\eta_{a}$ with:

$\eta_{a}^{\left( k+1 \right)}=-\frac{1}{4}+\sqrt{\frac{1}{16}+f_{b}m \alpha^{\left( k \right)}\rho^{\left( k \right)}\sum_{g\in\mathbb{G}_{a}} \left( {\tilde{\mathbf{P}}}^{\left( k \right)}\left( g,g \right)+{\tilde{\bar{\boldsymbol{\Sigma}}}}_{\boldsymbol{\iota\iota}}^{\left( k \right)}\left( g,g \right) \right)}$ (4.61)

$\lambda_{a}^{\left( k+1 \right)}=\frac{\eta_{a}^{\left( k+1 \right)}}{\left( \rho^{\left( k \right)}+\eta_{a}^{\left( k+1 \right)} \right)}$ (4.62)

***Regularization parameters $\boldsymbol{\rho}$ and*** $\boldsymbol{\alpha}$

The estimator of the regularization parameters $\rho$ and $a$ can be computed from the stationary values of the expression below. The computation follows the same steps as for the variances. See section A5 **Proposition A5-IIc**:

$\left\{ \begin{matrix} \frac{\partial}{\partial\rho}\log p\left( \boldsymbol{\gamma} \right)+\frac{\partial}{\partial r}\log p\left( \rho\right)=0 \\ and \\ \begin{matrix} \frac{\partial}{\partial\alpha}\left( f_{\mathcal{b}}m\log\left| \pi\mathbf{P} \right|-\frac{f_{\mathcal{b}}m}{2}tr\left( 2\alpha{\boldsymbol{\Lambda}^{\left( k \right)}}^{-1}{\tilde{\bar{\boldsymbol{\Sigma}}}}_{\boldsymbol{\iota\iota}}^{\left( k \right)} \right) \right) \\ -\frac{1}{2}\frac{\partial}{\partial\alpha}\log\left| 2\pi\frac{1}{2\alpha}\boldsymbol{\Lambda}^{\left( k \right)} \right|+\frac{\partial}{\partial\alpha}\log p\left( \alpha\right)=0 \end{matrix} \end{matrix} \right.$ (4.63)

Writing explicitly the $TGa$ distribution and using the chain rule in the derivative $\frac{\partial}{\partial r}\log p\left( \boldsymbol{\gamma} \right)$:

$\frac{\partial}{\rho}\log p\left( \boldsymbol{\gamma} \right)=$

$\sum_{a\in\mathbb{A}} \left( \frac{\partial}{\partial\gamma_{a}}\log Ga\left( \gamma_{a}\left| \frac{1}{2},1,\left( \rho,\infty\right) \right. \right)\frac{\partial\gamma_{a}}{\partial\rho} \right.\left. -\frac{\partial}{\partial\rho}\log\int_{\rho}^{\infty} Ga\left( x | \frac{1}{2},1 \right)dx \right)$ (4.64)

where $\frac{\partial}{\partial\gamma_{a}}\log Ga\left( \gamma_{a}\left| \frac{1}{2},1,\left( \rho,\infty\right) \right. \right)=-\frac{1-\lambda_{a}^{\left( k \right)}}{2\rho}-1$; $\frac{\partial\gamma_{\mathcal{a}}}{\partial\rho}=\frac{1}{1-\lambda_{a}^{\left( k+1 \right)}}$

$\frac{\partial}{\partial\rho}\log\int_{\rho}^{\infty} Ga\left( x | \frac{1}{2},1 \right)dx=-{Ga\left( \rho| \frac{1}{2},1 \right)}/{\int_{\rho}^{\infty} Ga\left( x | \frac{1}{2},1 \right)dx}$

The derivative $\frac{\partial}{\partial\rho}\log p\left( \rho\right)$ is given by:

$\frac{\partial}{\partial\rho}\log p\left( \rho\right)=\frac{s_{\rho}}{\rho}-r_{\rho}$ (4.65]

where $p\left( \rho\right)\propto Ga\left( \rho\left| s_{\rho}+1,r_{\rho} \right. \right)$ with shape $\left( s_{\rho}+1 \right)$ and rate $r_{\rho}$

From this, we obtain the equation for $\rho$:

$\rho^{\left( k+1 \right)}={zero}_{\rho}\left( -\sum_{\mathcal{a}\in\mathbb{A}} \frac{1}{1-\lambda_{a}^{\left( k+1 \right)}}+l\frac{Ga\left( \rho| \frac{1}{2},1 \right)}{\int_{\rho}^{\infty} Ga\left( x | \frac{1}{2},1 \right)dx}+\left( s_{\rho}-\frac{l}{2} \right)\frac{1}{\rho}-r_{\rho} \right)$ (4.66)

Due to the chain rule of matrix derivatives, the first term for the parameter $\alpha$ can be expressed as:

$$\frac{\partial}{\partial\alpha}\left( f_{\mathcal{b}}m\log\left| \pi\mathbf{P} \right|-\frac{f_{\mathcal{b}}m}{2}tr\left( 2\alpha{\boldsymbol{\Lambda}^{\left( k \right)}}^{-1}{\tilde{\bar{\boldsymbol{\Sigma}}}}_{\boldsymbol{\iota\iota}}^{\left( k \right)} \right) \right)=$$

$\sum_{a\in\mathbb{A}} \frac{1}{\lambda_{a}^{\left( k \right)}}\sum_{g\in\mathbb{G}_{a}} f_{b}m\left( {\tilde{\mathbf{P}}}^{\left( k \right)}\left( g,g \right)+{\tilde{\bar{\boldsymbol{\Sigma}}}}_{\boldsymbol{\iota\iota}}^{\left( k \right)}\left( g,g \right) \right)$ (4.67)

The derivative $\frac{\partial}{\partial\alpha}\log\left| 2\pi\frac{1}{2\alpha}\boldsymbol{\Lambda}^{\left( k \right)} \right|$ is given by:

$\frac{\partial}{\partial\alpha}\log\left| 2\pi\frac{1}{2\alpha}\boldsymbol{\Lambda} \right|=\frac{l}{2\alpha}$ (4.68)

where $l$ is the number of active sources.

The derivative $\frac{\partial}{\partial\rho}\log p\left( \rho\right)$ is given by:

$\frac{\partial}{\partial\alpha}\log p\left( \alpha\right)=\frac{s_{\alpha}}{\alpha}-r_{\alpha}$ (4.69)

where $p\left( \alpha\right)\propto Ga\left( \alpha\left| \left( s_{\alpha}+1 \right),r_{\alpha} \right. \right)$ with shape $\left( s_{\alpha}+1 \right)$ and rate $r_{\alpha}$

From this, we obtain the equation for $\alpha$:

$-f_{b}m\sum_{a\in\mathbb{A}} \frac{\sum_{g\in\mathbb{G}_{a}} \left( {\tilde{\mathbf{P}}}^{\left( k \right)}\left( g,g \right)+{\tilde{\bar{\boldsymbol{\Sigma}}}}_{\boldsymbol{\iota\iota}}^{\left( k \right)}\left( g,g \right) \right)}{\lambda_{a}^{\left( k \right)}}+\frac{l}{2\alpha}+\frac{s_{\alpha}}{\alpha}-r_{\alpha}=0$ (4.70]

*or*

$\alpha^{\left( k+1 \right)}=\left( s_{\alpha}+\frac{l}{2} \right)/\left( f_{b}m\sum_{a\in\mathbb{A}} \frac{\sum_{g\in\mathbb{G}_{a}} \left( {\tilde{\mathbf{P}}}^{\left( k \right)}\left( g,g \right)+{\tilde{\bar{\boldsymbol{\Sigma}}}}_{\boldsymbol{\iota\iota}}^{\left( k \right)}\left( g,g \right) \right)}{\lambda_{a}^{\left( k \right)}}+r_{\alpha} \right)$ (4.71)

***Noise parameter*** $\boldsymbol{\sigma}_{\boldsymbol{\xi}}^{\boldsymbol{2}}$

The estimator of the noise parameter $\beta$ can be computed from the stationary values of the expression below, following the same steps as for the variances.

$\frac{\partial}{\partial\beta}\left( f_{b}m\log\left| \pi\mathbf{P} \right|-f_{b}m\beta^{-1}tr\left( {\bar{\boldsymbol{\Sigma}}}_{\boldsymbol{\xi\xi}}^{\left( k \right)} \right) \right)-f_{b}m\frac{\partial}{\partial\beta}\log\left| \pi\beta\mathbf{I} \right|+\frac{\partial}{\partial\beta}\log p\left( \beta\right)=0$ (4.72)

Due to the chain rule of matrix derivatives, the first term for the parameter $\beta$ can be expressed as:

$$\frac{\partial}{\partial\beta}\left( f_{b}m\log\left| \pi\mathbf{P} \right|-f_{b}m\beta^{-1}tr\left( {\bar{\boldsymbol{\Sigma}}}_{\boldsymbol{\xi\xi}}^{\left( k \right)} \right) \right)=$$

$f_{\mathcal{b}}m \sigma_{\boldsymbol{\xi}}^{-4}\sum_{\mathcal{e\in}\mathbb{E}} \left( {\check{\boldsymbol{\Sigma}}}_{\boldsymbol{\xi\xi}}^{\left( k \right)}\left( \mathcal{e}\mathcal{,e} \right)+{\bar{\boldsymbol{\Sigma}}}_{\boldsymbol{\xi\xi}}^{\left( k \right)}\left( s,s \right) \right)$ (4.73)

where ${\check{\boldsymbol{\Sigma}}}_{\boldsymbol{\xi\xi}}^{\left( k \right)}=\mathbf{L}_{\boldsymbol{v\iota}}{\check{\boldsymbol{\Sigma}}}_{\boldsymbol{\iota\iota}}^{\left( k \right)}\mathbf{L}_{\boldsymbol{\iota v}}$ and $\mathbf{S}_{\boldsymbol{\xi\xi}}^{\left( k \right)}={\check{\mathbf{T}}}_{\boldsymbol{\xi v}}^{\left( k \right)}\mathbf{S}_{\boldsymbol{vv}}{\check{\mathbf{T}}}_{\boldsymbol{v\xi}}^{\left( k \right)}$ with ${\check{\mathbf{T}}}_{\boldsymbol{\xi v}}^{\left( k \right)}=\mathbf{I-}\mathbf{L}_{\boldsymbol{v\iota}}{\check{\mathbf{T}}}_{\boldsymbol{\iota v}}^{\left( k \right)}$

The derivative $\frac{\partial}{\partial\sigma_{\boldsymbol{\xi}}^{2}}\log\left| \pi\sigma_{\boldsymbol{\xi}}^{2}\mathbf{I} \right|$ is given by:

$\frac{\partial}{\partial\sigma_{\boldsymbol{\xi}}^{2}}\log\left| \pi\sigma_{\boldsymbol{\xi}}^{2}\mathbf{I} \right|=\frac{p}{\sigma_{\boldsymbol{\xi}}^{2}}$ (4.74)

The derivative $\frac{\partial}{\partial\sigma_{\boldsymbol{\xi}}^{2}}\log p\left( \sigma_{\boldsymbol{\xi}}^{2} \right)$ is given by:

$\frac{\partial}{\partial\sigma_{\boldsymbol{\xi}}^{2}}\log p\left( \sigma_{\boldsymbol{\xi}}^{2} \right)=-\frac{s_{\boldsymbol{\xi}}}{\sigma_{\boldsymbol{\xi}}^{2}}+\frac{r_{\boldsymbol{\xi}}}{\sigma_{\boldsymbol{\xi}}^{4}}$ (4.75)

where $p\left( \sigma_{\boldsymbol{\xi}}^{2} \right)\propto Ga\left( 1/{\sigma_{\boldsymbol{\xi}}^{2}}\left| \left( s_{\boldsymbol{\xi}}+1 \right),r_{\boldsymbol{\xi}} \right. \right)$ with shape $\left( s_{\boldsymbol{\xi}}+1 \right)$ and rate $r_{\boldsymbol{\xi}}$

From this, we obtain the equation for $\sigma_{\boldsymbol{\xi}}^{2}$:

$f_{\mathcal{b}}m \sigma_{\boldsymbol{\xi}}^{-4}\sum_{\mathcal{e\in}\mathbb{E}} \left( {\check{\boldsymbol{\Sigma}}}_{\boldsymbol{\xi\xi}}^{\left( k \right)}\left( \mathcal{e}\mathcal{,e} \right)+\mathbf{S}_{\boldsymbol{\xi\xi}}^{\left( k \right)}\left( \mathcal{e}\mathcal{,e} \right) \right)-f_{\mathcal{b}}m\frac{p}{\sigma_{\boldsymbol{\xi}}^{2}}-\frac{s_{\boldsymbol{\xi}}}{\sigma_{\boldsymbol{\xi}}^{2}}+\frac{r_{\boldsymbol{\xi}}}{\sigma_{\boldsymbol{\xi}}^{4}}=0$ (4.76)

*or*

${\sigma_{\boldsymbol{\xi}}^{2}}^{\left( k+1 \right)}=\left( s_{\boldsymbol{\xi}}+f_{\mathcal{b}}\mathrm{mp} \right)/\left( f_{\mathcal{b}}m\sum_{\mathcal{e\in}\mathbb{E}} \left( {\check{\boldsymbol{\Sigma}}}_{\boldsymbol{\xi\xi}}^{\left( k \right)}\left( \mathcal{e}\mathcal{,e} \right)+\mathbf{S}_{\boldsymbol{\xi\xi}}^{\left( k \right)}\left( \mathcal{e}\mathcal{,e} \right) \right)+r_{\boldsymbol{\xi}} \right)$ (4.77)

**Algorithm statistics:** The ssSBL allows screening out the neural space by thresholding the posterior distribution statistic: the ratio of the posterior mean and posterior variances. After convergence, the estimated source activity can be thresholded employing an unbiased statistic: this is due to the posterior distribution of source activity $N_{q}^{\mathbb{C}}\left( \boldsymbol{\iota} | \boldsymbol{\mu},\mathbf{P} \right)$ of formula [5.1], where the quantities $\hat{\boldsymbol{\iota}}$ (posterior mean) and ${\check{\boldsymbol{\Sigma}}}_{\boldsymbol{\iota\iota}}$ (posterior covariance) are defined as follows:

$\boldsymbol{\mu}=\beta^{-1}\mathbf{P}\mathbf{L}_{\boldsymbol{\iota v}}\boldsymbol{v}$ (4.78)

$\mathbf{P}=\left( \beta^{-1}\mathbf{L}_{\boldsymbol{\iota v}}\mathbf{L}_{\boldsymbol{v\iota}}+\frac{1}{2}\left( \mathbf{DN} \right)^{\mathcal{T}}\boldsymbol{\Lambda}^{-1}\mathbf{DN} \right)^{-1}$ (4.79)

In this distribution, $diag\left( \mathbf{S}_{\boldsymbol{\iota\iota}} \right)$ is the posterior mean and ${\check{\boldsymbol{\Sigma}}}_{\boldsymbol{\iota\iota}}$ the posterior covariance. The z-statistic for the analysis of variance has the following form:$z_{stat}=sqrt\left( {diag\left( \mathbf{S}_{\boldsymbol{\iota\iota}} \right).}/{diag\left( {\check{\boldsymbol{\Sigma}}}_{\boldsymbol{\iota\iota}} \right)} \right)$. A plausible way to screen out the active sources is to extract the set of nodes $\mathcal{I}$ that return a value of the z-statistic greater than 1: $\mathbb{G}_{0}=\left\{ \boldsymbol{\iota}:{z\_stat}_{\iota}\geq1 \right\}$.

**Implementation details:** The high computational cost for obtaining $\bar{\Sigma}_{t}$ employing a matrix inversion operation can be avoided by using the economic singular value decomposition (SVD) of the lead field $\mathbf{L}_{\boldsymbol{v\iota}}=\mathbf{LS}\mathbf{R}^{\mathcal{T}}$, and the Woodbury identity, leading to: $\mathbf{P}=\beta\boldsymbol{\Lambda R}\left( \mathbf{R}^{\mathcal{T}}\boldsymbol{\Lambda R}+\beta\mathbf{S}^{-2} \right)^{-1}\mathbf{S}^{-2}\mathbf{R}^{\mathcal{T}}$.

The update formulas in Proposition A5-II a), b) are consistent with the sparsity constraint in both the ENET and ELASSO models, since the elements of the effective prior variance matrix $\boldsymbol{\Lambda}$ (or equivalently $\tilde{\boldsymbol{\Lambda}}$**)** select which elements of $\mu$ become zero. When $\boldsymbol{\Lambda}\left( i,i \right)\to0$, the $i$-th row and $i$-th -column of the matrix $\mathbf{P}$ tend to zero vectors, from where $\hat{\boldsymbol{\iota}}\left( i \right)\to0$. In the same way, if some parameters are very small in a previous iteration ($\boldsymbol{\mu}\left( i \right)\approx0$, i-th diagonal element $\mathbf{P}\left( i,i \right)\approx0$), they will lead to $\boldsymbol{\Lambda}\left( i,i \right)\to0$ in the next iteration (equations (A5-2) and (A5-8)). In some algorithms, this property usually means that if one activation is set to zero (e.g., removed from the active set) in an iteration, it will not appear as part of the solution. In our case, however, we do not prune to zero the small coefficients. Therefore, although unlikely, a “zeroed” activation might be re-estimated in a future iteration and contribute to the solution.

The nonlinear terms in (A5-5) and (A5-10) are obtained from the derivative of the normalization constants. These terms decrease strictly with respect to their arguments leading to smaller values of F for higher values of $\rho$ and $\alpha$, which is equivalent in both cases to have more zero elements in $\tilde{\boldsymbol{\Lambda}}$. The measurements variance $\beta$ in (A5-6) and (A5-7) is generally considered superfluous in the learning process, because it only acts as a scale factor for the parameters and usually decelerates the algorithm convergence. In our case, we fix it to $\beta=1$ , for all time points.

We also use fixed values for the parameters of the Gamma distribution of ENET’s $\rho$ and $\alpha$ and ELASSO’s $\alpha$. In particular, we chose for ENET $s_{\rho}=q$, which preserves the monotony of (C6-5) (in the sense that only one zero of $F$ exists) and $r_{\rho}=\epsilon q$, where $\epsilon$ is such that the prior is flexible: with a mean ($\rho\left( t \right)$)$\approx1/\epsilon$ and variance ($\rho\left( t \right)$)$\approx1/\left( \epsilon^{2}q \right)$. Following a similar flexible strategy with a mean ($\alpha\left( t \right)$)$\approx1/\epsilon$ and variance ($\alpha\left( t \right)$)$\approx1/\left( \epsilon^{2}q \right)$, we chose ENET’s $s_{\alpha}=q/2$, which is also in the same order of magnitude of the numerator in (C6-3), and $r_{\alpha}=\epsilon q$, as a form of regularization in the denominator of (C6-3). This combination of similar $\rho$ and $\alpha$ priors keeps adequate balance, allowing flexibility in our learning of different degrees of sparsity. Our choice of ELASSO’s $\alpha$ is similar to that of the ENET’s $s_{\rho}=\mathrm{qT}$, which preserves the monotony of (C6-10) (in the sense that only one zero of $F$ exists) and $r_{\rho}=\epsilon\mathrm{qT}$, where $\epsilon$ is such that the prior is flexible: with a mean ($\alpha\left( t \right)$)$\approx1/\epsilon$ and variance ($\alpha\left( t \right)$)$\approx1/\left( \epsilon^{2}\mathrm{qT} \right)$.

References

Arnold, Ludwig. 1974. “Stochastic Differential Equations: Theory and Application.”

———. 1995. “Random Dynamical Systems.” *Dynamical Systems*, 1–43.

Capiński, Marek, and Peter Ekkehard Kopp. 2004. *Measure, Integral and Probability*. Vol. 14. Springer.

Damoiseaux, Jessica S, SARB Rombouts, Frederik Barkhof, Philip Scheltens, Cornelis J Stam, Stephen M Smith, and Christian F Beckmann. 2006. “Consistent Resting-State Networks across Healthy Subjects.” *Proceedings of the National Academy of Sciences* 103 (37): 13848–53.

Durrett, Rick. 2019. *Probability: Theory and Examples*. Vol. 49. Cambridge university press.

Essen, David C. Van, Stephen M. Smith, Deanna M. Barch, Timothy E.J. Behrens, Essa Yacoub, and Kamil Ugurbil. 2013. “The WU-Minn Human Connectome Project: An Overview.” *NeuroImage* 80: 62–79. https://doi.org/10.1016/j.neuroimage.2013.05.041.

Greicius, Michael. 2008. “Resting-State Functional Connectivity in Neuropsychiatric Disorders.” *Current Opinion in Neurology* 21 (4): 424–30.

Hui-Hsiung, Kuo. 2006. *Introduction to Stochastic Integration*. Universitext. New York: Springer-Verlag. https://doi.org/10.1007/0-387-31057-6.

Larson-Prior, L. J., R. Oostenveld, S. Della Penna, G. Michalareas, F. Prior, A. Babajani-Feremi, J. M. Schoffelen, et al. 2013. “Adding Dynamics to the Human Connectome Project with MEG.” *NeuroImage* 80: 190–201. https://doi.org/10.1016/j.neuroimage.2013.05.056.

Lehmann, Erich L, and George Casella. 2006. *Theory of Point Estimation*. Springer Science & Business Media.

Smith, Stephen M, Diego Vidaurre, Christian F Beckmann, Matthew F Glasser, Mark Jenkinson, Karla L Miller, Thomas E Nichols, Emma C Robinson, Gholamreza Salimi-Khorshidi, and Mark W Woolrich. 2013. “Functional Connectomics from Resting-State FMRI.” *Trends in Cognitive Sciences* 17 (12): 666–82.

Steele, J Michael. 2001. *Stochastic Calculus and Financial Applications*. Vol. 1. Springer.
